# Synthesis, Characterization, and Electrochemical Studies of Fused Oxazolidine Complexes of Molybdenum

**DOI:** 10.1021/acsomega.6c05562

**Published:** 2026-07-10

**Authors:** Jiayun Li, Aurodeep Panda, William W. Brennessel, William D. Jones

**Affiliations:** Department of Chemistry, University of Rochester, Rochester, New York 14627, United States

## Abstract

Two fused-bisoxazolidine ligands (FOX^OH^ and FOX^OMe^) are used to coordinate to molybdenum carbonyl, producing Mo­(CO)_3_(FOX^OR^). The ligands bind in a κ^3^-*NNN* fashion to the metal center creating an octahedral complex. Difficulty in further substitution of CO and the lack of reactivity prompted research into alternative starting materials. This resulted in the synthesis of a MoCl_3_(FOX^OMe^) complex. All complexes are characterized by single-crystal X-ray crystallography and cyclic voltammetry.

## Graphical abstract

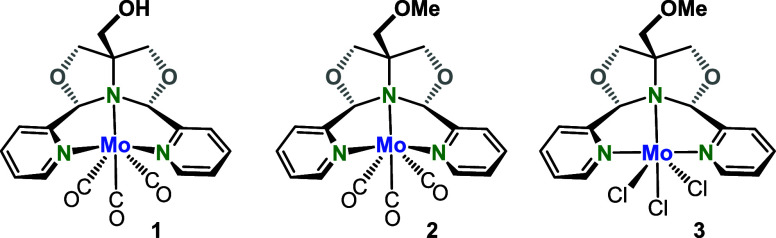

## Introduction

Fused oxazolidine (FOX) compounds have had a long history since their discovery in 1945.^1^ They have been the focus of many biological and pharmaceutical research studies over the past few decades.^2^ In 2021, Jones *et al.* reported a series of metal complexes created with FOX ligands and first-row transition metals.^3^ In the same year, it was discovered that the iron triflate FOX exhibits catalytic potential for alcohol dehydration.^4^ A Cu^II^(FOX^OH^) complex was found to efficiently dehydrate 1-phenylethanol to styrene.^5^ Besides the works by Jones, there have been few studies regarding FOX compounds as ligands in metal complexes. One published study in the past decade was by Das *et al.* in which a dinuclear iron-μ-oxo complex with FOX ligands was used to catalyze water oxidation reactions.^6^ Another more recent example was published by Sanchez-Portillo and co-workers who discovered that a Zn^II^(FOX) complex can be produced when ZnCl_2_ is present during the condensation reaction between aminodiols and carboxaldehydes.^2^

FOX falls under the general category of NNN pincer ligands, which has been a group of interest for some time. This is due to the fact that pincer ligands give the metal complex more stability, leading to less metal leaching during homogeneous catalytic reactions.^7,8^ One well-known example is pyridine-2,6-bis­(oxazoline) (PyBOX). PyBOX is a derivative of BOX (BOX = bis­(oxazoline)­methane), with pyridine as a spacer between the two oxazolines. Since its discovery in 1989, PyBOX has received much attention and has become an important catalyst for organic synthesis.^9^ FOX, also being an NNN tridentate ligand with oxazolidine and pyridine elements, could potentially hold similar properties and might also offer flexibility with its derivatives. In this contribution, we aim to further understand the underlying properties of FOX ligands and their potential as polydentate ligands for transition metals.

After the extensive exploration of the binding of first-row transition metals with FOX,^3^ we have initiated studies with second-row transition metals. Molybdenum was chosen for several reasons: it has several common oxidation states and is known to form complexes with a wide range of coordination numbers; it is also relatively inexpensive and environmentally friendly unlike some of the other second-row transition metals.^10^ These properties of molybdenum made it an attractive choice for metal complex research. Thus, research into molybdenum complexes and specifically tridentate and pincer-type ligands is quite abundant.^11^ Yet, the catalytic capacity of these compounds, specifically NNN pincer molybdenum complexes, has not been widely reported. There is limited literature on molybdenum NNN pincer ligands.^12–16^ Therefore, this research aims to explore reactivity of NNN molybdenum complexes containing the FOX ligand.

## Results and Discussion

### FOX Ligands Used in This Study

The FOX ligands used were the L1_py_^meso^ type as described previously by Jones *et al.* in 2021.^3^ Two ligands were explored, FOX^OH^ and FOX^OMe^ (Figure 1). The latter was prepared by reaction of FOX^OH^ with NaH followed by methyl iodide. Reactions were carried out in parallel with both ligands when possible.

**Figure 1:**
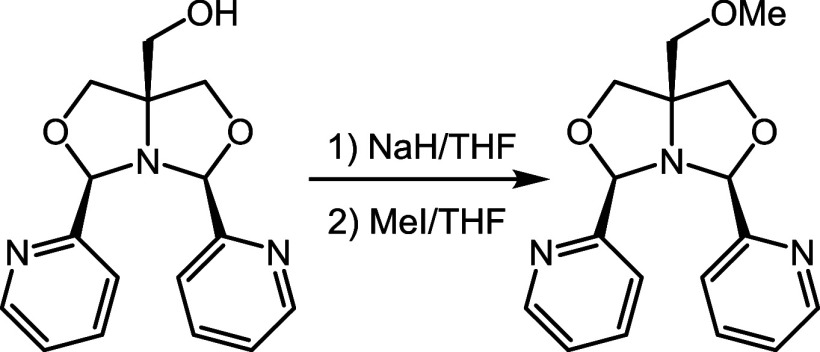
FOX ligands in this study. FOX^OH^ (left) and FOX^OMe^ (right).

### Synthesis and Characterization of Mo^0^ FOX Complexes

The original plan was to test two different synthetic routes to prepare bromide/carbonyl derivatives of the type Mo­(FOX)­(Br)_*n*_(CO)_3–*n*_ (Scheme 1). The Mo­(FOX)­(Br)_*n*_(CO)_3–*n*_ could then be used to create hydride complexes, or carry out catalytic reactions on its own. Partial success with two of these routes is described below.

**Scheme 1:**
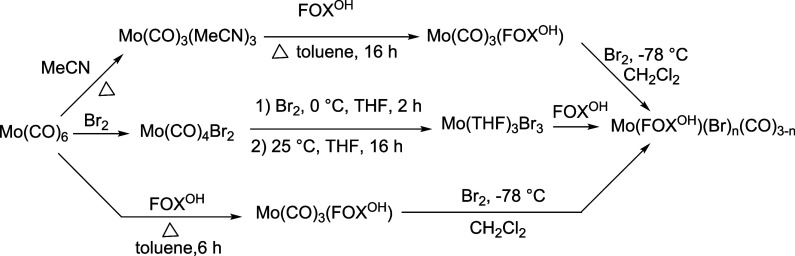
Three Proposed Synthetic Routes for Mo­(Br)_*n*_(CO)_3–*n*_(FOX) Complexes

Route one was first attempted with some success. Mo­(CO)_3_(FOX^OH^) (**1**) and Mo­(CO)_3_(FOX^OMe^) (**2**) were obtained after reacting the corresponding FOX ligand with the known intermediate Mo­(CO)_3_(MeCN)_3_ in refluxing toluene (Scheme 1, top).^17^ This reaction was further simplified into a one-step reaction as seen at the bottom of Scheme 1. These diamagnetic compounds were characterized by ^1^H NMR and IR spectroscopies and elemental analysis (Figure 2).

**Figure 2:**
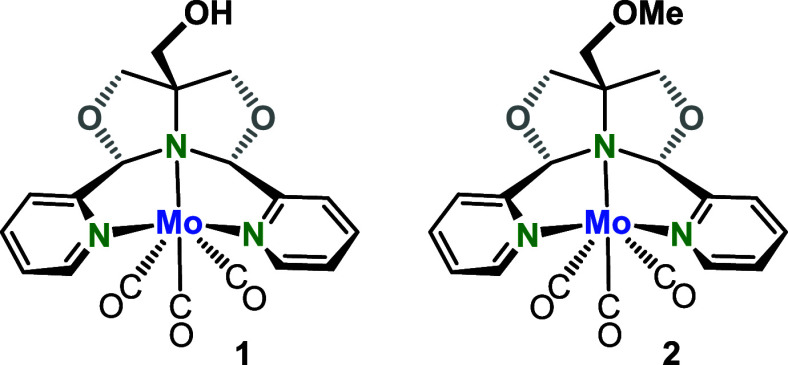
Mo^0^ FOX complexes. *fac*-Mo­(CO)_3_(FOX^OH^) (left) and *fac*-Mo­(CO)_3_(FOX^OMe^) (right).

The single-crystal X-ray structures of **1** and **2** were determined (Figures 3 and 4). Each shows the FOX ligand in a *fac*-κ^3^-*NNN* bonding mode. By comparison, previous FOX complexes with first-row metals were all seen to coordinate in a *mer*-fashion (15 examples).^3^ Only [Re­(FOX)­(CO)_3_]^+^ has been seen in a *fac*-coordination mode, and it may be that a larger metal binds preferably in a facial manner.^18^ Complex **1** crystallizes in space group *P2*_1_ */c* with *Z* = 8, indicating two independent molecules within the asymmetric unit. One of these showed disorder in the binding of the FOX^OH^ ligand over two positions, so only the ordered molecule is shown here and discussed. Interestingly, two of the Mo–N bonds are about 0.1 Å shorter than the third (Mo1–N2 = Mo1–N3 = 2.252(5) Å; Mo1–N1 = 2.380(6) Å). These differences can be ascribed to the presence of *sp* ^2^ hybridized nitrogens in the pyridine ligands (N2, N3) but *sp* ^3^ hybridized nitrogen in the bisoxazoline part of the ligand (N1). Two of the Mo–CO bonds are slightly longer than the third (Mo1–C18 = Mo1–C19 = 1.929(8) Å; Mo1–C17 = 1.892(2)), with the shorter Mo–C bond being trans to the longer Mo–N­(*sp* ^3^) bond. The stronger Mo1–C17 interaction also induces a lengthening of the C17–O4 bond, compared to the other CO bond lengths (C17–O4 = 1.175(9) vs C18–O5 = 1.154(9), C19–O6 = 1.153(9)). Similarly, the N–Mo–N and C–Mo–C bond angles are slightly more acute when the “outlier” atom (C17) in the octahedron is involved (N1–Mo1–N2 = 73.2(2), N1–Mo1–N3 = 73.6(2), N2–Mo1–N3 = 86.6(2); C17–Mo1–C18 = 86.7(3), C17–Mo1–C19 = 84.6(3), C18–Mo1–C19 = 88.6(3)). The hydroxymethyl group is seen to be dangling away from the core of the molecule, and hydrogen bonds to the OH group in an adjacent unit cell or to the “outlier” CO in an adjacent molecule, contributing to the asymmetry in the bonding described above. For comparison, a similar *fac*-N_3_Mo­(CO)_3_ complex has been reported with a κ^3^-1,1,1-tris­(pyrid-2-yl)­ethane) ligand. Here, the average Mo–N distances were 2.24 Å and the average Mo–C distances were 1.95 Å, very similar to the corresponding distances observed in **1** and **2**.^19^ Another related example is the complex (bis­(picolyl)­amine)­Mo­(CO)_3_, for which the Mo–N and Mo–C distance averages are 2.25 Å and 1.91, respectively, also very similar to the corresponding distances observed in **1** and **2**.^15^

**Figure 3:**
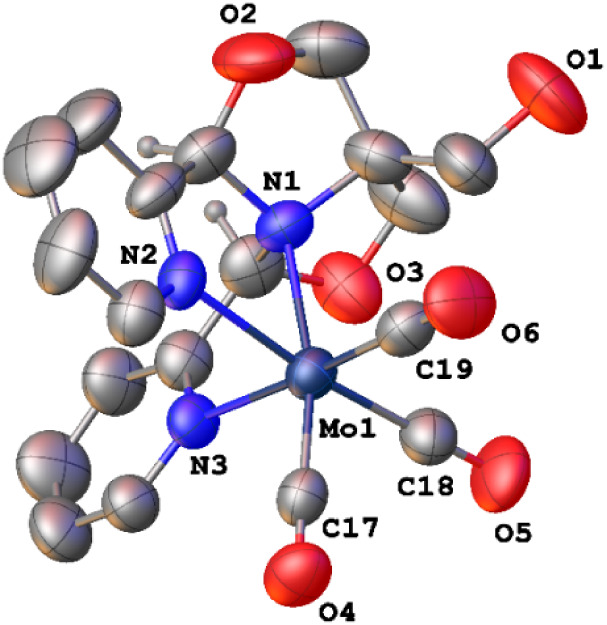
Thermal ellipsoid structure of **1** at 20 °C (50% probability ellipsoids). Most hydrogens have been omitted for clarity.

**Figure 4:**
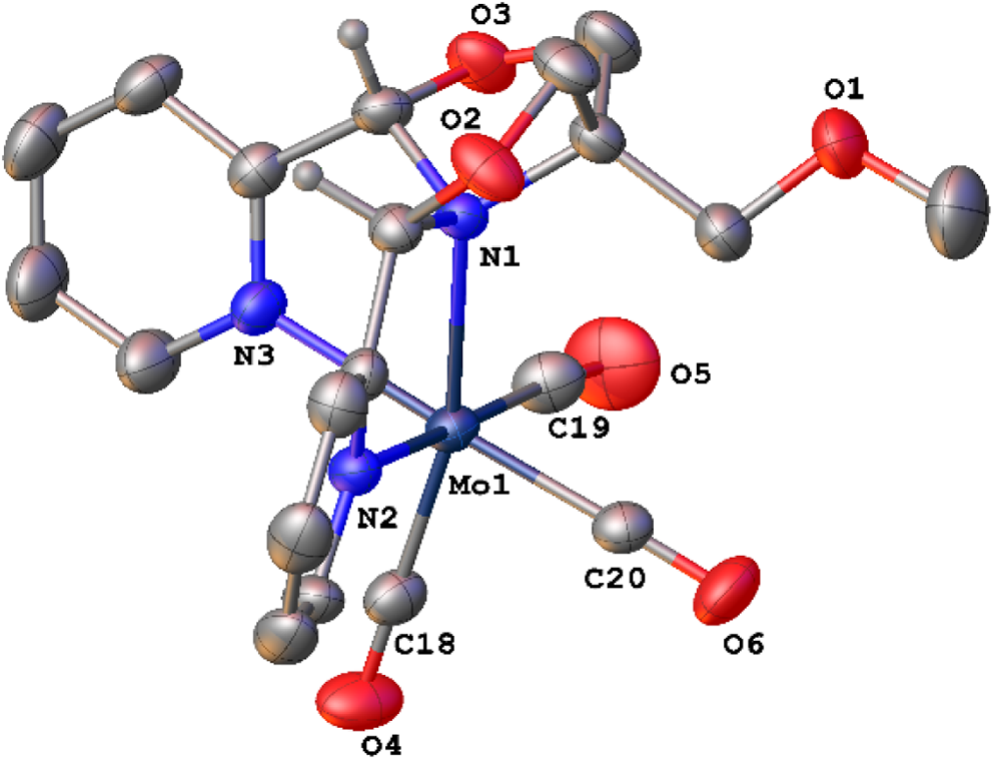
Thermal ellipsoid structure of **2** at 20 °C (50% probability ellipsoids). Most hydrogens have been omitted for clarity.

Molecule **2** also crystallizes in space group *P2*_1_ */c* but with *Z* = 4 (one unique molecule per unit cell). As in compound **1**, the two Mo–N­(*sp* ^2^) bonds are shorter than the third Mo–N­(*sp* ^3^) bond (Mo1–N2 = 2.2612(14), Mo1–N3 = 2.2523(15), Mo1–N1 = 2.3787(14)) and two of the Mo–CO bonds are longer than the third, but the differences for the latter are smaller (0.01–0.02 Å). The C–O bond lengths are also within 0.01 Å of each other. No hydrogen bonding is seen in this methoxymethyl derivative, which appears to contribute to the CO bond asymmetry in **1** but is necessarily absent in **2**.

Both **1** and **2** are low-spin Mo(0) complexes and can be characterized by NMR spectroscopy. Both have *C*_*s*_ symmetry, rendering the two halves of the FOX ligand equivalent. The ^1^H NMR spectrum of **1** displays 4 resonances in the aromatic region for the two equivalent pyridines. Other resonances for the bisoxazoline hydrogens (H3A/H5A, H3B/H5B, H4/H6) and hydroxymethyl group (H1C/H1D, H1A/H1B) are fully resolved (see Supporting Information Figure S1). The ^1^H NMR spectrum for **2** shows a similar pattern of resonances, with an additional singlet at δ3.36 for the OMe group (Figure S7). All resonances are shifted downfield from those in the free ligand.

The ^13^C NMR spectra for **1** and **2** show resonances consistent with *C*_*s*_ symmetry. Five pyridine carbons are observed, along with 4 carbons for the bisoxazoline and CH_2_O moieties. **2** displays an additional resonance for the OMe group at δ 59.68 (Figures S2 and S8).

The infrared spectrum of **1** is dominated by the two CO stretches at 1894(s) and 1745­(br) cm^–1^ as expected for a low-valent tricarbonyl in a *C*_*s*_ symmetric environment (*A*_1_ and *E* bands). Other notable stretches seen are assigned to the C–O single bond (1107 cm^–1^) and O–H bond (3553­(br) cm^–1^). Compound **2** shows carbonyl stretches at 1900(s) and 1742­(br) cm^–1^ for the *A*_1_ and *E* bands and a C–O single bond stretch at 1100 cm^–1^ (Figures S3 and S9).

A UV–vis spectrum of complexes **1** and **2** was recorded in THF solvent and shows 4 absorptions at 254, 312, 366, and 452 nm (Figure 5). These are likely MLCT bands, as the extinction coefficients are on the order of 5000–18,000 M^–1^ cm^–1^. In addition, these compounds are set up for MLCT as they contain a Mo(0) center and strong π-acceptor ligands (CO and pyridine). The spectrum of **3** (*vide infra*) containing Mo­(III) shows similar features, but with smaller extinction.

**Figure 5:**
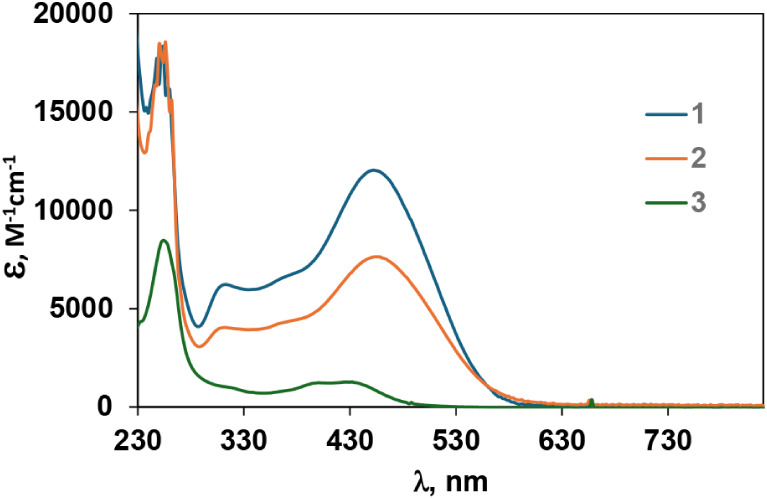
UV–vis spectrum of **1** and **2** in THF (0.125, 0.132 mM) and **3** in CH_2_Cl_2_ (0.093 mM).

Unfortunately, neither complex **1** nor **2** showed further promising reactivity. Attempts to replace any of the remaining carbonyls using photochemical or thermal methods failed.^20^ The CO ligands remained strongly attached. This is not entirely unexpected, as *fac*-Mo­(CO)_3_(1,1,1-tris­(pyrid-2-yl)­ethane) was found to be more photostable than [Ru­(bipy)_3_]^2+^.^19^ Addition of trimethylamine-N-oxide (TMANO) to **2** in THF in the presence of COD showed complete decomposition by IR spectroscopy—no CO ligands remained and free FOX^OMe^ ligand was observed in the ^1^H NMR spectrum. A similar reaction of **2** with TMANO in refluxing acetonitrile showed only free FOX^OMe^. Reaction of **2** with Br_2_ in dichloromethane as proposed in Scheme 1 also led to loss of the FOX^OMe^ ligand. A crude X-ray analysis of crystals obtained from the reaction mixture showed [H_2_FOX^OMe^]^+2^Br^–^ and [MoBr_4_O­(CH_3_CN)]^−^. These observations prompted additional synthetic attempts using Mo­(III) complexes.

### Synthesis and Characterization of Mo­(III) FOX Complex

The route to prepare the MoBr_3_(THF)_3_ complex as laid out in the middle of Scheme 1 was not successful due to difficulties repeating the literature procedure without sufficient detail.^21^ Consequently, a new route was chosen on the basis of a report by Chirik and co-workers for a similar Mo­(III) complex (Scheme 2).^22^ Reduction of MoCl_5_ with tin powder in diethyl ether gave MoCl_4_(Et_2_O)_2_. Further reduction with tin powder in THF yielded MoCl_3_(THF)_3_. Further reaction of MoCl_3_(THF)_3_ with FOX^OMe^ yielded MoCl_3_(FOX^OMe^), **3** (Figure 6). Due to the paramagnetic nature of this product, NMR spectroscopy was not valuable for characterization (Figure S10). Fortunately, the compound crystallized and a single-crystal X-ray structure was obtained (Figure 7).

**Scheme 2:**
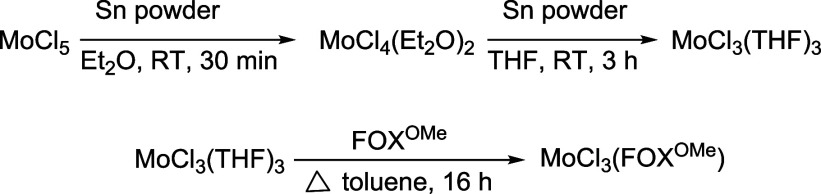
Synthetic Route to MoCl_3_(FOX^OMe^)

**Figure 6:**
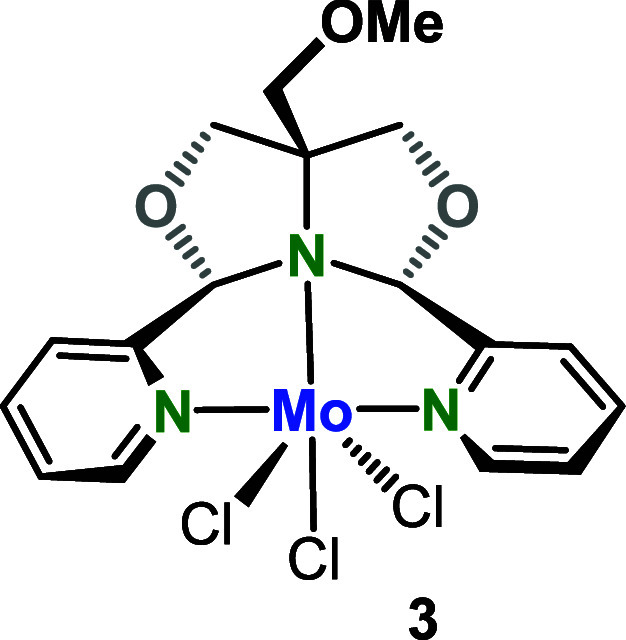
Complex **3**, *mer-*MoCl_3_(FOX^OMe^).

**Figure 7:**
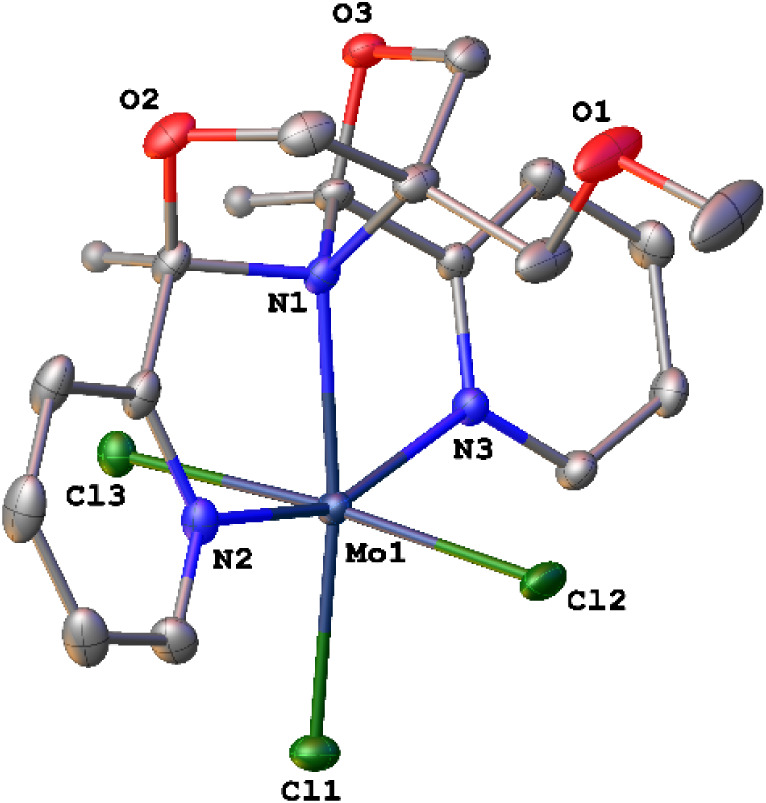
Thermal ellipsoid structure of **3** at −173 °C (50% probability ellipsoids). Hydrogen atoms have been omitted for clarity.

The structure of **3** shows a *mer*-octahedral Mo^III^ geometry. The molecule crystallizes in space group *Pbca* with *Z* = 8, indicating one unique molecule in the asymmetric unit. The Mo–N distances are shorter to the two pyridine groups (2.135(2), 2.1151(2) Å) than to the tertiary *sp* ^3^ nitrogen (2.260(2) Å) as seen in **1** and **2**. In addition, all of the Mo^III^-nitrogen distances are approximately 0.1 Å shorter in **3** than in **1** or **2**, a result of the decreased radius of Mo^III^ vs Mo^0^. The smaller Mo^III^ metal center may permit meridional coordination. Also, the Mo–Cl distances show two short and one long bond (2.4063(5), 2.3968(5), 2.4575(5) Å), however, the long bond is Mo1–Cl3 rather than the bond trans to *sp* ^3^ N1.

In our earlier studies of FOX compounds of first-row metals, the *meso*-FOX^OH^ ligand was always observed to adopt a κ^4^-*NNNO* geometry.^3^ In all complexes **1**–**3**, the HOCH_2_ or MeOCH_2_ portion of the FOX ligand is dangling, and not interacting with the molybdenum. This is likely due to the coordination saturation of the complexes and the lack of lability of the CO or Cl ligands, which also accounts for their lack of reactivity. Molybdenum(0) is sufficiently electron-rich that it binds the remaining three COs tightly, and the *d* ^6^ octahedral configuration adds to the stability. Similarly, molybdenum­(III) binds the chloride ligands tightly, and the *d* ^3^ half-octahedral configuration also adds stability. This same situation was seen with FOX^OH^ binding to a rhenium tricarbonyl cation in which the CH_2_OH group is dangling and the CO groups were not labile.^18^ It might be possible that an analog of **3** with a *meso*-FOX^OH^ ligand could be deprotonated and replace a chloride to give a κ^4^-*NNNO* complex, but this was not investigated here.

Several reactions were attempted with trichloro complex **3**. First, the reduction of MoCl_3_(FOX^OMe^) was attempted using 5% sodium amalgam. The reaction decomposed rapidly into a dark brown solution. Next, alkylation was attempted using LiCH_2_SiMe_3_, but no product was obtained. Similarly, reaction with LiHB­(*sec*-Bu)_3_ gave no tractable product. Several (NNN)­MoX_3_ complexes with NNN-pincer ligands have been reported, but limited reactivity was observed beyond oxidation.^23–30^

### Cyclic Voltammetry of Mo­(FOX) Complexes

The redox properties of complexes **1**–**3** were examined in acetonitrile or DMF solution (**3** was not soluble in acetonitrile). As seen in Figure 8a, complex **1** displays a single irreversible oxidation at −0.18 V (vs Fc), indicating that the complex is not very reducing despite being formally Mo^0^. In Figure 8b, the voltammogram for **2** also shows a single reduction at −0.16 V as methylation of the hydroxymethyl group has little influence on the electronics of the complex. The oxidation likely leads to CO loss as the chemically irreversible step in the cycle as a result of reduced electron density for π-backbonding. In contrast, the voltammogram for **3** shows two quasi-reversible reductions at −1.49 and −1.77 V, which could lead to halide loss.

**Figure 8:**
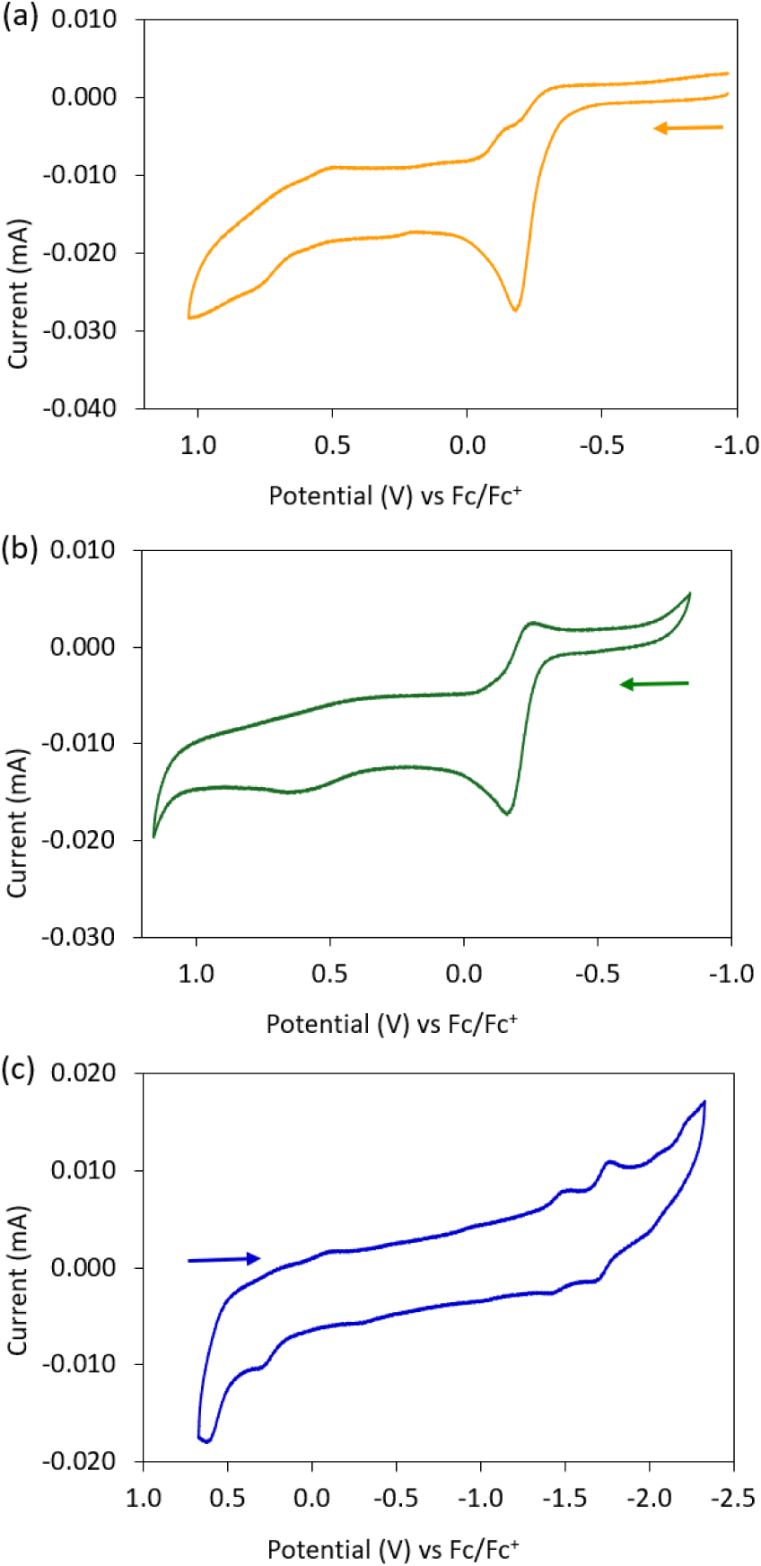
Cyclic voltammograms of (a) 1 mM **1** in CH_3_CN, (b) 1 mM **2** in CH_3_CN, and (c) 1 mM **3** in DMF. 0.01 M ^*n*^Bu_4_PF_6_, 50 mV/s.

## Conclusions

In summary, three new molybdenum FOX complexes were discovered and characterized during the search for potential molybdenum FOX catalysts. Neither of the two molybdenum tricarbonyl complexes showed reactivity involving loss of CO. None of the three complexes showed further reactivity toward alkylation or reaction with borohydride. Cyclic voltammetry studies show irreversible oxidations for the two Mo(0) complexes and quasi-reversible reductions for the Mo­(III) complex.

## Experimental Section

### General Remarks

Unless otherwise specified, all reagents were used directly as purchased from Aldrich, Fisher, or Acros except for dichloromethane (DCM) which was used as received from Mallinckrodt. Tetrahydrofuran (THF) and diethyl ether (Et_2_O) were distilled from sodium metal and benzophenone. Acetonitrile (MeCN) was distilled from phosphorus pentoxide. Toluene and hexanes were purified by passage through a column of activated alumina. ^1^H and ^13^C­{^1^H} NMR data were recorded on 400 and 500 MHz Bruker Avance NMR spectrometers or Jeol 400 or 500 MHz spectrometers. NMR assignments are made using chemical shifts and multiplicities. A Rigaku Synergy-S diffractometer with dual PhotonJet-S microfocus X-ray sources (Cu Kα, Mo Kα) and a HyPix-6000HE HPC detector was used for crystallographic experiments. UV–vis spectra were recorded on an HP-8452A diode array spectrometer. The ligand FOX^OH^ was synthesized as previously reported.^3^ Cyclic voltammograms were recorded in the drybox using a BASi Epsilon potentiostat equipped with a 3 mm glassy carbon working electrode, a platinum wire counter-electrode, and an Ag/Ag+ reference electrode. Elemental analyses were obtained from the CENTC Elemental Analysis Facility at the University of Rochester. Microanalysis samples were weighed with a PerkinElmer Model AD6000 Autobalance and their compositions were determined with a PerkinElmer 2400 Series II Analyzer.

### Synthesis of FOX^OMe^

In a nitrogen-filled glovebox, a clean dry 50 mL two-necked round-bottom flask was charged with 325 mg (13.5 mmol) of NaH and 5 mL of anhydrous THF. To the stirring solution, a THF solution of 1.01 g (3.39 mmol) of FOX^OH^ ligand was added and the resulting mixture was stirred in an ice bath maintained at 0 °C. After 20 min, 0.9 mL (13.54 mmol) of CH_3_I was added and the reaction mixture was allowed to warm to room temperature and stirred overnight. The reaction was worked up by filtering the excess NaH and washing the solid with hexanes. The solvents and MeI were removed under reduced pressure. The gel-like substance was then dissolved in EtOAc and layered with hexanes to afford FOX^OMe^ as a white crystalline solid (yield: 902 mg, 2.88 mmol, 85%). ^1^H NMR (500 MHz, DMSO-*d*_6_): δ 8.499 (ddd, *J* = 4.7, 1.7, 0.9 Hz, 2H), 7.766 (td, *J* = 7.7, 1.8 Hz, 2H), 7.495 (d, *J* = 7.9 Hz, 2H), 7.302 (ddd, *J* = 7.4, 4.8, 1.1 Hz, 2H), 5.637 (s, 2H), 4.052 (d, *J* = 8.7 Hz, 2H), 3.860 (d, *J* = 8.7 Hz, 2H), 3.393 (s, 2H), 3.243 (s, 3H). ^13^C­{^1^H} NMR (125 MHz, DMSO-*d*_6_): δ 159.00, 148.82, 136.77, 123.36, 120.89, 97.75, 76.34, 73.11, 72.42, 58.89. Anal. Calcd (obsd) for C_17_H_19_N_3_O_3_: 65.16 (64.98) %C, 6.11 (6.34) %H, 13.41 (13.15) %N. IR (ATR, selected peaks): 2866­(m), 1590­(m), 1440­(m), 1110(s), 1075(s), 916(s), 768(s) cm^–1^.

### Synthesis of Mo­(CO)_3_(FOX^OH^), **1**

Mo­(CO)_6_ (176.2 mg, 0.67 mmol) and FOX^OH^ ligand (200 mg, 0.62 mmol) were dissolved in 25 mL of toluene in a round-bottom flask with a septum-capped side arm. The solution was then refluxed under a nitrogen atmosphere for 16 h. The reaction was then cooled to room temperature and the solvent removed under vacuum. An orange solid was obtained (193 mg, 0.40 mmol, 60.3%). ^1^H NMR (500 MHz, CD_3_CN): δ 9.002 (d, *J* = 5.0 Hz, 2H), 7.863 (t, *J* = 7.7 Hz, 2H), 7.507 (d, *J* = 7.6 Hz, 2H), 7.359 (t, *J* = 6.4 Hz, 2H), 5.748 (s, 2H), 4.412 (d, *J* = 9.3 Hz, 2H), 4.232 (d, *J* = 9.3 Hz, 2H), 3.886 (d, *J* = 4.9 Hz, 2H), 3.577 (t, *J* = 4.8 Hz, 1H). ^1^H NMR (500 MHz, DMSO-*d*_6_): δ 8.911 (d, *J* = 4.9 Hz, 2H), 7.953 (t, *J* = 7.2 Hz, 1H), 7.569 (d, *J* = 7.8 Hz, 2H), 7.459 (t, *J* = 6.3 Hz, 2H), 5.945 (s, 2H), 5.518 (s, 1H), 4.339 (d, *J* = 9.2 Hz, 2H), 4.230 (d, *J* = 9.3 Hz, 2H), 3.770 (d, *J* = 5.0 Hz, 2H). ^13^C NMR (126 MHz, CD_3_CN): δ 156.58 (s), 152.43 (s), 139.23 (s), 125.93 (s), 125.06 (s), 100.26 (s), 77.38 (s), 74.16 (s), 68.43 (s). Anal. Calcd (obsd) for MoC_19_H_17_N_3_O_6_: 47.61 (47.42)% C; 3.58 (3.19)% H; 8.77 (7.64)% N. IR­(ATR, selected peaks): 1894 (s) and 1745 (br) cm^–1^.

### Synthesis of Mo­(CO)_3_(FOX^OMe^), **2**

Mo­(CO)_6_ (227.5 mg, 0.86 mmol) and FOX-OMe ligand (270 mg, 0.86 mmol) were dissolved in 20 mL of toluene in a round-bottom flask with a septum-capped side arm. The solution was refluxed under a nitrogen atmosphere for 16 h. The reaction was then cooled to room temperature and the solvents removed under vacuum. An orange solid was obtained (312.8 mg, 0.63 mmol, 73.6%). ^1^H NMR (500 MHz, CD_3_CN): δ 9.001 (d, *J* = 5.3 Hz, 2H), 7.862 (t, *J* = 7.3 Hz, 2H), 7.505 (d, *J* = 7.6 Hz, 2H), 7.356 (t, *J* = 6.4 Hz, 2H), 5.750 (s, 2H), 4.375 (d, *J* = 9.3 Hz, 2H), 4.234 (d, *J* = 9.5 Hz, 2H), 3.788 (s, 2H), 3.356 (s, 3H). ^13^C NMR (126 MHz, CD_3_CN): δ 156.49 (s), 152.36 (s), 139.16 (s), 125.88 (s), 125.06 (s), 99.91 (s), 79.31 (s), 75.82 (s), 74.30 (s), 59.68 (s). Anal. Calcd (obsd) for MoC_20_H_19_N_3_O_6_: 48.69 (48.39)% C; 3.88 (3.86)% H; 8.52 (8.40)% N. IR­(ATR, selected peaks): 1900(s), 1742­(br) cm^–1^.

### Synthesis of MoCl_3_(THF)_3_ ^31^

In a nitrogen filled glovebox, MoCl_5_ (0.5 g, 1.83 mmol) and coarse tin powder (0.43 g, 3.66 mmol) were suspended in 5 mL of Et_2_O in a 20 mL vial. The mixture was then stirred for 30 min at room temperature to form an orange solution and an orange solid. The supernatant liquid was decanted off and 5 mL of THF was added. The mixture was then stirred for 3 h at room temperature. The pale orange-brown crystalline product, MoCl_3_(THF)_3_, was separated from excess tin via mechanical separation. The product was then washed with Et_2_O (2 × 5 mL) and dried under vacuum (0.48 g, 1.15 mmol, 63.0%). Anal. Calcd (obsd) for MoC_12_H_24_Cl_3_O_3_: 34.43 (33.55)% C; 5.78 (5.61)% H; 0 (0.23)% N.

### Synthesis of MoCl_3_(FOX^OMe^), **3**

In a nitrogen filled glovebox, MoCl_3_(THF)_3_ (133.4 mg, 0.32 mmol) and FOX^OH^ (112.4 mg, 0.32 mmol) were added to a Schlenk flask with a stir bar and 20 mL of THF. The vessel was sealed, brought out of the glovebox, and stirred with a condenser attached under nitrogen at 50 °C overnight for 16 h. After the allocated reaction time, the suspension became orange, and upon cooling was brought back into the glovebox. The suspension was filtered on a glass frit, and the solid washed with pentane (3 × 5 mL). The solid was collected and dried in vacuo to obtain the product (132.8 mg, 0.26 mmol, 80.7%). ^1^H NMR (500 MHz, CD_2_Cl_2_): δ 76.27, 35.57, 17.49, 11.17, 8.51, 7.99, −84.41 (all br s). Anal. Calcd (obsd) for MoC_17_H_19_N_3_O_3_Cl_3_: 39.6 (40.21)% C; 3.71 (3.83)% H; 8.15 (7.01)% N.

Crystallographic data (including structure factors) for the structures in this paper have been deposited with the Cambridge Crystallographic Data Center, CCDC, 12 Union Road, Cambridge CB21EZ, UK. Copies of the data can be obtained free of charge on quoting the depository numbers CCDC-2483784 (**1**), CCDC-2483785 (**2**), CCDC-2483786 (**3**), and CCDC-2498084 (FOX^OMe^) (Fax: + 44–1223–336–033; E-mail: deposit@ccdc.cam.ac.uk, http://www.ccdc.cam.ac.uk).

## Supplementary Material





## Notes

Crystallographic data (including structure factors) for the structures (CIF)
^1^H and ^13^C NMR spectra and IR spectra for all compounds; X-ray structure determinations for **1**–**3** and FOX^OMe^ (PDF)
