# Supplementary material for: Synthesis, Characterization, and Electrochemical Studies of Fused Oxazolidine Complexes of Molybdenum
Source: ACS Omega. 2026 Jul 10;11(29):44473–9. doi: 10.1021/acsomega.6c05562 (PMC13425294; doi:10.1021/acsomega.6c05562)
Supplement: Supplementary file 2 [file ao6c05562_si_002.pdf]

Supporting Information

Synthesis, Characterization, and Electrochemical Studies of Fused Oxazolidine Complexes of Molybdenum

Jiayun Li, Aurodeep Panda, William W. Brennessel, William D. Jones*

Department of Chemistry, University of Rochester, Rochester, NY 14627 USA

Table of Contents

Figure S1. ^{1}H NMR spectrum of $Mo(CO)_3(FOX^{OH})$, **1** S-3

Figure S2. $^{13}C\{^{1}H\}$ NMR spectrum of $Mo(CO)_3(FOX^{OH})$, **1** S-4

Figure S3. ATR IR spectrum of $Mo(CO)_3(FOX^{OH})$, **1** S-5

Figure S4. ^{1}H NMR spectrum of FOX^{OMe} in DMSO-d_6 S-6

Figure S5. $^{13}C\{^{1}H\}$ NMR spectrum of FOX^{OMe} in DMSO-d_6 S-7

Figure S6. ATR IR spectrum of FOX^{OMe} S-8

Figure S7. ^{1}H NMR spectrum of $Mo(CO)_3(FOX^{OMe})$, **2** S-9

Figure S8. $^{13}C\{^{1}H\}$ NMR spectrum of $Mo(CO)_3(FOX^{OMe})$, **2** S-10

Figure S9. ATR IR spectrum of $Mo(CO)_3(FOX^{OMe})$, **2** S-11

Figure S10. ^{1}H NMR spectrum (paramagnetic) of $MoCl_3(FOX^{OMe})$, **3** S-12

Figure S11. ATR IR spectrum of $MoCl_3(FOX^{OMe})$, **3** S-13

X-ray structure determination of $Mo(CO)_3(FOX^{OH})$, **1** S-14

X-ray structure determination of $Mo(CO)_3(FOX^{OMe})$, **2** S-39

X-ray structure determination of $MoCl_3(FOX^{OMe})$, **3** S-53

X-ray structure determination of FOX^{OMe} S-66

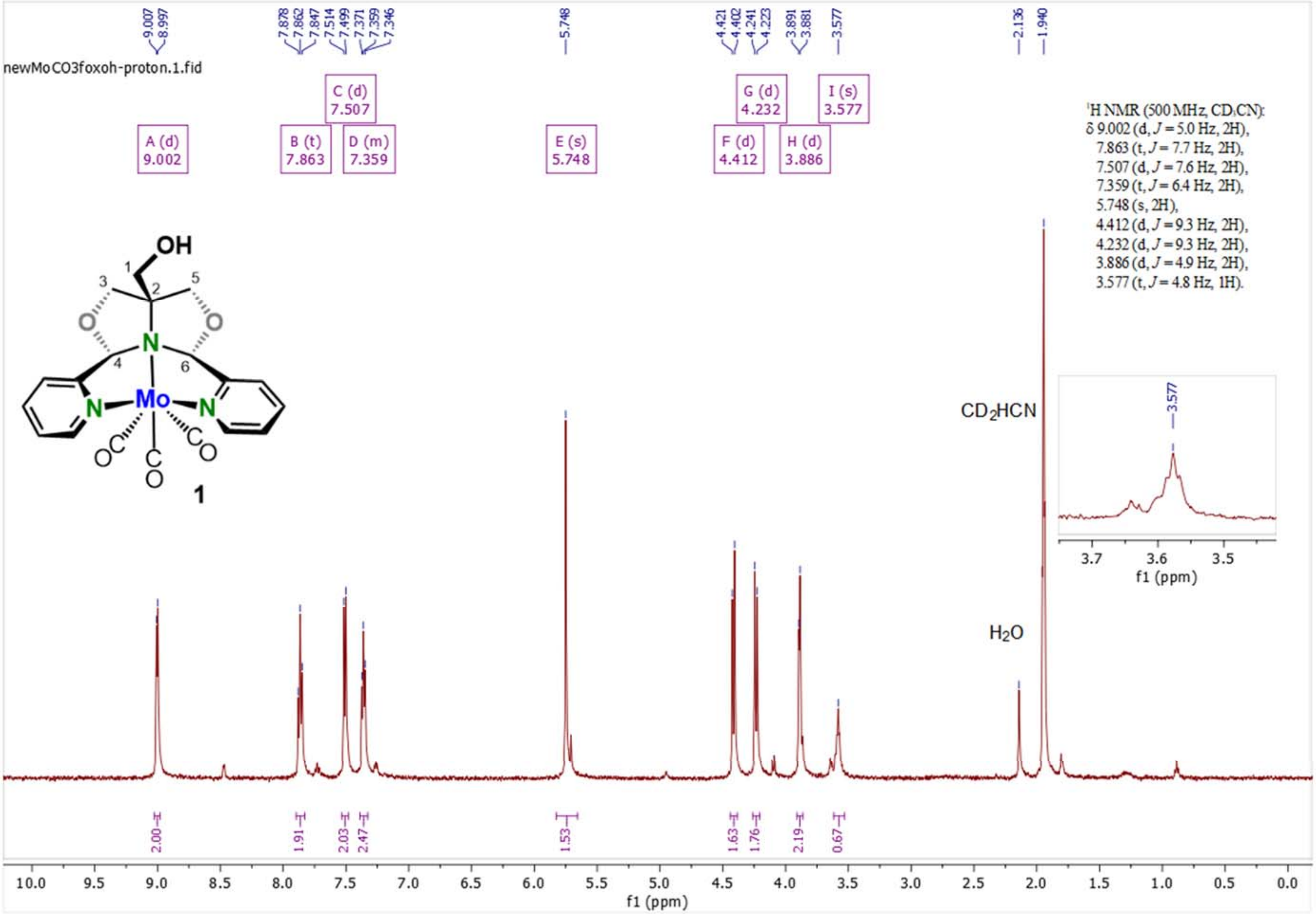

Figure S1. ¹H NMR spectrum of $Mo(CO)_3(FOX^{OH})$, **1**, in CD_3CN.

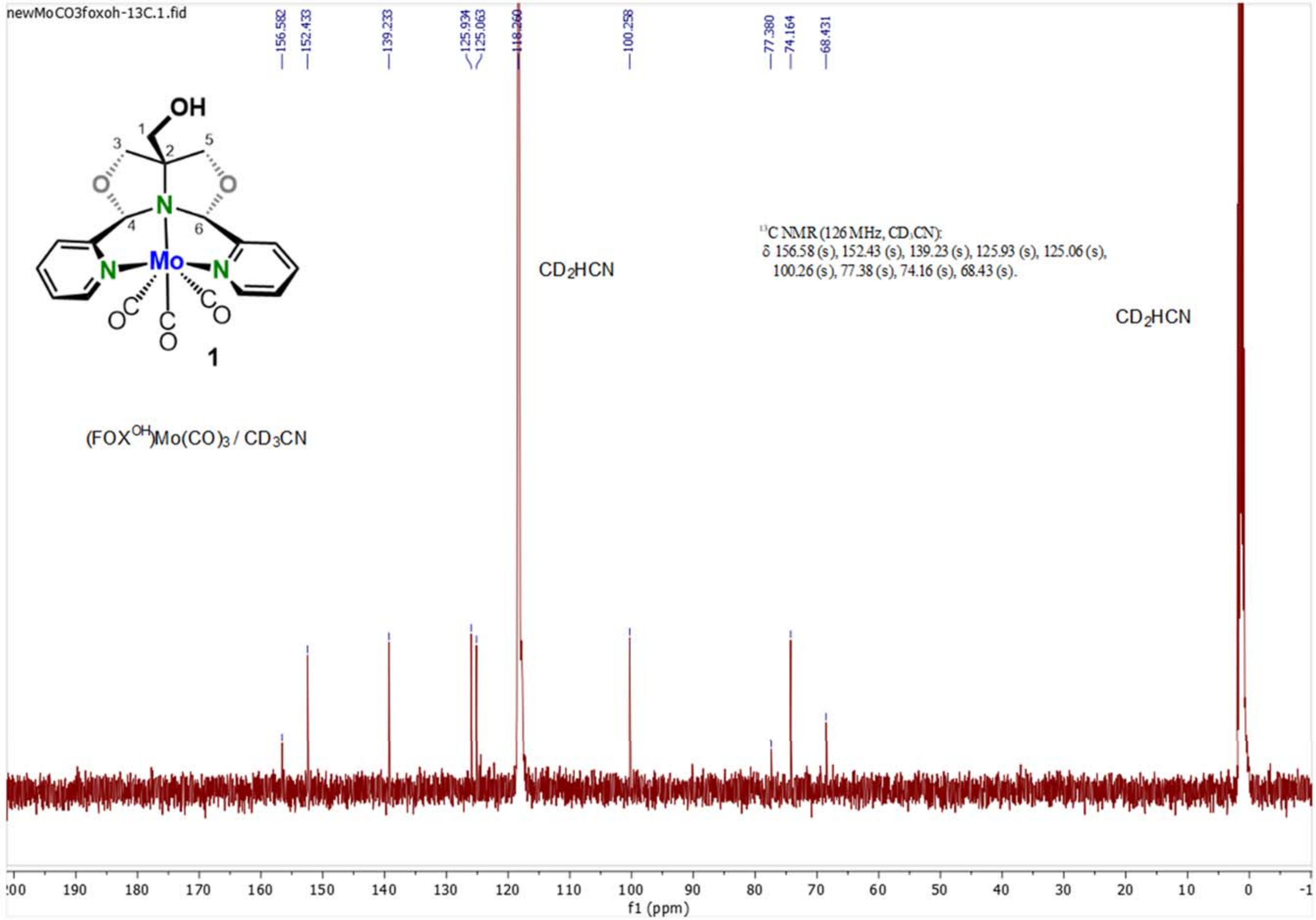

Figure S2. $^{13}C\{^{1}H\}$ NMR spectrum of $Mo(CO)_3(FOX^{OH})$, **1**, in CD_3CN.

PerkinElmer Spectrum IR Version 10.7.
Tuesday, June 11, 2024 5:27 PI

Analyst | Lab
Date | Tuesday, June 11, 2024 5:27 PM

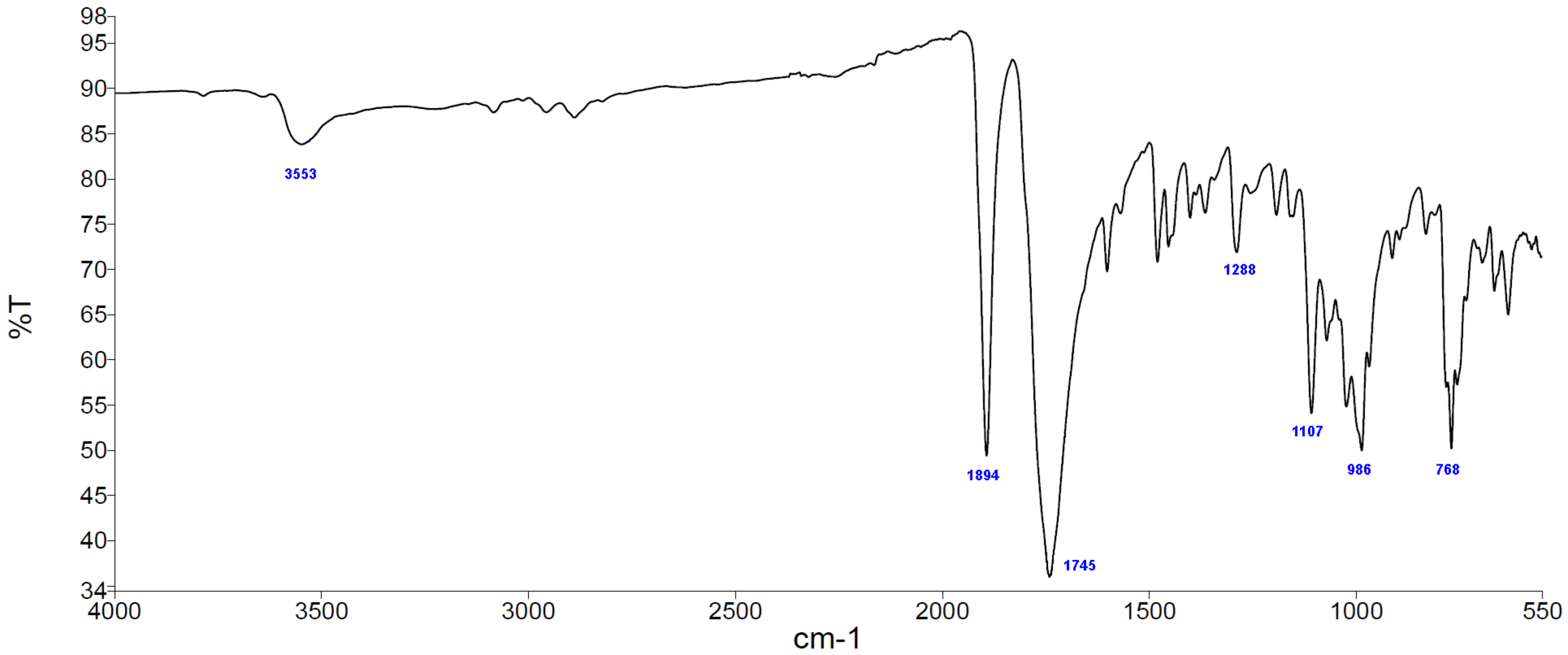

Figure S3. ATR IR spectrum of Mo(CO)3(FOX^OH), **1.**

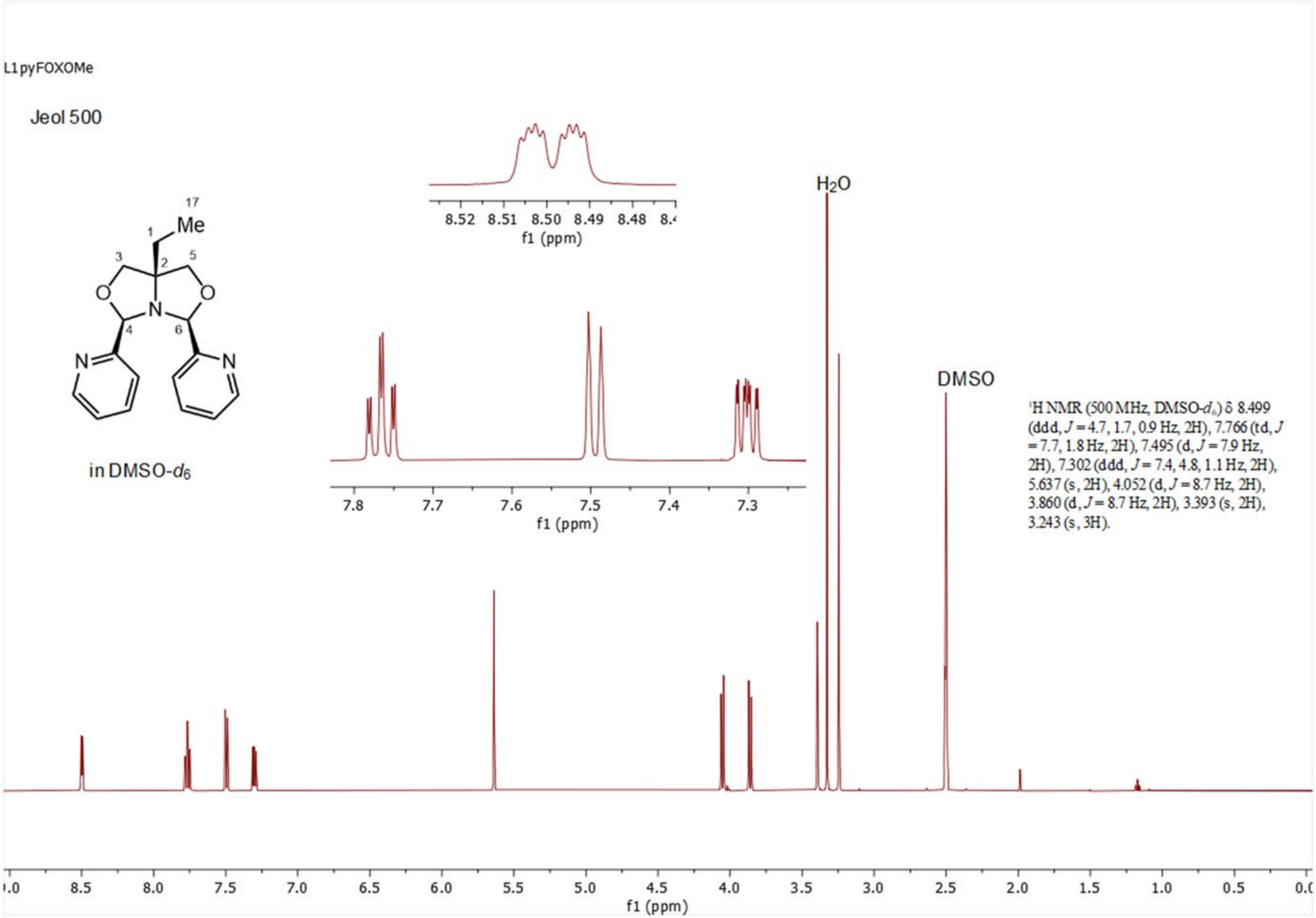

Figure S4. ^{1}H NMR spectrum of FOX^{OMe} in DMSO-d_6.

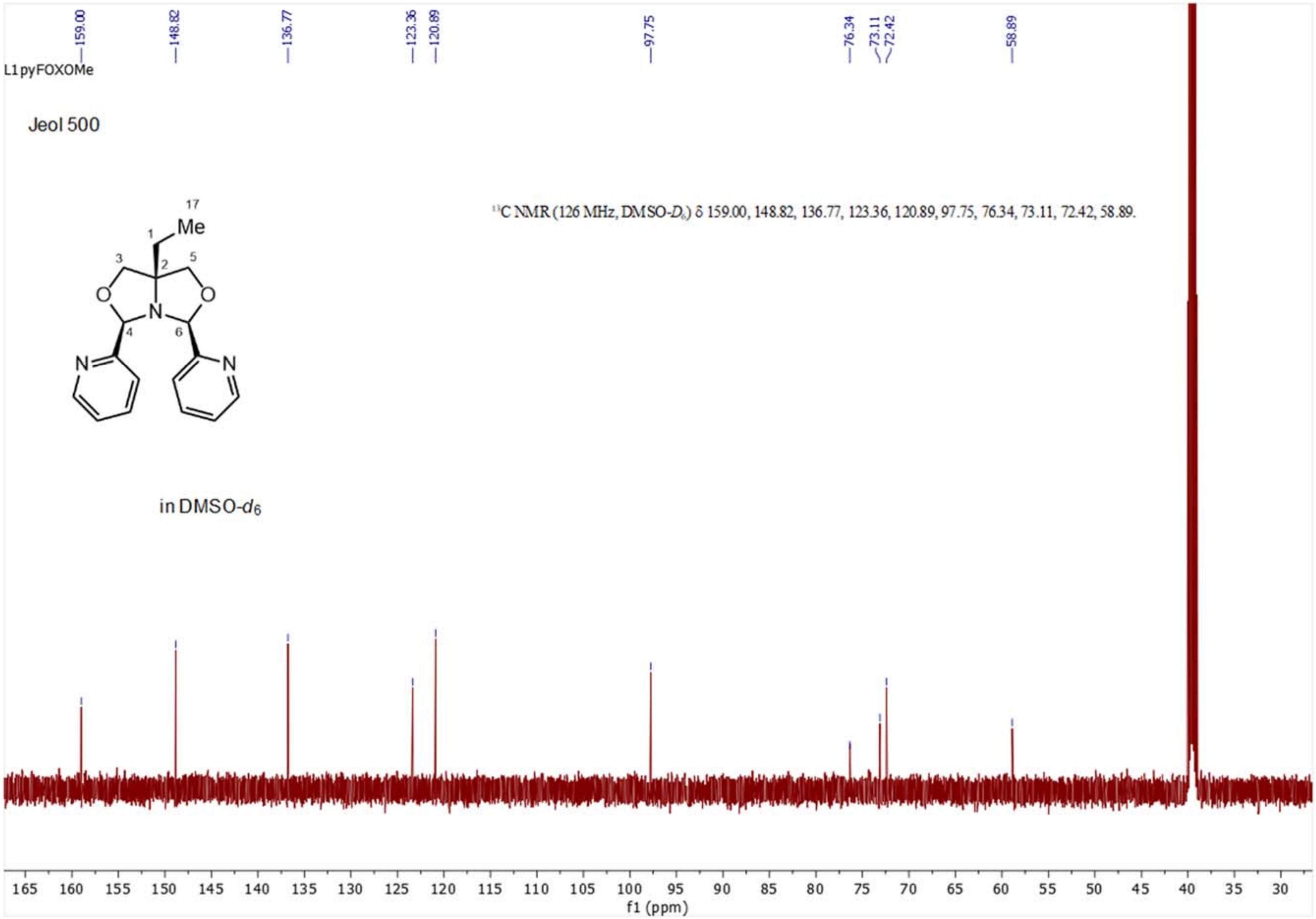

Figure S5. ${}^{13}C\{{}^{1}H\}$ NMR spectrum of FOX^{OMe} in DMSO-d_6.

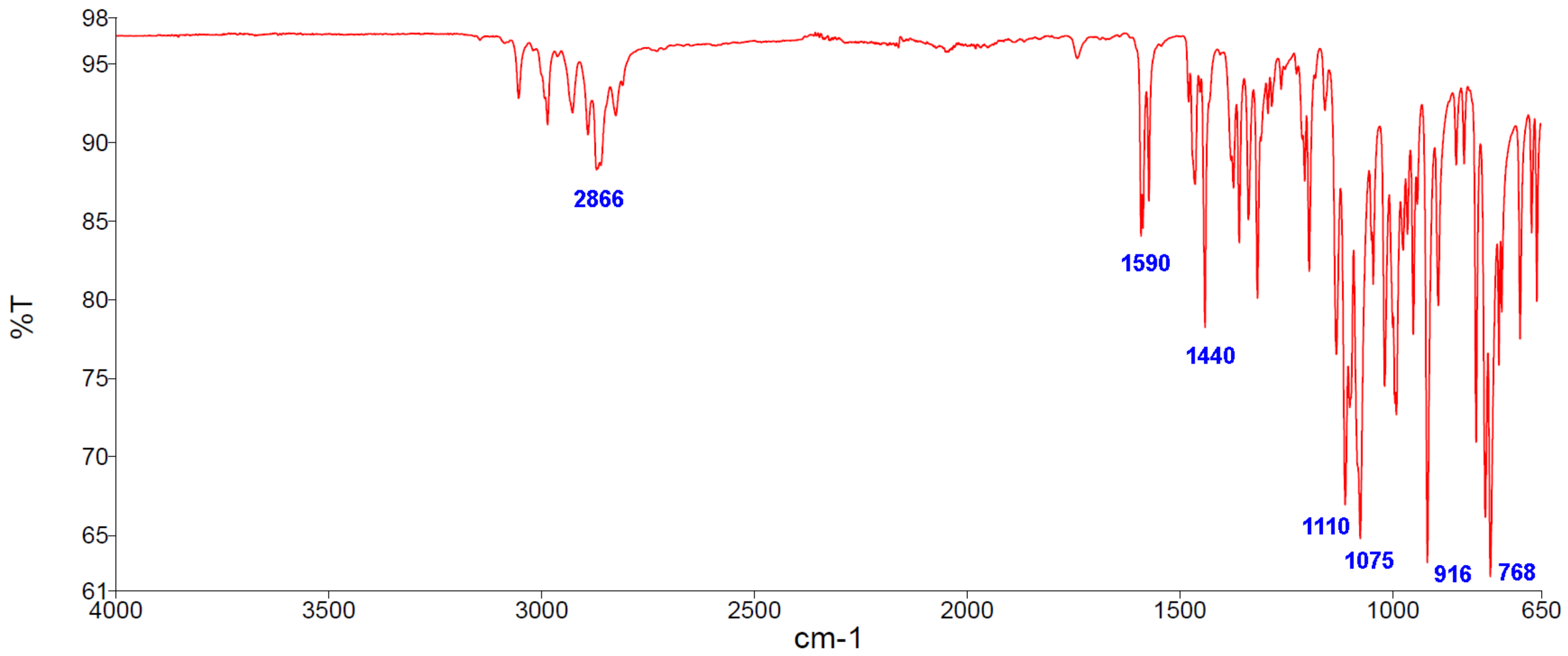

Figure S6. ATR IR spectrum of FOX^{OMe}

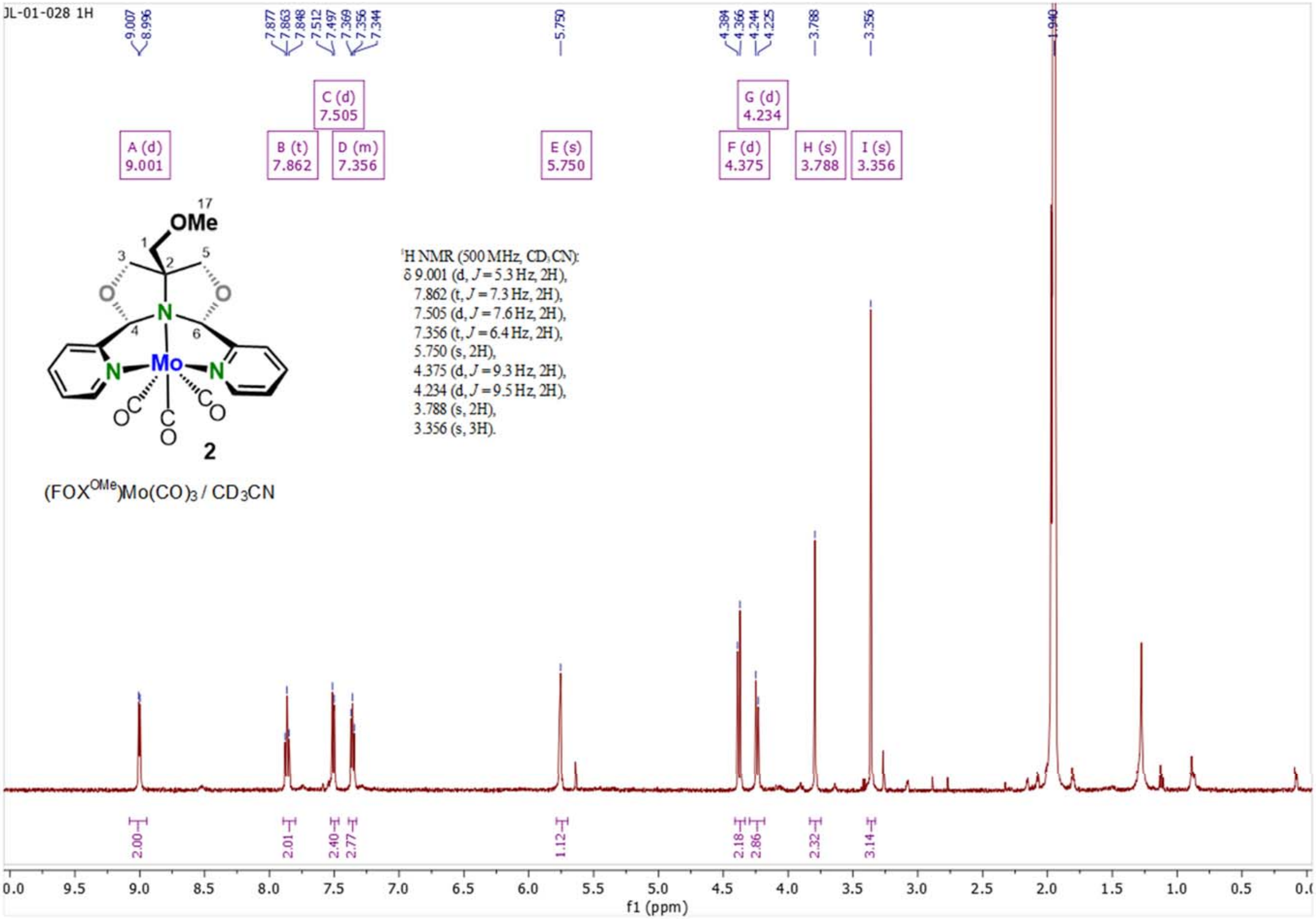

Figure S7. ^{1}H NMR spectrum of $Mo(CO)_3(FOX^{OMe})$, **2**, in CD_3CN.

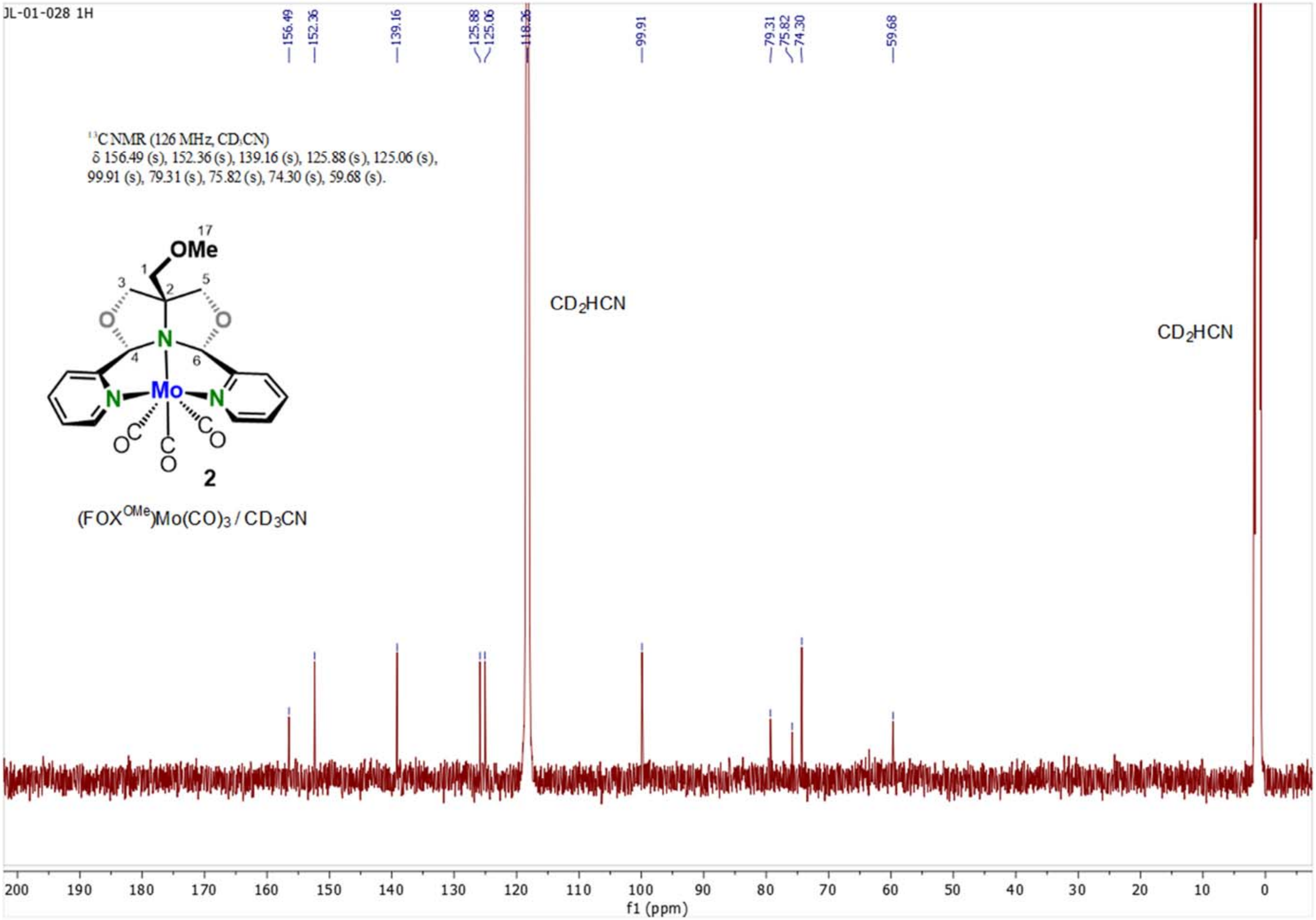

Figure S8. $^{13}C\{^{1}H\}$ NMR spectrum of $Mo(CO)_3(FOX^{OMe})$, **2**, in CD_3CN.

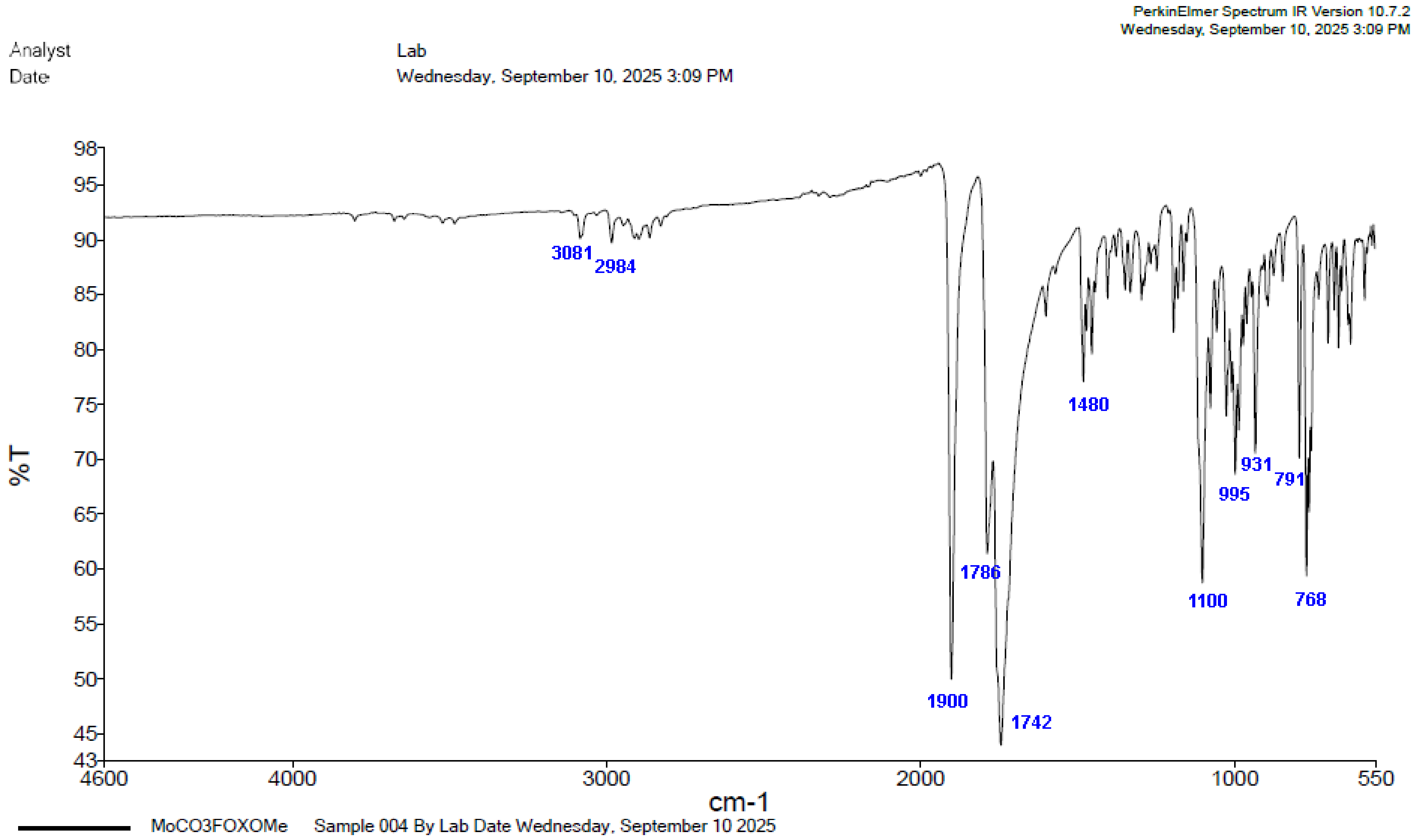

Figure S9. ATR IR spectrum of $Mo(CO)_3(FOX^{OMe})$, **2**.

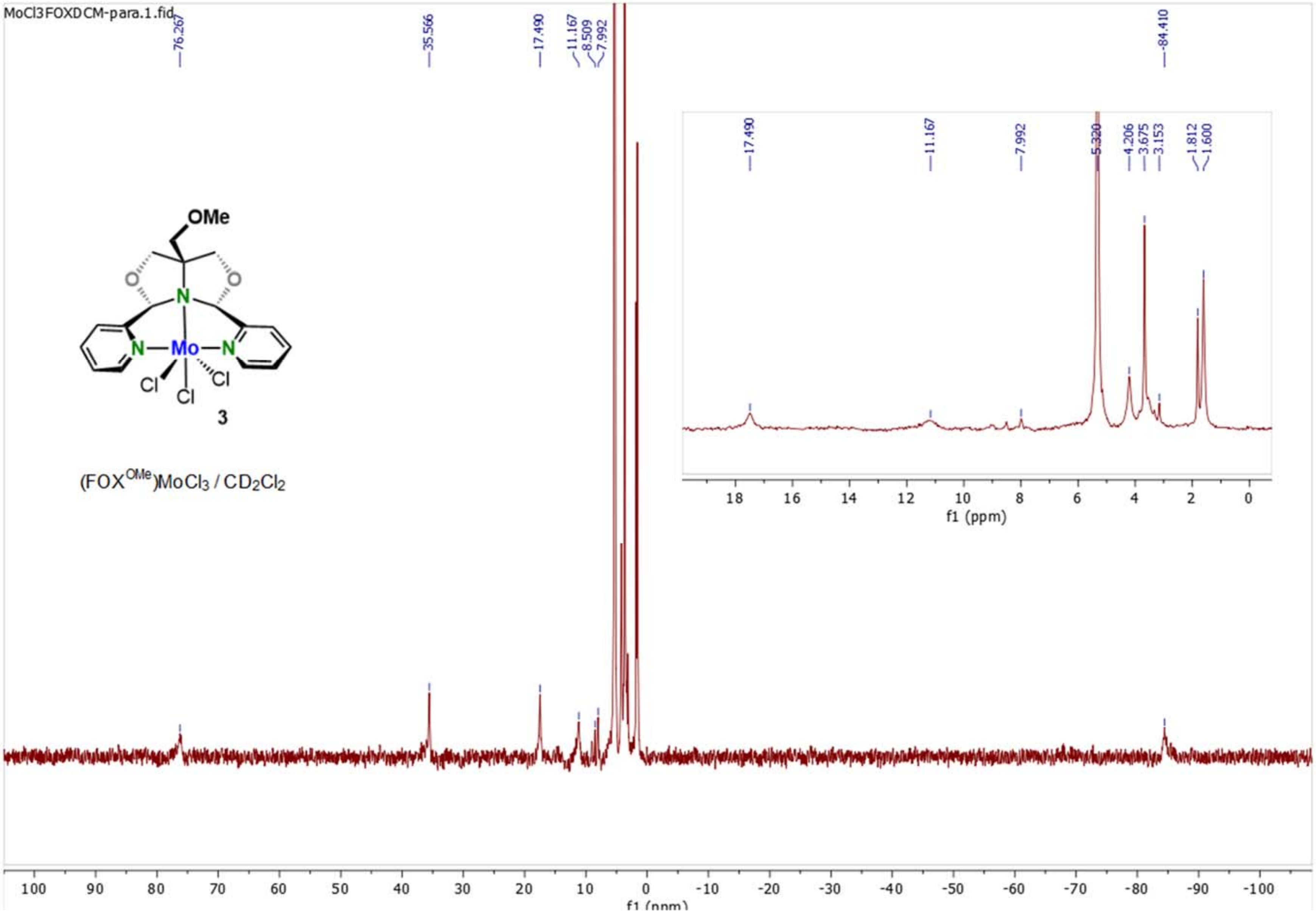

Figure S10. ^{1}H NMR spectrum (paramagnetic) of $MoCl_3(FOX^{OMe})$, **3**.

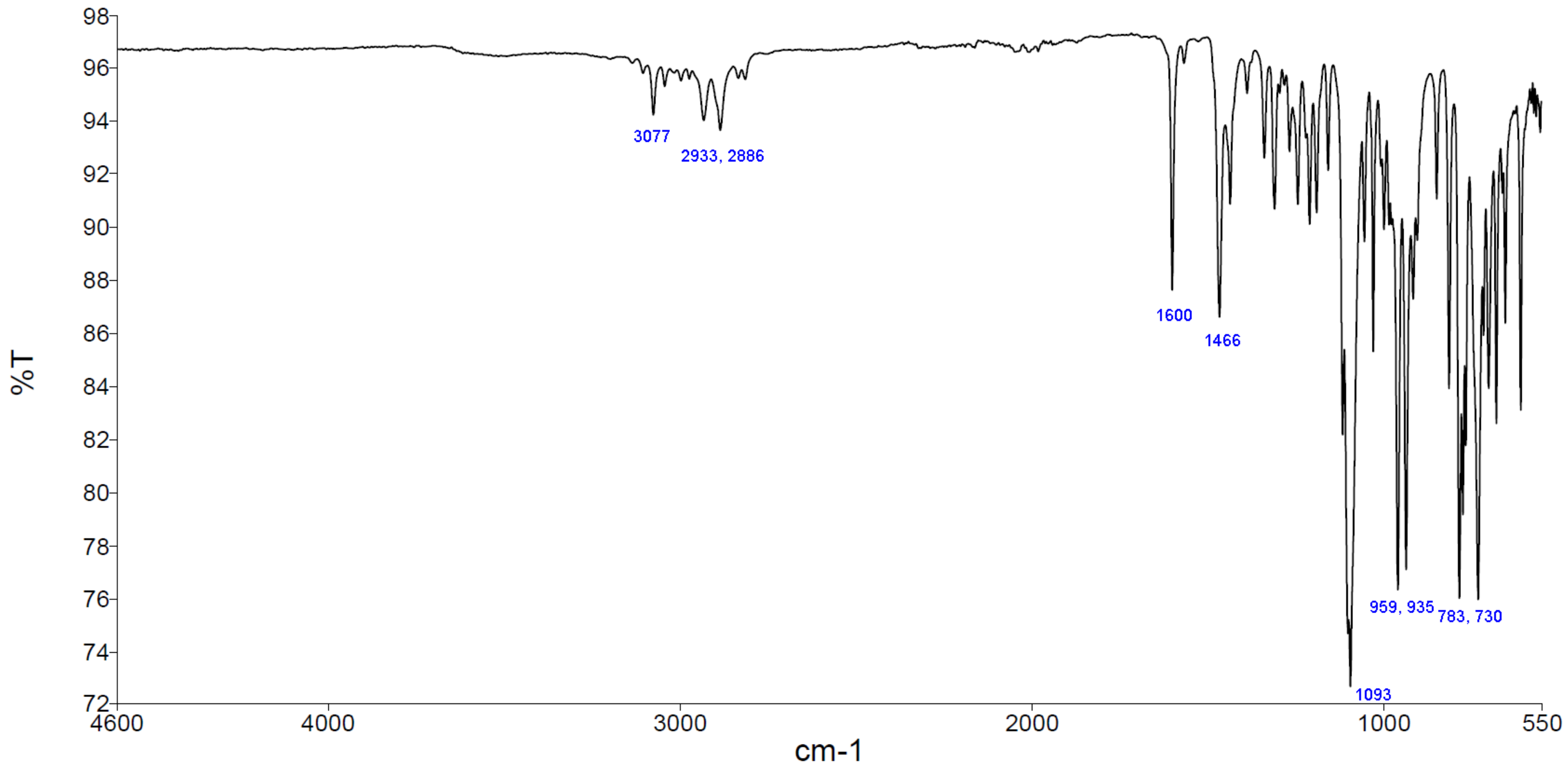

Figure S11. ATR IR spectrum of $MoCl_3(FOX^{OMe})$, **3**.

REFERENCE NUMBER: jonap45

1

CRYSTAL STRUCTURE REPORT

$C_{19}H_{17}MoN_3O_6$

or

(κ^3-FOX^{OH})Mo(CO)$_3$ · xSolvent

Report prepared for:

J. Li, A. Panda, Prof. W. Jones

June 25, 2024

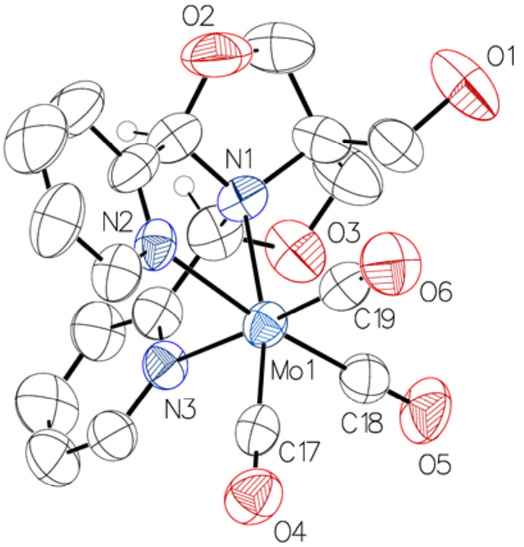

William W. Brennessel

X-ray Crystallographic Facility

Department of Chemistry, University of Rochester

120 Trustee Road

Rochester, NY 14627

Data collection

A crystal (0.283 x 0.102 x 0.015 mm^3) was placed onto a nylon loop and mounted on a Rigaku XtaLAB Synergy-S Dualflex diffractometer equipped with a HyPix-6000HE HPC area detector for data collection at 293 K. A preliminary set of cell constants and an orientation matrix were calculated from a small sampling of reflections.[1] A short pre-experiment was run, from which an optimal data collection strategy was determined. The full data collection was carried out using a PhotonJet (Cu) X-ray source with frame times of 3.53 and 14.13 seconds and a detector distance of 34.0 mm. Series of frames were collected in 0.50º steps in ω at different 2θ, κ, and ϕ settings. After the intensity data were corrected for absorption, the final cell constants were calculated from the xyz centroids of 13458 strong reflections from the actual data collection after integration.[1] See Table S1 for additional crystal and refinement information.

Structure solution and refinement

The structure was solved using SHELXT[2] and refined using SHELXL.[3] The space group $P2_1/c$ was determined based on systematic absences. Most or all non-hydrogen atoms were assigned from the solution. Full-matrix least squares / difference Fourier cycles were performed which located any remaining non-hydrogen atoms. All non-hydrogen atoms were refined with anisotropic displacement parameters. All hydrogen atoms were placed in ideal positions and refined as riding atoms with relative isotropic displacement parameters. Due to the nearby inversion center, a 0.50:0.50 disorder model between donor and acceptor (which is the symmetry equivalent of the donor) is required. When the hydrogen atom is not pointing at the symmetry-equivalent acceptor, the acceptor is likely a solvent molecule (see below).

Reflection contributions from highly disordered solvent were fixed and added to the calculated structure factors using the SQUEEZE routine of program Platon,[4] which determined there to be 181 electrons in 652 Å^3 per unit cell treated this way. Because the exact identity and amount of solvent were unknown, no solvent was included in the atom list or molecular formula. Thus, all calculated quantities that derive from the molecular formula (e.g., F(000), density, molecular weight, etc.) are known to be inaccurate.

The final full matrix least squares refinement converged to $R1 = 0.0738$ (F^2, $I > 2\sigma(I)$) and $wR2 = 0.1948$ (F^2, all data).

Structure description

The structure is the one suggested. The asymmetric unit contains two Mo complexes in general positions and solvent of crystallization whose atoms were not explicitly assigned (see above). A large portion of the multidentate ligand on the molecule containing atom Mo2 is modeled as disordered over two positions (0.51:0.49).

Structure manipulation and figure generation were performed using Olex2.[5] Unless noted otherwise all structural diagrams containing anisotropic displacement ellipsoids are drawn at the 50 % probability level.

Data collection, structure solution, and structure refinement were conducted at the X-ray Crystallographic Facility, B04 Hutchison Hall, Department of Chemistry, University of Rochester. The instrument was purchased with funding from NSF MRI program grant CHE-1725028. All publications arising from this report MUST either 1) include William W. Brennessel

as a coauthor or 2) acknowledge William W. Brennessel and the X-ray Crystallographic Facility of the Department of Chemistry at the University of Rochester.

---

[1] *CrysAlisPro*, version 171.42.90a; Rigaku Corporation: Oxford, UK, 2023.

[2] Sheldrick, G. M. *SHELXT* – Integrated space-group and crystal-structure determination. *Acta. Crystallogr.* **2015**, *A71*, 3-8. *SHELXT*, version 2018/2

[3] Sheldrick, G. M. Crystal structure refinement with *SHELXL*. *Acta. Crystallogr.* 2015, *C71*, 3-8. *SHELXL*, version 2019/2

[4] Spek, A. L. *PLATON* SQUEEZE: a tool for the calculation of the disorder solvent contribution to the calculated structure factors. *Acta. Crystallogr.* **2015**, *C71*, 9-18. *PLATON*, version 250420

[5] Dolomanov, O. V.; Bourhis, L. J.; Gildea, R. J.; Howard, J. A. K.; Puschmann, H. *OLEX2*: a complete structure solution, refinement and analysis program. *J. Appl. Cryst.* **2009**, *42*, 339-341. *Olex2*, version 1.5

Some equations of interest:

$$R_{\mathrm{int}} = \Sigma\,|F_o^2 - \langle F_o^2\rangle| \,/\, \Sigma|F_o^2|$$
$$R1 = \Sigma||F_o|-|F_c|| \,/\, \Sigma|F_o|$$
$$wR2 = [\Sigma[w(F_o^2-F_c^2)^2] \,/\, \Sigma\,[w(F_o^2)^2]]^{1/2}$$
$$\text{where } w = 1 / [\sigma^2(F_o^2) + (aP)^2 + bP] \text{ and}$$
$$P = 1/3 \max(0, F_o^2) + 2/3\, F_c^2$$
$$\mathrm{GOF} = S = [\Sigma[w(F_o^2-F_c^2)^2] \,/\, (m-n)]^{1/2}$$

where m = number of reflections and n = number of parameters

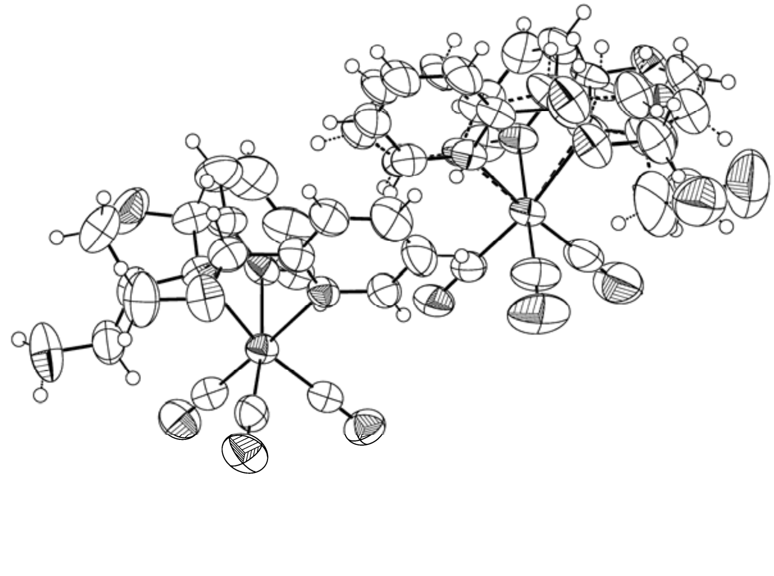

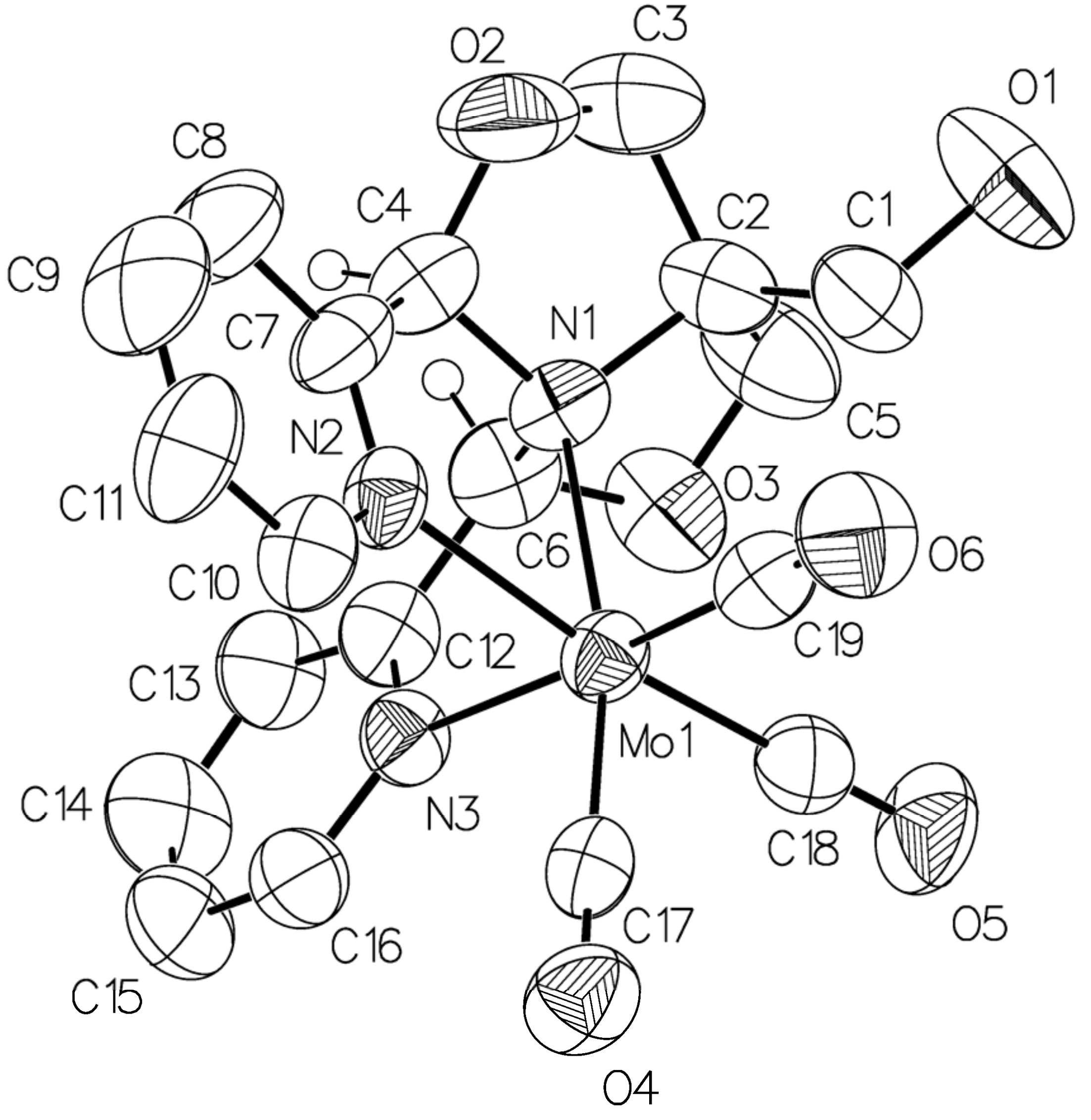
O2
C3
O1
C8
C4
C2
C1
C9
C7
N1
N2
C5
C11
O3
C6
O6
C10
C19
C12
C13
Mo1
C14
N3
C18
C17
C16
O5
C15
O4

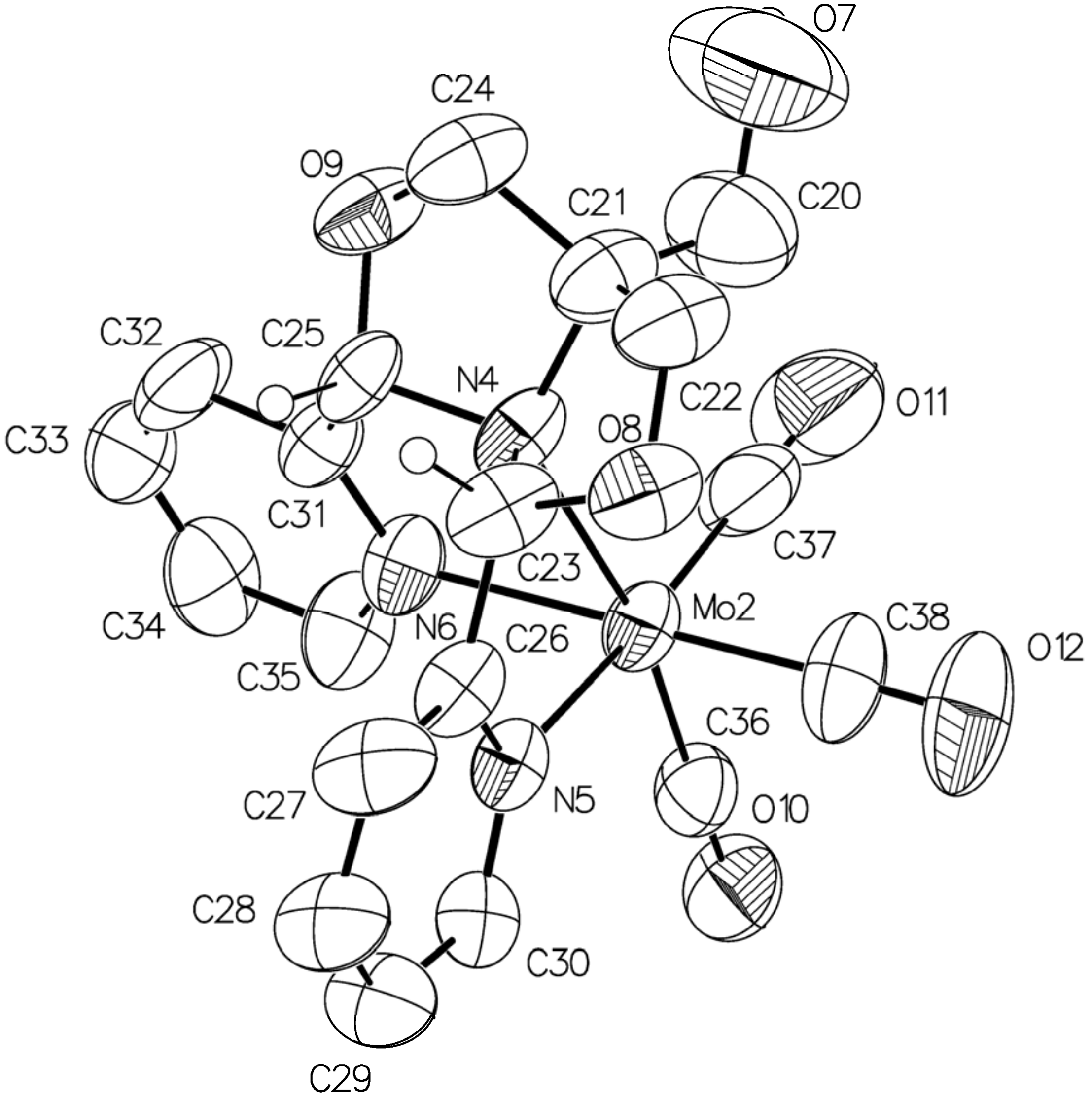
O7
C24
O9
C21
C20
C32
C25
N4
C22
O11
C33
O8
C31
C37
C23
Mo2
C34
N6
C26
C38
O12
C35
C36
C27
N5
O10
C28
C30
C29

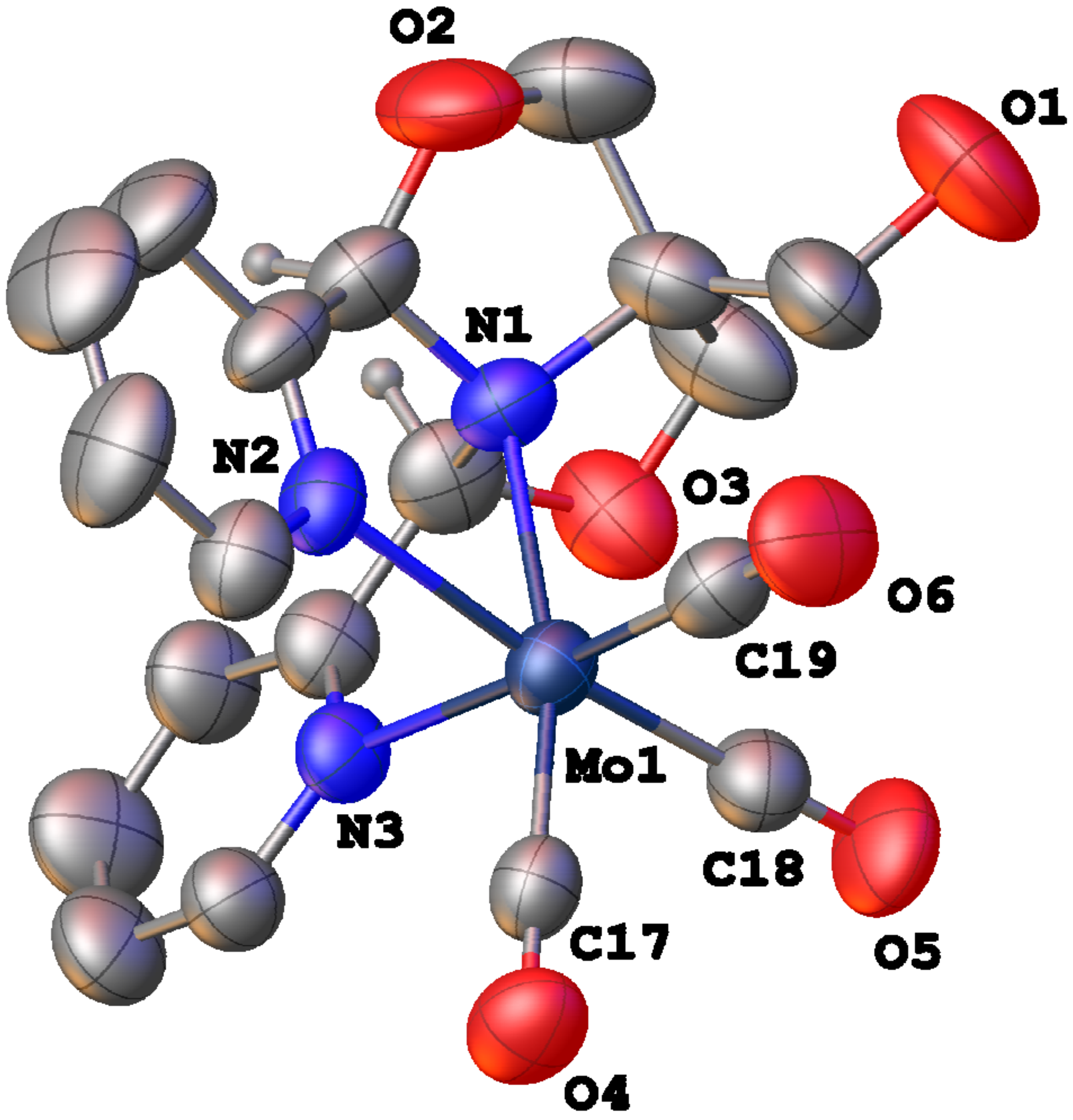
O2
O1
N1
N2
O3
O6
C19
Mo1
N3
C18
C17
O5
O4

Table S1. Crystal data and structure refinement for jonap45.

Identification code	jonap45	
Empirical formula	C19 H17 Mo N3 O6	
Formula weight	479.29	
Temperature	293 K	
Wavelength	1.54184 Å	
Crystal system	monoclinic	
Space group	$P2_1/c$	
Unit cell dimensions	a = 30.6679(4) Å	α = 90°
	b = 9.04440(10) Å	β = 93.2480(10)°
	c = 15.7245(2) Å	γ = 90°
Volume	4354.54(9) $Å^3$	
Z	8	
Density (calculated)	1.462 Mg/m^3	
Absorption coefficient	5.266 mm^{-1}	
F(000)	1936	
Crystal color, morphology	yellow, plate	
Crystal size	0.283 x 0.102 x 0.015 mm^3	
Theta range for data collection	2.886 to 77.971°	
Index ranges	$-38 \leq h \leq 37$, $-5 \leq k \leq 11$, $-19 \leq l \leq 19$	
Reflections collected	33960	
Independent reflections	8879 [R(int) = 0.0426]	
Observed reflections	6959	
Completeness to theta = 67.684°	100.0%	
Absorption correction	Multi-scan	
Max. and min. transmission	1.00000 and 0.85451	
Refinement method	Full-matrix least-squares on F^2	
Data / restraints / parameters	8879 / 348 / 670	
Goodness-of-fit on F^2	1.074	
Final R indices [I>2sigma(I)]	$R1$ = 0.0738, $wR2$ = 0.1875	
R indices (all data)	$R1$ = 0.0879, $wR2$ = 0.1948	
Largest diff. peak and hole	1.249 and -1.142 e.$Å^{-3}$	

Table S2. Atomic coordinates (x 10^4) and equivalent isotropic displacement parameters ($Å^2$x 10^3) for jonap45. U_{eq} is defined as one third of the trace of the orthogonalized U_{ij} tensor.

	x	y	z	U_{eq}
Mo1	8172(1)	5048(1)	4418(1)	48(1)
O1	9742(2)	5666(10)	4382(6)	119(3)
O2	9091(2)	6434(10)	6327(4)	99(2)
O3	8667(2)	8558(7)	4060(4)	78(2)
O4	7416(2)	3108(7)	3709(4)	76(2)
O5	8374(2)	5573(8)	2544(4)	88(2)
O6	8706(2)	2164(7)	4377(5)	88(2)
N1	8640(2)	6831(6)	5110(4)	53(1)
N2	8115(2)	4744(6)	5829(3)	51(1)
N3	7769(2)	7114(6)	4544(4)	51(1)
C1	9286(3)	5506(12)	4535(7)	83(3)
C2	9122(3)	6930(10)	4885(6)	69(2)
C3	9341(3)	7259(13)	5766(7)	97(3)
C4	8649(3)	6685(10)	6053(5)	68(2)
C5	9114(3)	8199(12)	4235(8)	95(3)
C6	8473(3)	8321(8)	4831(6)	65(2)
C7	8363(3)	5473(9)	6376(4)	62(2)
C8	8386(4)	5221(13)	7256(6)	93(3)
C9	8116(4)	4124(15)	7542(7)	106(4)
C10	7849(4)	3378(11)	6974(6)	90(3)
C11	7849(3)	3695(9)	6122(5)	65(2)
C12	7985(3)	8390(8)	4723(5)	58(2)
C13	7774(3)	9741(9)	4816(6)	72(2)
C14	7329(4)	9761(10)	4707(7)	89(3)
C15	7106(3)	8473(11)	4544(6)	80(3)
C16	7335(3)	7171(9)	4469(5)	62(2)
C17	7706(2)	3845(8)	3988(4)	52(2)
C18	8296(3)	5407(9)	3247(5)	62(2)
C19	8513(3)	3258(9)	4381(5)	60(2)
Mo2	6193(1)	5174(1)	6300(1)	50(1)
O10	6833(2)	2747(7)	5826(4)	85(2)
O11	5503(3)	2808(11)	5880(6)	141(4)
O12	6195(3)	5859(10)	4382(4)	126(3)

N6	6198(2)	4649(6)	7693(4)	55(1)
C31	5929(2)	5386(8)	8178(4)	52(2)
C32	5871(3)	5049(11)	9017(5)	77(3)
C33	6092(3)	3883(13)	9361(6)	84(3)
C34	6369(3)	3098(11)	8890(6)	80(3)
C35	6415(3)	3523(9)	8055(5)	73(2)
C36	6595(3)	3676(9)	6013(5)	58(2)
C37	5764(3)	3706(13)	6048(6)	89(3)
C38	6186(4)	5652(10)	5105(5)	80(3)
O7	4633(6)	6200(30)	6150(20)	199(9)
O8	5815(4)	8907(13)	5975(8)	71(3)
O9	5276(4)	6657(15)	8014(8)	72(3)
N5	6641(5)	7056(15)	6625(15)	46(3)
C21	5283(6)	7350(20)	6615(11)	71(3)
C22	5351(5)	8760(20)	6039(12)	80(3)
C23	5965(5)	8480(20)	6767(12)	64(3)
C24	5066(6)	7670(20)	7432(13)	77(4)
C25	5720(5)	6817(18)	7871(13)	51(3)
C26	6468(5)	8351(17)	6819(13)	53(3)
C27	6728(7)	9550(20)	7040(20)	79(5)
C28	7168(7)	9390(20)	7120(20)	79(4)
C29	7348(6)	8060(20)	6940(20)	78(4)
C30	7073(6)	6952(19)	6643(16)	52(3)
N4	5759(7)	7009(16)	6933(14)	54(2)
C20	5075(7)	6000(30)	6148(19)	114(6)
O7'	5062(8)	8380(20)	5137(13)	145(8)
O8'	5976(4)	9593(13)	6504(10)	78(3)
O9'	5213(4)	6201(14)	7505(10)	70(3)
N5'	6701(5)	6826(15)	6760(15)	46(3)
C21'	5405(6)	7840(20)	6452(10)	70(3)
C22'	5510(6)	9560(20)	6522(16)	85(5)
C23'	6097(5)	8467(19)	7054(12)	62(4)
C24'	5042(6)	7370(20)	6972(15)	80(4)
C25'	5647(5)	6570(20)	7721(12)	51(4)
C26'	6593(5)	8183(17)	6999(13)	49(3)
C27'	6883(6)	9312(19)	7210(20)	76(4)
C28'	7313(6)	9020(20)	7140(19)	74(4)
C29'	7438(6)	7650(20)	6920(20)	75(5)

C30'	7127(6)	6566(18)	6784(17)	54(4)
N4'	5800(7)	7156(15)	6900(13)	51(3)
C20'	5343(11)	7350(40)	5513(17)	181(18)

Table S3. Bond lengths [Å] and angles [°] for jonap45.

Mo(1)-N(1)	2.380(6)	C(7)-C(8)	1.400(11)
Mo(1)-N(2)	2.252(5)	C(8)-H(8)	0.9300
Mo(1)-N(3)	2.255(5)	C(8)-C(9)	1.382(16)
Mo(1)-C(17)	1.892(8)	C(9)-H(9)	0.9300
Mo(1)-C(18)	1.929(8)	C(9)-C(10)	1.357(15)
Mo(1)-C(19)	1.929(8)	C(10)-H(10)	0.9300
O(1)-H(1A)	0.9601	C(10)-C(11)	1.371(11)
O(1)-H(1B)	0.9602	C(11)-H(11)	0.9300
O(1)-C(1)	1.440(10)	C(12)-C(13)	1.395(10)
O(2)-C(3)	1.413(12)	C(13)-H(13)	0.9300
O(2)-C(4)	1.418(10)	C(13)-C(14)	1.365(13)
O(3)-C(5)	1.422(11)	C(14)-H(14)	0.9300
O(3)-C(6)	1.396(10)	C(14)-C(15)	1.368(14)
O(4)-C(17)	1.175(9)	C(15)-H(15)	0.9300
O(5)-C(18)	1.154(9)	C(15)-C(16)	1.380(11)
O(6)-C(19)	1.153(9)	C(16)-H(16)	0.9300
N(1)-C(2)	1.544(10)	Mo(2)-N(6)	2.241(6)
N(1)-C(4)	1.487(9)	Mo(2)-C(36)	1.903(8)
N(1)-C(6)	1.498(9)	Mo(2)-C(37)	1.894(11)
N(2)-C(7)	1.297(10)	Mo(2)-C(38)	1.928(8)
N(2)-C(11)	1.348(10)	Mo(2)-N(5)	2.230(11)
N(3)-C(12)	1.352(10)	Mo(2)-N(4)	2.381(12)
N(3)-C(16)	1.331(9)	Mo(2)-N(5')	2.248(11)
C(1)-H(1C)	0.9700	Mo(2)-N(4')	2.383(12)
C(1)-H(1D)	0.9700	O(10)-C(36)	1.162(9)
C(1)-C(2)	1.498(13)	O(11)-C(37)	1.161(12)
C(2)-C(3)	1.534(13)	O(12)-C(38)	1.153(10)
C(2)-C(5)	1.536(12)	N(6)-C(31)	1.333(9)
C(3)-H(3A)	0.9700	N(6)-C(35)	1.327(9)
C(3)-H(3B)	0.9700	C(31)-C(32)	1.375(10)
C(4)-H(4)	0.9800	C(31)-C(25)	1.510(14)
C(4)-C(7)	1.508(12)	C(31)-C(25')	1.529(14)
C(5)-H(5A)	0.9700	C(32)-H(32)	0.9300
C(5)-H(5B)	0.9700	C(32)-C(33)	1.350(13)
C(6)-H(6)	0.9800	C(33)-H(33)	0.9300
C(6)-C(12)	1.498(11)	C(33)-C(34)	1.360(14)

C(34)-H(34)	0.9300	O(8')-C(22')	1.432(18)
C(34)-C(35)	1.383(11)	O(8')-C(23')	1.373(16)
C(35)-H(35)	0.9300	O(9')-C(24')	1.430(16)
O(7)-H(7)	0.8200	O(9')-C(25')	1.393(15)
O(7)-C(20)	1.37(2)	N(5')-C(26')	1.332(13)
O(8)-C(22)	1.440(18)	N(5')-C(30')	1.326(14)
O(8)-C(23)	1.358(17)	C(21')-C(22')	1.59(2)
O(9)-C(24)	1.421(16)	C(21')-C(24')	1.48(2)
O(9)-C(25)	1.400(16)	C(21')-N(4')	1.50(3)
N(5)-C(26)	1.328(14)	C(21')-C(20')	1.54(3)
N(5)-C(30)	1.326(15)	C(22')-H(22C)	0.9700
C(21)-C(22)	1.59(2)	C(22')-H(22D)	0.9700
C(21)-C(24)	1.51(2)	C(23')-H(23')	0.9800
C(21)-N(4)	1.55(3)	C(23')-C(26')	1.549(17)
C(21)-C(20)	1.54(2)	C(23')-N(4')	1.506(14)
C(22)-H(22A)	0.9700	C(24')-H(24C)	0.9700
C(22)-H(22B)	0.9700	C(24')-H(24D)	0.9700
C(23)-H(23)	0.9800	C(25')-H(25')	0.9800
C(23)-C(26)	1.545(17)	C(25')-N(4')	1.496(13)
C(23)-N(4)	1.506(14)	C(26')-C(27')	1.383(17)
C(24)-H(24A)	0.9700	C(27')-H(27')	0.9300
C(24)-H(24B)	0.9700	C(27')-C(28')	1.354(19)
C(25)-H(25)	0.9800	C(28')-H(28')	0.9300
C(25)-N(4)	1.497(14)	C(28')-C(29')	1.355(18)
C(26)-C(27)	1.379(17)	C(29')-H(29')	0.9300
C(27)-H(27)	0.9300	C(29')-C(30')	1.373(17)
C(27)-C(28)	1.36(2)	C(30')-H(30')	0.9300
C(28)-H(28)	0.9300	C(20')-H(20C)	0.9700
C(28)-C(29)	1.358(19)	C(20')-H(20D)	0.9700
C(29)-H(29)	0.9300	N(2)-Mo(1)-N(1)	73.2(2)
C(29)-C(30)	1.375(17)	N(2)-Mo(1)-N(3)	86.6(2)
C(30)-H(30)	0.9300	N(3)-Mo(1)-N(1)	73.6(2)
C(20)-H(20B)	0.9700	C(17)-Mo(1)-N(1)	167.7(3)
C(20)-H(20A)	0.9700	C(17)-Mo(1)-N(2)	100.5(3)
O(7')-H(7')	0.8200	C(17)-Mo(1)-N(3)	95.8(3)
O(7')-C(20')	1.38(2)	C(17)-Mo(1)-C(18)	86.7(3)

C(17)-Mo(1)-C(19)	84.6(3)	O(2)-C(3)-C(2)	103.6(7)
C(18)-Mo(1)-C(19)	88.6(3)	O(2)-C(3)-H(3A)	111.0
C(18)-Mo(1)-N(1)	100.2(3)	O(2)-C(3)-H(3B)	111.0
C(18)-Mo(1)-N(2)	172.5(3)	C(2)-C(3)-H(3A)	111.0
C(18)-Mo(1)-N(3)	94.8(3)	C(2)-C(3)-H(3B)	111.0
C(19)-Mo(1)-N(1)	105.6(3)	H(3A)-C(3)-H(3B)	109.0
C(19)-Mo(1)-N(2)	90.0(3)	O(2)-C(4)-N(1)	106.2(7)
C(19)-Mo(1)-N(3)	176.6(3)	O(2)-C(4)-H(4)	108.4
C(1)-O(1)-H(1A)	109.3	O(2)-C(4)-C(7)	110.2(7)
C(1)-O(1)-H(1B)	109.2	N(1)-C(4)-H(4)	108.4
C(3)-O(2)-C(4)	105.7(7)	N(1)-C(4)-C(7)	114.9(6)
C(6)-O(3)-C(5)	104.5(7)	C(7)-C(4)-H(4)	108.4
C(2)-N(1)-Mo(1)	119.6(5)	O(3)-C(5)-C(2)	106.2(7)
C(4)-N(1)-Mo(1)	111.9(4)	O(3)-C(5)-H(5A)	110.5
C(4)-N(1)-C(2)	105.7(6)	O(3)-C(5)-H(5B)	110.5
C(4)-N(1)-C(6)	111.1(6)	C(2)-C(5)-H(5A)	110.5
C(6)-N(1)-Mo(1)	106.8(4)	C(2)-C(5)-H(5B)	110.5
C(6)-N(1)-C(2)	101.3(6)	H(5A)-C(5)-H(5B)	108.7
C(7)-N(2)-Mo(1)	121.1(5)	O(3)-C(6)-N(1)	103.8(6)
C(7)-N(2)-C(11)	118.3(7)	O(3)-C(6)-H(6)	109.4
C(11)-N(2)-Mo(1)	120.3(5)	O(3)-C(6)-C(12)	111.5(7)
C(12)-N(3)-Mo(1)	117.4(5)	N(1)-C(6)-H(6)	109.4
C(16)-N(3)-Mo(1)	125.1(5)	N(1)-C(6)-C(12)	113.1(6)
C(16)-N(3)-C(12)	117.5(6)	C(12)-C(6)-H(6)	109.4
O(1)-C(1)-H(1C)	109.9	N(2)-C(7)-C(4)	118.5(6)
O(1)-C(1)-H(1D)	109.9	N(2)-C(7)-C(8)	124.5(9)
O(1)-C(1)-C(2)	108.9(9)	C(8)-C(7)-C(4)	117.0(8)
H(1C)-C(1)-H(1D)	108.3	C(7)-C(8)-H(8)	121.9
C(2)-C(1)-H(1C)	109.9	C(9)-C(8)-C(7)	116.3(10)
C(2)-C(1)-H(1D)	109.9	C(9)-C(8)-H(8)	121.9
C(1)-C(2)-N(1)	112.3(7)	C(8)-C(9)-H(9)	120.3
C(1)-C(2)-C(3)	111.1(8)	C(10)-C(9)-C(8)	119.5(9)
C(1)-C(2)-C(5)	113.1(9)	C(10)-C(9)-H(9)	120.3
C(3)-C(2)-N(1)	100.4(7)	C(9)-C(10)-H(10)	119.8
C(3)-C(2)-C(5)	116.4(9)	C(9)-C(10)-C(11)	120.4(10)
C(5)-C(2)-N(1)	102.5(6)	C(11)-C(10)-H(10)	119.8

N(2)-C(11)-C(10)	121.0(9)
N(2)-C(11)-H(11)	119.5
C(10)-C(11)-H(11)	119.5
N(3)-C(12)-C(6)	117.6(6)
N(3)-C(12)-C(13)	123.0(8)
C(13)-C(12)-C(6)	119.5(7)
C(12)-C(13)-H(13)	121.1
C(14)-C(13)-C(12)	117.7(8)
C(14)-C(13)-H(13)	121.1
C(13)-C(14)-H(14)	120.1
C(13)-C(14)-C(15)	119.9(8)
C(15)-C(14)-H(14)	120.1
C(14)-C(15)-H(15)	120.3
C(14)-C(15)-C(16)	119.4(9)
C(16)-C(15)-H(15)	120.3
N(3)-C(16)-C(15)	122.5(8)
N(3)-C(16)-H(16)	118.7
C(15)-C(16)-H(16)	118.7
O(4)-C(17)-Mo(1)	179.0(6)
O(5)-C(18)-Mo(1)	177.7(8)
O(6)-C(19)-Mo(1)	177.6(8)
N(6)-Mo(2)-N(4)	73.3(6)
N(6)-Mo(2)-N(5')	82.0(7)
N(6)-Mo(2)-N(4')	75.4(5)
C(36)-Mo(2)-N(6)	96.4(3)
C(36)-Mo(2)-C(38)	84.3(4)
C(36)-Mo(2)-N(5)	101.5(5)
C(36)-Mo(2)-N(4)	168.6(7)
C(36)-Mo(2)-N(5')	95.9(5)
C(36)-Mo(2)-N(4')	167.8(7)
C(37)-Mo(2)-N(6)	91.3(4)
C(37)-Mo(2)-C(36)	84.4(4)
C(37)-Mo(2)-C(38)	89.1(5)
C(37)-Mo(2)-N(5)	174.2(5)
C(37)-Mo(2)-N(4)	100.3(6)
C(37)-Mo(2)-N(5')	173.2(7)
C(37)-Mo(2)-N(4')	104.5(6)
C(38)-Mo(2)-N(6)	179.2(3)
C(38)-Mo(2)-N(5)	91.4(7)
C(38)-Mo(2)-N(4)	106.0(6)
C(38)-Mo(2)-N(5')	97.6(7)
C(38)-Mo(2)-N(4')	103.9(6)
N(5)-Mo(2)-N(6)	88.1(7)
N(5)-Mo(2)-N(4)	74.0(8)
N(5')-Mo(2)-N(4')	74.3(7)
C(31)-N(6)-Mo(2)	118.9(5)
C(35)-N(6)-Mo(2)	123.8(5)
C(35)-N(6)-C(31)	116.8(7)
N(6)-C(31)-C(32)	123.7(7)
N(6)-C(31)-C(25)	120.7(9)
N(6)-C(31)-C(25')	115.7(9)
C(32)-C(31)-C(25)	114.9(10)
C(32)-C(31)-C(25')	120.4(10)
C(31)-C(32)-H(32)	121.0
C(33)-C(32)-C(31)	118.0(8)
C(33)-C(32)-H(32)	121.0
C(32)-C(33)-H(33)	119.8
C(32)-C(33)-C(34)	120.3(8)
C(34)-C(33)-H(33)	119.8
C(33)-C(34)-H(34)	120.9
C(33)-C(34)-C(35)	118.2(9)
C(35)-C(34)-H(34)	120.9
N(6)-C(35)-C(34)	123.0(9)
N(6)-C(35)-H(35)	118.5
C(34)-C(35)-H(35)	118.5
O(10)-C(36)-Mo(2)	178.4(7)
O(11)-C(37)-Mo(2)	178.9(10)
O(12)-C(38)-Mo(2)	175.9(9)
C(20)-O(7)-H(7)	109.5
C(23)-O(8)-C(22)	101.2(11)
C(25)-O(9)-C(24)	103.8(11)
C(26)-N(5)-Mo(2)	118.4(11)

C(30)-N(5)-Mo(2)	123.6(11)	C(28)-C(27)-C(26)	119.8(15)
C(30)-N(5)-C(26)	118.0(12)	C(28)-C(27)-H(27)	120.1
C(24)-C(21)-C(22)	114.4(15)	C(27)-C(28)-H(28)	120.3
C(24)-C(21)-N(4)	102.5(13)	C(27)-C(28)-C(29)	119.4(16)
C(24)-C(21)-C(20)	111.4(18)	C(29)-C(28)-H(28)	120.3
N(4)-C(21)-C(22)	101.1(14)	C(28)-C(29)-H(29)	121.1
C(20)-C(21)-C(22)	115.4(18)	C(28)-C(29)-C(30)	117.8(16)
C(20)-C(21)-N(4)	110.7(15)	C(30)-C(29)-H(29)	121.1
O(8)-C(22)-C(21)	106.0(12)	N(5)-C(30)-C(29)	123.3(15)
O(8)-C(22)-H(22A)	110.5	N(5)-C(30)-H(30)	118.4
O(8)-C(22)-H(22B)	110.5	C(29)-C(30)-H(30)	118.4
C(21)-C(22)-H(22A)	110.5	C(21)-N(4)-Mo(2)	122.9(13)
C(21)-C(22)-H(22B)	110.5	C(23)-N(4)-Mo(2)	107.2(12)
H(22A)-C(22)-H(22B)	108.7	C(23)-N(4)-C(21)	99.5(14)
O(8)-C(23)-H(23)	109.7	C(25)-N(4)-Mo(2)	114.1(13)
O(8)-C(23)-C(26)	110.8(12)	C(25)-N(4)-C(21)	102.5(12)
O(8)-C(23)-N(4)	106.5(16)	C(25)-N(4)-C(23)	109.3(14)
C(26)-C(23)-H(23)	109.7	O(7)-C(20)-C(21)	106.3(19)
N(4)-C(23)-H(23)	109.7	O(7)-C(20)-H(20B)	110.5
N(4)-C(23)-C(26)	110.5(15)	O(7)-C(20)-H(20A)	110.5
O(9)-C(24)-C(21)	102.8(12)	C(21)-C(20)-H(20B)	110.5
O(9)-C(24)-H(24A)	111.2	C(21)-C(20)-H(20A)	110.5
O(9)-C(24)-H(24B)	111.2	H(20B)-C(20)-H(20A)	108.7
C(21)-C(24)-H(24A)	111.2	C(20')-O(7')-H(7')	109.5
C(21)-C(24)-H(24B)	111.2	C(23')-O(8')-C(22')	101.9(11)
H(24A)-C(24)-H(24B)	109.1	C(25')-O(9')-C(24')	106.3(11)
C(31)-C(25)-H(25)	111.0	C(26')-N(5')-Mo(2)	121.6(11)
O(9)-C(25)-C(31)	105.0(11)	C(30')-N(5')-Mo(2)	123.8(11)
O(9)-C(25)-H(25)	111.0	C(30')-N(5')-C(26')	114.6(11)
O(9)-C(25)-N(4)	107.6(14)	C(24')-C(21')-C(22')	113.6(15)
N(4)-C(25)-C(31)	111.0(16)	C(24')-C(21')-N(4')	103.5(13)
N(4)-C(25)-H(25)	111.0	C(24')-C(21')-C(20')	113(2)
N(5)-C(26)-C(23)	117.9(13)	N(4')-C(21')-C(22')	102.4(14)
N(5)-C(26)-C(27)	121.2(14)	N(4')-C(21')-C(20')	112.5(14)
C(27)-C(26)-C(23)	120.9(13)	C(20')-C(21')-C(22')	111(2)
C(26)-C(27)-H(27)	120.1	O(8')-C(22')-C(21')	102.6(12)

O(8')-C(22')-H(22C)	111.2	C(27')-C(26')-C(23')	119.0(13)
O(8')-C(22')-H(22D)	111.2	C(26')-C(27')-H(27')	121.5
C(21')-C(22')-H(22C)	111.2	C(28')-C(27')-C(26')	117.1(14)
C(21')-C(22')-H(22D)	111.2	C(28')-C(27')-H(27')	121.5
H(22C)-C(22')-H(22D)	109.2	C(27')-C(28')-H(28')	120.4
O(8')-C(23')-H(23')	107.1	C(27')-C(28')-C(29')	119.2(15)
O(8')-C(23')-C(26')	108.5(11)	C(29')-C(28')-H(28')	120.4
O(8')-C(23')-N(4')	110.1(15)	C(28')-C(29')-H(29')	120.3
C(26')-C(23')-H(23')	107.1	C(28')-C(29')-C(30')	119.3(16)
N(4')-C(23')-H(23')	107.1	C(30')-C(29')-H(29')	120.3
N(4')-C(23')-C(26')	116.5(14)	N(5')-C(30')-C(29')	123.6(14)
O(9')-C(24')-C(21')	105.9(13)	N(5')-C(30')-H(30')	118.2
O(9')-C(24')-H(24C)	110.6	C(29')-C(30')-H(30')	118.2
O(9')-C(24')-H(24D)	110.6	C(21')-N(4')-Mo(2)	122.5(13)
C(21')-C(24')-H(24C)	110.6	C(21')-N(4')-C(23')	102.4(13)
C(21')-C(24')-H(24D)	110.6	C(23')-N(4')-Mo(2)	110.0(12)
H(24C)-C(24')-H(24D)	108.7	C(25')-N(4')-Mo(2)	105.4(12)
C(31)-C(25')-H(25')	106.5	C(25')-N(4')-C(21')	105.4(13)
O(9')-C(25')-C(31)	117.0(12)	C(25')-N(4')-C(23')	111.0(14)
O(9')-C(25')-H(25')	106.5	O(7')-C(20')-C(21')	104.9(19)
O(9')-C(25')-N(4')	102.5(13)	O(7')-C(20')-H(20C)	110.8
N(4')-C(25')-C(31)	117.1(15)	O(7')-C(20')-H(20D)	110.8
N(4')-C(25')-H(25')	106.5	C(21')-C(20')-H(20C)	110.8
N(5')-C(26')-C(23')	115.5(12)	C(21')-C(20')-H(20D)	110.8
N(5')-C(26')-C(27')	125.5(13)	H(20C)-C(20')-H(20D)	108.8

Table S4. Anisotropic displacement parameters ($Å^2 x 10^3$) for jonap45. The anisotropic displacement factor exponent takes the form: $-2\pi^2[h^2 a^{*2}U_{11} + ... + 2 h k a^* b^* U_{12}]$

	U_{11}	U_{22}	U_{33}	U_{23}	U_{13}	U_{12}
Mo1	56(1)	44(1)	44(1)	-1(1)	3(1)	4(1)
O1	53(4)	138(7)	170(8)	19(6)	23(4)	6(4)
O2	81(4)	141(7)	72(4)	9(4)	-29(3)	-10(4)
O3	72(4)	68(4)	94(4)	31(3)	13(3)	2(3)
O4	83(4)	75(4)	71(4)	-7(3)	5(3)	-19(3)
O5	105(5)	107(5)	53(3)	19(3)	24(3)	12(4)
O6	96(5)	58(3)	109(5)	-11(3)	-4(4)	26(3)
N1	61(4)	50(3)	46(3)	1(3)	-2(3)	-5(3)
N2	62(3)	51(3)	41(3)	8(2)	12(3)	9(3)
N3	56(3)	42(3)	54(3)	-1(2)	2(3)	13(3)
C1	53(5)	109(8)	87(6)	2(6)	4(4)	10(5)
C2	53(5)	76(5)	77(5)	10(4)	-6(4)	-5(4)
C3	82(7)	102(8)	102(8)	1(6)	-23(6)	-23(6)
C4	83(6)	66(5)	54(4)	-8(4)	-4(4)	-5(4)
C5	64(6)	99(8)	122(9)	43(7)	6(5)	-10(5)
C6	78(5)	43(4)	75(5)	-3(4)	2(4)	-4(4)
C7	86(6)	58(4)	40(4)	-3(3)	-8(4)	15(4)
C8	117(8)	117(9)	45(4)	3(5)	-3(5)	-2(7)
C9	139(10)	121(10)	59(6)	34(6)	14(6)	10(8)
C10	138(9)	75(6)	60(5)	16(5)	34(6)	-1(6)
C11	82(6)	56(4)	58(4)	7(4)	12(4)	7(4)
C12	69(5)	46(4)	61(4)	4(3)	3(4)	10(3)
C13	81(6)	49(4)	88(6)	-8(4)	5(5)	13(4)
C14	97(7)	53(5)	115(8)	3(5)	1(6)	27(5)
C15	69(6)	84(7)	88(6)	1(5)	2(5)	30(5)
C16	60(5)	62(5)	64(5)	-3(4)	1(4)	10(4)
C17	63(4)	48(4)	47(4)	0(3)	9(3)	5(3)
C18	59(4)	69(5)	57(4)	5(4)	2(3)	10(4)
C19	67(5)	59(4)	53(4)	-4(3)	0(3)	-5(4)
Mo2	65(1)	43(1)	42(1)	-5(1)	9(1)	5(1)
O10	88(4)	81(4)	86(4)	-33(3)	2(3)	22(3)

O11	121(6)	150(8)	156(7)	-75(6)	60(6)	-62(6)
O12	205(8)	124(6)	47(3)	21(4)	-1(4)	16(6)
N6	78(4)	41(3)	48(3)	-2(2)	8(3)	2(3)
C31	65(4)	46(4)	47(4)	0(3)	18(3)	-6(3)
C32	78(6)	103(7)	53(4)	7(5)	32(4)	3(5)
C33	81(6)	121(9)	50(5)	29(5)	7(4)	-11(6)
C34	98(7)	77(6)	63(5)	28(5)	-10(5)	1(5)
C35	108(7)	55(5)	55(4)	14(4)	6(4)	15(5)
C36	65(5)	55(4)	54(4)	-11(3)	2(3)	1(4)
C37	77(6)	112(8)	82(6)	-37(6)	34(5)	-18(6)
C38	131(8)	64(5)	45(4)	3(4)	5(5)	16(5)
O7	95(8)	237(18)	263(19)	-25(16)	-9(10)	-11(9)
O8	77(5)	58(6)	79(6)	30(5)	21(4)	18(5)
O9	72(5)	76(7)	73(6)	19(6)	32(5)	13(5)
N5	66(4)	33(4)	38(6)	8(4)	5(4)	9(4)
C21	74(5)	71(6)	72(6)	16(5)	22(4)	15(4)
C22	76(5)	82(8)	84(8)	27(6)	22(5)	23(5)
C23	76(5)	42(4)	77(7)	22(5)	25(4)	18(4)
C24	76(6)	73(8)	87(7)	23(6)	35(6)	16(6)
C25	73(5)	33(7)	48(6)	-9(6)	25(4)	0(5)
C26	77(5)	33(4)	50(8)	11(5)	16(5)	10(4)
C27	80(8)	42(6)	118(12)	-18(8)	27(9)	9(6)
C28	79(8)	50(7)	111(8)	-26(7)	20(9)	6(7)
C29	68(7)	54(8)	112(8)	-31(9)	7(8)	8(6)
C30	67(4)	37(6)	50(8)	6(6)	-2(5)	9(4)
N4	74(4)	42(5)	50(5)	5(4)	24(4)	11(4)
C20	94(8)	101(10)	146(17)	-21(10)	-1(10)	1(8)
O7'	170(20)	150(18)	112(15)	-5(12)	-27(13)	52(15)
O8'	87(6)	56(6)	95(7)	34(5)	26(6)	24(5)
O9'	58(6)	68(7)	86(8)	24(7)	8(6)	-2(5)
N5'	64(6)	35(5)	39(7)	2(6)	4(5)	12(5)
C21'	71(7)	70(8)	71(7)	15(6)	22(6)	22(6)
C22'	78(9)	71(9)	108(11)	21(8)	19(8)	18(8)
C23'	79(9)	38(6)	73(9)	15(7)	30(7)	22(7)
C24'	76(7)	79(7)	87(9)	24(7)	20(7)	11(6)
C25'	75(8)	41(7)	41(7)	-14(7)	28(6)	-4(6)

C26'	73(7)	33(5)	41(7)	14(5)	11(6)	11(6)
C27'	75(10)	43(7)	111(10)	-22(7)	19(10)	19(8)
C28'	67(8)	47(8)	108(8)	-30(8)	14(9)	13(7)
C29'	64(9)	53(10)	107(9)	-34(10)	3(9)	15(7)
C30'	65(7)	36(8)	60(10)	-2(8)	-10(7)	11(6)
N4'	66(7)	44(6)	43(5)	9(5)	17(5)	12(6)
C20'	140(30)	240(40)	160(30)	40(30)	-30(20)	130(30)

Table S5. Hydrogen coordinates (x 10^4) and isotropic displacement parameters (Å^2 x 10^3) for jonap45.

	x	y	z	U(eq)
H1A	9913	5146	4816	179
H1B	9795	5256	3834	179
H1C	9247	4712	4939	100
H1D	9122	5263	4008	100
H3A	9643	6939	5800	116
H3B	9330	8307	5895	116
H4	8556	7627	6293	81
H5A	9273	9048	4470	114
H5B	9247	7890	3719	114
H6	8578	9069	5246	78
H8	8571	5761	7627	112
H9	8119	3898	8119	127
H10	7664	2649	7164	108
H11	7664	3179	5740	78
H13	7930	10599	4948	87
H14	7178	10648	4745	106
H15	6802	8475	4484	96
H16	7180	6301	4362	74
H32	5686	5607	9337	92
H33	6054	3616	9923	101
H34	6524	2297	9123	96
H35	6606	2995	7732	87
H7	4514	5404	6233	298
H22A	5206	8630	5480	96
H22B	5232	9636	6300	96
H23	5875	9207	7187	77
H24A	5115	8681	7613	93
H24B	4755	7481	7370	93
H25	5846	7662	8188	61
H27	6602	10463	7141	95

H28	7345	10174	7304	95
H29	7648	7909	7009	93
H30	7196	6086	6446	62
H20B	5167	5950	5569	137
H20A	5160	5095	6441	137
H7'	4869	7953	4851	217
H22C	5373	10102	6046	102
H22D	5414	9970	7050	102
H23'	6053	8823	7631	74
H24C	4944	8185	7315	96
H24D	4796	7021	6610	96
H25'	5639	7413	8111	62
H27'	6787	10229	7390	91
H28'	7520	9763	7244	89
H29'	7731	7437	6854	90
H30'	7221	5600	6707	65
H20C	5620	7356	5244	218
H20D	5219	6369	5471	218

Table S6. Torsion angles [°] for jonap45.

Mo1-N1-C2-C1	-24.3(9)	C3-O2-C4-N1	34.3(9)
Mo1-N1-C2-C3	-142.4(6)	C3-O2-C4-C7	159.4(8)
Mo1-N1-C2-C5	97.3(7)	C3-C2-C5-O3	-115.8(10)
Mo1-N1-C4-O2	121.6(6)	C4-O2-C3-C2	-44.4(10)
Mo1-N1-C4-C7	-0.5(8)	C4-N1-C2-C1	102.9(8)
Mo1-N1-C6-O3	-84.6(6)	C4-N1-C2-C3	-15.2(8)
Mo1-N1-C6-C12	36.4(7)	C4-N1-C2-C5	-135.4(8)
Mo1-N2-C7-C4	8.3(10)	C4-N1-C6-O3	153.1(6)
Mo1-N2-C7-C8	-171.9(7)	C4-N1-C6-C12	-85.9(8)
Mo1-N2-C11-C10	172.3(7)	C4-C7-C8-C9	178.9(10)
Mo1-N3-C12-C6	-0.6(9)	C5-O3-C6-N1	-47.9(8)
Mo1-N3-C12-C13	180.0(6)	C5-O3-C6-C12	-169.9(7)
Mo1-N3-C16-C15	-179.3(6)	C5-C2-C3-O2	145.3(9)
O1-C1-C2-N1	-177.2(7)	C6-O3-C5-C2	34.1(10)
O1-C1-C2-C3	-65.6(11)	C6-N1-C2-C1	-141.2(7)
O1-C1-C2-C5	67.4(10)	C6-N1-C2-C3	100.7(7)
O2-C4-C7-N2	-124.8(8)	C6-N1-C2-C5	-19.6(8)
O2-C4-C7-C8	55.4(10)	C6-N1-C4-O2	-119.1(7)
O3-C6-C12-N3	90.6(8)	C6-N1-C4-C7	118.7(7)
O3-C6-C12-C13	-90.0(9)	C6-C12-C13-C14	179.6(9)
N1-C2-C3-O2	35.6(10)	C7-N2-C11-C10	-1.6(12)
N1-C2-C5-O3	-7.4(10)	C7-C8-C9-C10	-0.6(18)
N1-C4-C7-N2	-4.8(11)	C8-C9-C10-C11	0.9(18)
N1-C4-C7-C8	175.4(8)	C9-C10-C11-N2	0.3(15)
N1-C6-C12-N3	-25.9(10)	C11-N2-C7-C4	-177.8(7)
N1-C6-C12-C13	153.5(7)	C11-N2-C7-C8	1.9(12)
N2-C7-C8-C9	-0.8(15)	C12-N3-C16-C15	1.8(12)
N3-C12-C13-C14	-1.0(14)	C12-C13-C14-C15	2.3(16)
C1-C2-C3-O2	-83.3(10)	C13-C14-C15-C16	-1.7(16)
C1-C2-C5-O3	113.7(9)	C14-C15-C16-N3	-0.5(14)
C2-N1-C4-O2	-10.1(8)	C16-N3-C12-C6	178.4(7)
C2-N1-C4-C7	-132.3(7)	C16-N3-C12-C13	-1.1(11)
C2-N1-C6-O3	41.3(7)	Mo2-N6-C31-C32	172.8(7)
C2-N1-C6-C12	162.3(7)	Mo2-N6-C31-C25	-17.2(12)

Mo2-N6-C31-C25'	-1.6(11)	O9-C25-N4-C21	14.5(17)
Mo2-N6-C35-C34	-171.2(7)	O9-C25-N4-C23	119.4(16)
Mo2-N5-C26-C23	-3(2)	N5-C26-C27-C28	-4(4)
Mo2-N5-C26-C27	179(2)	C22-O8-C23-C26	172.5(15)
Mo2-N5-C30-C29	-172(2)	C22-O8-C23-N4	52.3(18)
Mo2-N5'-C26'-C23'	8(2)	C22-C21-C24-O9	-144.6(15)
Mo2-N5'-C26'-C27'	-173(2)	C22-C21-N4-Mo2	-98.6(15)
Mo2-N5'-C30'-C29'	168(3)	C22-C21-N4-C23	19.1(17)
N6-C31-C32-C33	-1.7(14)	C22-C21-N4-C25	131.5(15)
N6-C31-C25-O9	131.2(12)	C22-C21-C20-O7	-86(3)
N6-C31-C25-N4	15.3(18)	C23-O8-C22-C21	-36.7(19)
N6-C31-C25'-O9'	101.3(15)	C23-C26-C27-C28	177(2)
N6-C31-C25'-N4'	-21.0(18)	C24-O9-C25-C31	-156.8(15)
C31-N6-C35-C34	0.4(13)	C24-O9-C25-N4	-38.5(19)
C31-C32-C33-C34	1.4(15)	C24-C21-C22-O8	118.2(18)
C31-C25-N4-Mo2	-6.3(18)	C24-C21-N4-Mo2	143.1(13)
C31-C25-N4-C21	128.8(13)	C24-C21-N4-C23	-99.2(16)
C31-C25-N4-C23	-126.3(16)	C24-C21-N4-C25	13.2(17)
C31-C25'-N4'-Mo2	29.6(17)	C24-C21-C20-O7	47(3)
C31-C25'-N4'-C21'	160.4(15)	C25-C31-C32-C33	-172.2(11)
C31-C25'-N4'-C23'	-89.5(19)	C25-O9-C24-C21	47(2)
C32-C31-C25-O9	-58.0(17)	C26-N5-C30-C29	7(3)
C32-C31-C25-N4	-174.0(13)	C26-C23-N4-Mo2	-36.8(18)
C32-C31-C25'-O9'	-73(2)	C26-C23-N4-C21	-165.8(14)
C32-C31-C25'-N4'	164.4(13)	C26-C23-N4-C25	87.3(18)
C32-C33-C34-C35	-0.4(16)	C26-C27-C28-C29	3(4)
C33-C34-C35-N6	-0.6(15)	C27-C28-C29-C30	3(4)
C35-N6-C31-C32	0.7(12)	C28-C29-C30-N5	-8(4)
C35-N6-C31-C25	170.7(11)	C30-N5-C26-C23	177.9(17)
C35-N6-C31-C25'	-173.7(10)	C30-N5-C26-C27	-1(3)
O8-C23-C26-N5	-89(2)	N4-C21-C22-O8	8.9(19)
O8-C23-C26-C27	90(2)	N4-C21-C24-O9	-36.1(18)
O8-C23-N4-Mo2	83.5(17)	N4-C21-C20-O7	160(2)
O8-C23-N4-C21	-45.4(16)	N4-C23-C26-N5	28(2)
O8-C23-N4-C25	-152.3(14)	N4-C23-C26-C27	-153(2)
O9-C25-N4-Mo2	-120.6(15)	C20-C21-C22-O8	-110.5(18)

C20-C21-C24-O9	82(2)	C24'-C21'-N4'-Mo2	110.6(15)
C20-C21-N4-Mo2	24(2)	C24'-C21'-N4'-C23'	-125.7(17)
C20-C21-N4-C23	141.9(18)	C24'-C21'-N4'-C25'	-9.6(19)
C20-C21-N4-C25	-105.7(18)	C24'-C21'-C20'-O7'	81(3)
O8'-C23'-C26'-N5'	-120.3(18)	C25'-C31-C32-C33	172.5(11)
O8'-C23'-C26'-C27'	61(2)	C25'-O9'-C24'-C21'	36(2)
O8'-C23'-N4'-Mo2	110.9(14)	C26'-N5'-C30'-C29'	-9(3)
O8'-C23'-N4'-C21'	-20.8(16)	C26'-C23'-N4'-Mo2	-13.3(19)
O8'-C23'-N4'-C25'	-132.9(15)	C26'-C23'-N4'-C21'	-144.9(14)
O9'-C25'-N4'-Mo2	-99.9(15)	C26'-C23'-N4'-C25'	103.0(16)
O9'-C25'-N4'-C21'	30.9(17)	C26'-C27'-C28'-C29'	-3(4)
O9'-C25'-N4'-C23'	141.0(14)	C27'-C28'-C29'-C30'	-1(4)
N5'-C26'-C27'-C28'	2(4)	C28'-C29'-C30'-N5'	8(4)
C22'-O8'-C23'-C26'	171.0(15)	C30'-N5'-C26'-C23'	-174.7(17)
C22'-O8'-C23'-N4'	42.4(19)	C30'-N5'-C26'-C27'	4(3)
C22'-C21'-C24'-O9'	-125.0(17)	N4'-C21'-C22'-O8'	31.9(18)
C22'-C21'-N4'-Mo2	-131.1(13)	N4'-C21'-C24'-O9'	-15(2)
C22'-C21'-N4'-C23'	-7.4(16)	N4'-C21'-C20'-O7'	-162(2)
C22'-C21'-N4'-C25'	108.7(15)	N4'-C23'-C26'-N5'	5(2)
C22'-C21'-C20'-O7'	-48(3)	N4'-C23'-C26'-C27'	-174(2)
C23'-O8'-C22'-C21'	-44.7(19)	C20'-C21'-C22'-O8'	-88(2)
C23'-C26'-C27'-C28'	-179(2)	C20'-C21'-C24'-O9'	107.3(19)
C24'-O9'-C25'-C31	-170.8(16)	C20'-C21'-N4'-Mo2	-12(3)
C24'-O9'-C25'-N4'	-41.2(19)	C20'-C21'-N4'-C23'	112(2)
C24'-C21'-C22'-O8'	142.8(16)	C20'-C21'-N4'-C25'	-132(2)

Table S7. Hydrogen bonds and close contacts for jonap45 [Å and °].

D-H...A	d(D-H)	d(H...A)	d(D...A)	<(DHA)
O1-H1A...O1#1	0.96	1.76	2.718(17)	174.9
O7-H7...O12#2	0.82	2.59	3.22(2)	134.0
O7'-H7'...O11#2	0.82	1.72	2.53(2)	171.2

Symmetry transformations used to generate equivalent atoms:

#1 -x+2,-y+1,-z+1 #2 -x+1,-y+1,-z+1

REFERENCE NUMBER: jonap46

2

CRYSTAL STRUCTURE REPORT

C_{20} H_{19} Mo N_3 O_6

or

(κ^3-FOX^{OMe})Mo$(CO)_3$

Report prepared for:

J. Li, A. Panda, Prof. W. Jones

June 29, 2024

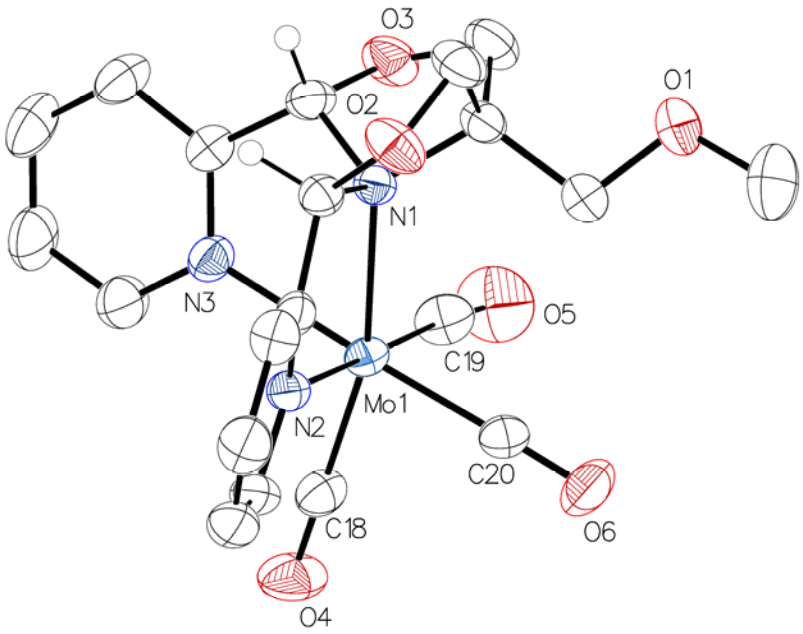

William W. Brennessel

X-ray Crystallographic Facility

Department of Chemistry, University of Rochester

120 Trustee Road

Rochester, NY 14627

Data collection

A crystal (0.098 x 0.068 x 0.011 mm^3) was placed onto a nylon loop and mounted on a Rigaku XtaLAB Synergy-S Dualflex diffractometer equipped with a HyPix-6000HE HPC area detector for data collection at 293 K. A preliminary set of cell constants and an orientation matrix were calculated from a small sampling of reflections.[1] A short pre-experiment was run, from which an optimal data collection strategy was determined. The full data collection was carried out using a PhotonJet (Cu) X-ray source with frame times of 0.36 and 1.43 seconds and a detector distance of 34.0 mm. Series of frames were collected in 0.50º steps in ω at different 2θ, κ, and ϕ settings. After the intensity data were corrected for absorption, the final cell constants were calculated from the xyz centroids of 27225 strong reflections from the actual data collection after integration.[1] See Table S8 for additional crystal and refinement information.

Structure solution and refinement

The structure was solved using SHELXT[2] and refined using SHELXL.[3] The space group $P2_1/c$ was determined based on systematic absences. Most or all non-hydrogen atoms were assigned from the solution. Full-matrix least squares / difference Fourier cycles were performed which located any remaining non-hydrogen atoms. All non-hydrogen atoms were refined with anisotropic displacement parameters. All hydrogen atoms were placed in ideal positions and refined as riding atoms with relative isotropic displacement parameters. The final full matrix least squares refinement converged to $R1 = 0.0218$ (F^2, $I > 2\sigma(I)$) and $wR2 = 0.0616$ (F^2, all data).

Structure description

The structure is the one suggested. The asymmetric unit contains one molecule in a general position.

Structure manipulation and figure generation were performed using Olex2.[4] Unless noted otherwise all structural diagrams containing anisotropic displacement ellipsoids are drawn at the 50 % probability level.

Data collection, structure solution, and structure refinement were conducted at the X-ray Crystallographic Facility, B04 Hutchison Hall, Department of Chemistry, University of Rochester. The instrument was purchased with funding from NSF MRI program grant CHE-1725028. All publications arising from this report MUST either 1) include William W. Brennessel as a coauthor or 2) acknowledge William W. Brennessel and the X-ray Crystallographic Facility of the Department of Chemistry at the University of Rochester.

1 *CrysAlisPro*, version 171.42.90a; Rigaku Corporation: Oxford, UK, 2023.

2 Sheldrick, G. M. *SHELXT* – Integrated space-group and crystal-structure determination. *Acta. Crystallogr*. **2015**, *A71*, 3-8. *SHELXT*, version 2018/2

3 Sheldrick, G. M. Crystal structure refinement with *SHELXL*. *Acta. Crystallogr.* 2015, *C71*, 3-8. *SHELXL*, version 2019/2

4 Dolomanov, O. V.; Bourhis, L. J.; Gildea, R. J.; Howard, J. A. K.; Puschmann, H. *OLEX2*: a complete structure solution, refinement and analysis program. *J. Appl. Cryst.* **2009**, *42*, 339-341. *Olex2*, version 1.5

Some equations of interest:

$$R_{int} = \Sigma |F_o^2 - \langle F_o^2 \rangle| / \Sigma |F_o^2|$$

$$R1 = \Sigma||F_o|-|F_c|| / \Sigma|F_o|$$

$$wR2 = [\Sigma[w(F_o^2-F_c^2)^2] / \Sigma [w(F_o^2)^2]]^{1/2}$$

where $w = 1 / [\sigma^2 (F_o^2) + (aP)^2 + bP]$ and

$$P = 1/3 \max (0, F_o^2) + 2/3 F_c^2$$

$$\text{GOF} = S = [\Sigma[w(F_o^2-F_c^2)^2] / (m-n)]^{1/2}$$

where m = number of reflections and n = number of parameters

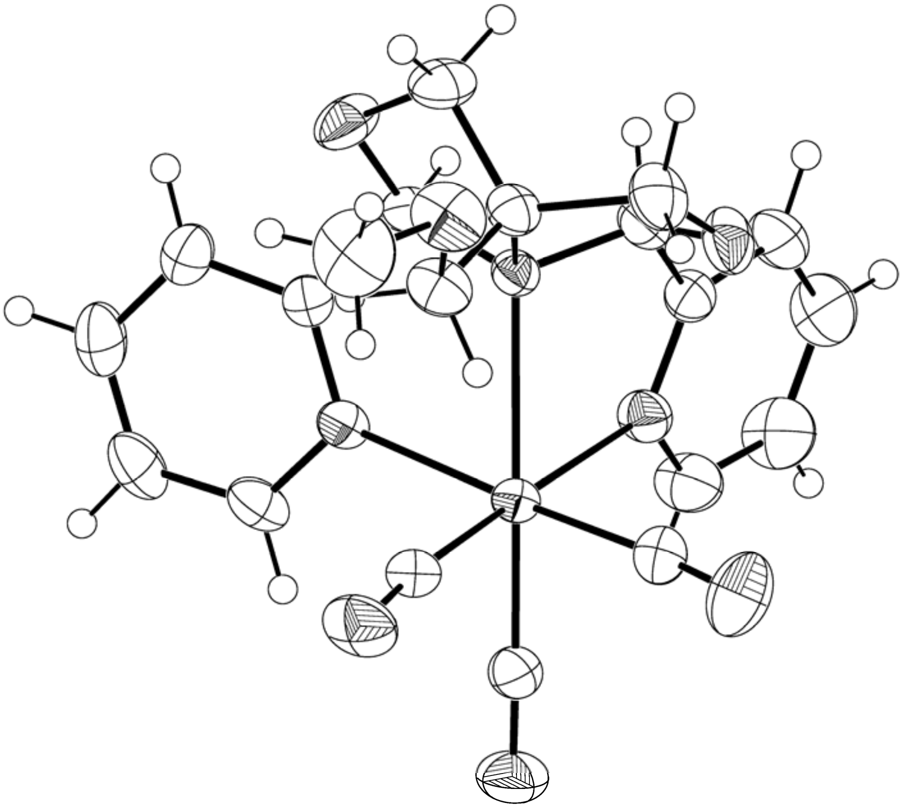

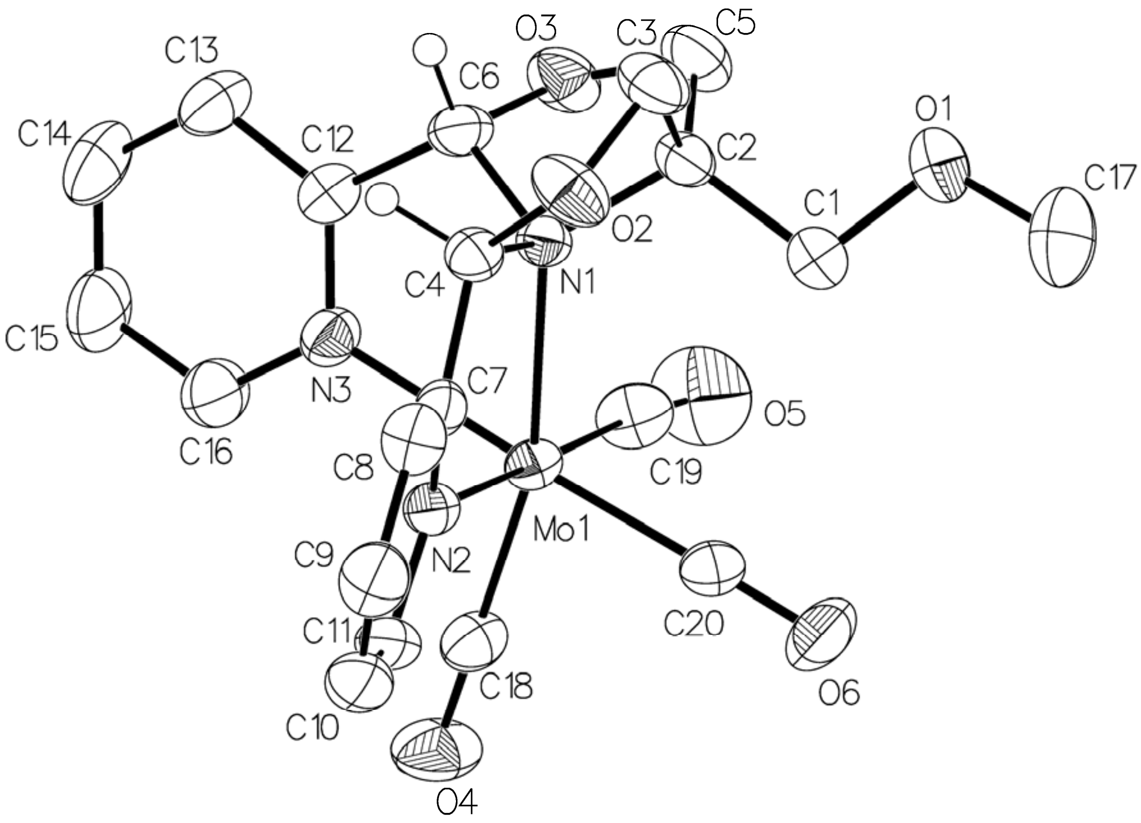
O3
C3
C5
C13
C6
O1
C14
C12
C2
C17
C1
O2
C4
N1
C15
N3
C7
O5
C16
C8
C19
Mo1
C9
N2
C20
C11
C18
O6
C10
O4

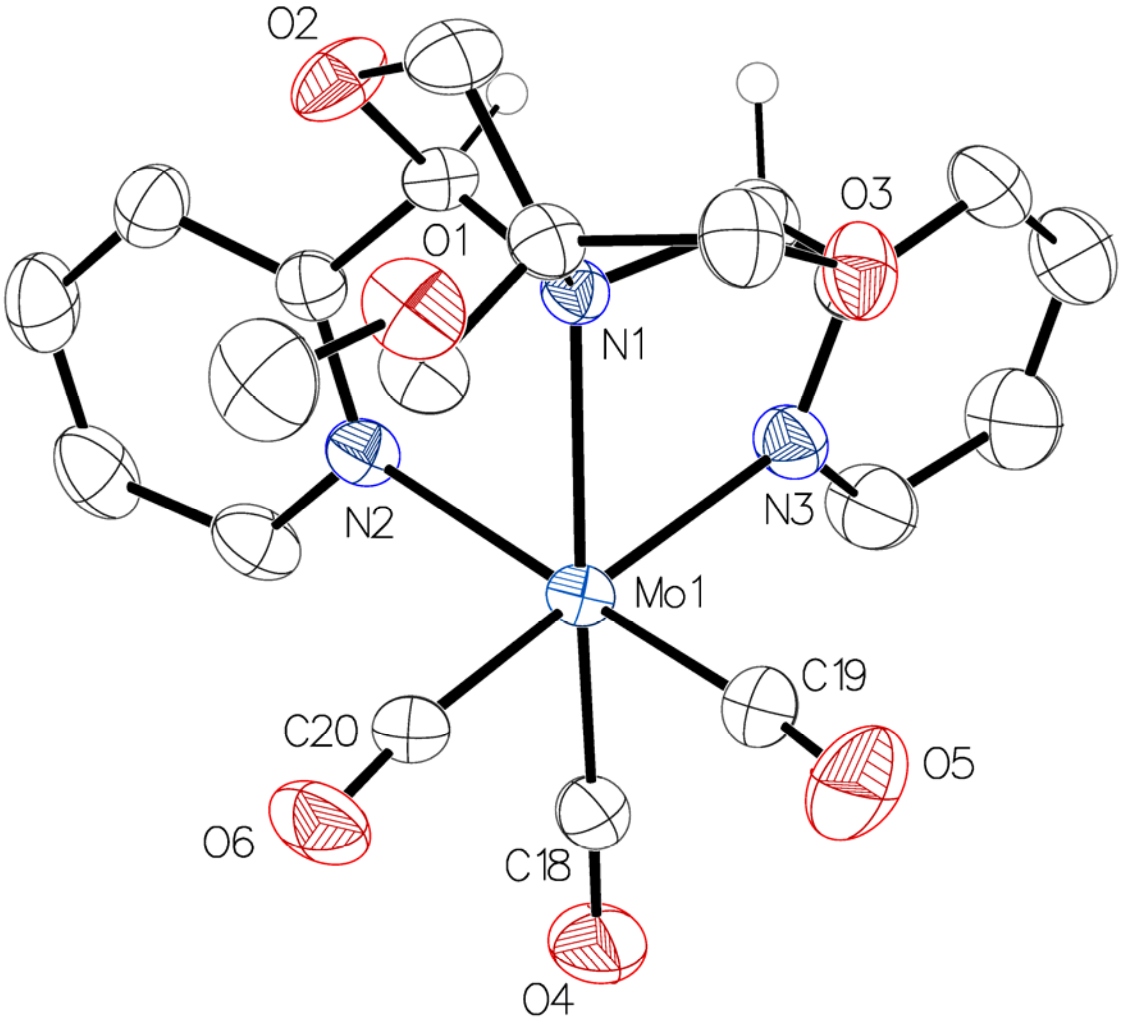
O2
O1
O3
N1
N2
N3
Mo1
C19
C20
O5
O6
C18
O4

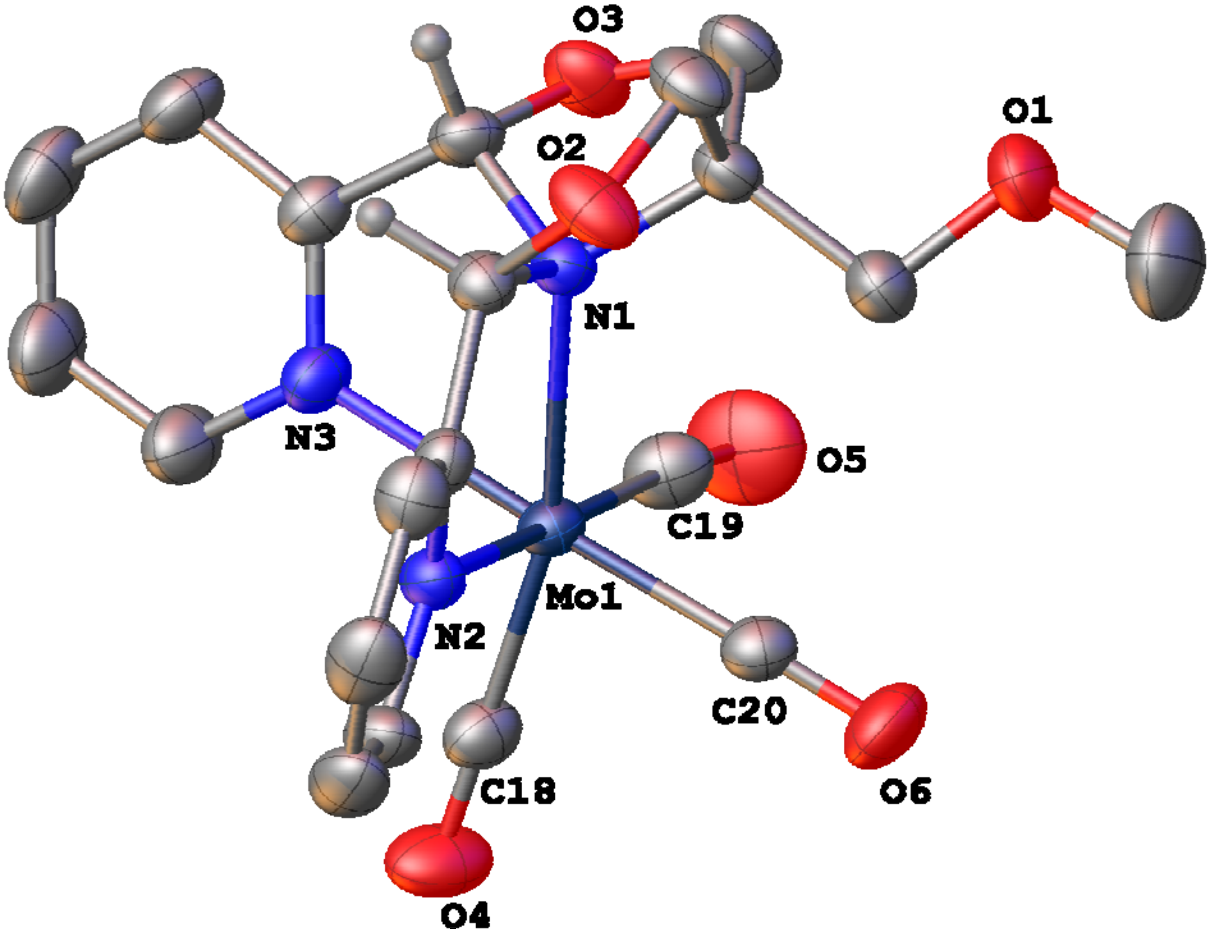
O3
O1
O2
N1
N3
O5
C19
Mo1
N2
C20
C18
O6
O4

Table S8. Crystal data and structure refinement for jonap46.

Identification code	jonap46	
Empirical formula	C20 H19 Mo N3 O6	
Formula weight	493.32	
Temperature	293 K	
Wavelength	1.54184 Å	
Crystal system	monoclinic	
Space group	$P2_1$/c	
Unit cell dimensions	*a* = 15.30526(9) Å	α = 90°
	b = 8.36787(5) Å	β = 99.8505(6)°
	c = 15.99075(10) Å	γ = 90°
Volume	2017.78(2) $Å^3$	
Z	4	
Density (calculated)	1.624 Mg/m^3	
Absorption coefficient	5.700 mm^{-1}	
F(000)	1000	
Crystal color, morphology	orange, plate	
Crystal size	0.098 x 0.068 x 0.011 mm^3	
Theta range for data collection	2.930 to 80.368°	
Index ranges	$-19 \le h \le 19$, $-10 \le k \le 10$, $-20 \le l \le 17$	
Reflections collected	35437	
Independent reflections	4359 [*R*(int) = 0.0289]	
Observed reflections	4161	
Completeness to theta = 74.504°	100.0%	
Absorption correction	Multi-scan	
Max. and min. transmission	1.00000 and 0.76242	
Refinement method	Full-matrix least-squares on F^2	
Data / restraints / parameters	4359 / 0 / 272	
Goodness-of-fit on F^2	1.081	
Final *R* indices [*I*>2sigma(*I*)]	*R*1 = 0.0218, *wR*2 = 0.0611	
R indices (all data)	*R*1 = 0.0227, *wR*2 = 0.0616	
Largest diff. peak and hole	0.335 and -0.532 e.$Å^{-3}$	

Table S9. Atomic coordinates (x 10^4) and equivalent isotropic displacement parameters ($Å^2$x 10^3) for jonap46. U_{eq} is defined as one third of the trace of the orthogonalized U_{ij} tensor.

	x	y	z	U_{eq}
Mo1	7211(1)	8271(1)	6416(1)	26(1)
O1	9524(1)	7648(2)	4626(1)	50(1)
O2	7534(1)	4919(2)	4438(1)	44(1)
O3	8994(1)	5466(2)	6858(1)	42(1)
O4	6054(1)	10552(2)	7278(1)	56(1)
O5	8819(1)	9728(3)	7610(1)	74(1)
O6	7535(1)	11296(2)	5402(1)	53(1)
N1	7824(1)	6032(2)	5805(1)	26(1)
N2	6110(1)	7250(2)	5439(1)	28(1)
N3	7063(1)	6148(2)	7246(1)	33(1)
C1	8729(1)	7734(3)	4966(1)	39(1)
C2	8640(1)	6141(2)	5394(1)	31(1)
C3	8470(1)	4816(3)	4726(1)	41(1)
C4	7146(1)	5202(2)	5165(1)	29(1)
C5	9403(1)	5828(3)	6137(1)	43(1)
C6	8149(1)	4895(2)	6522(1)	32(1)
C7	6294(1)	6114(2)	4909(1)	28(1)
C8	5718(1)	5701(2)	4170(1)	37(1)
C9	4915(1)	6485(3)	3981(2)	45(1)
C10	4704(1)	7621(3)	4538(2)	44(1)
C11	5308(1)	7965(2)	5252(1)	38(1)
C12	7566(1)	4858(2)	7185(1)	32(1)
C13	7576(2)	3544(3)	7714(1)	45(1)
C14	7059(2)	3569(3)	8343(2)	54(1)
C15	6550(2)	4892(3)	8415(2)	55(1)
C16	6557(2)	6144(3)	7854(1)	46(1)
C17	9576(2)	8810(4)	3997(2)	70(1)
C18	6501(1)	9697(2)	6960(1)	36(1)
C19	8230(1)	9119(3)	7183(1)	41(1)
C20	7400(1)	10109(2)	5742(1)	32(1)

Table S10. Bond lengths [Å] and angles [°] for jonap46.

Mo(1)-N(1)	2.3787(14)	C(8)-H(8)	0.9300
Mo(1)-N(2)	2.2612(14)	C(8)-C(9)	1.379(3)
Mo(1)-N(3)	2.2523(15)	C(9)-H(9)	0.9300
Mo(1)-C(18)	1.9201(19)	C(9)-C(10)	1.377(3)
Mo(1)-C(19)	1.945(2)	C(10)-H(10)	0.9300
Mo(1)-C(20)	1.9281(19)	C(10)-C(11)	1.372(3)
O(1)-C(1)	1.417(2)	C(11)-H(11)	0.9300
O(1)-C(17)	1.411(3)	C(12)-C(13)	1.386(3)
O(2)-C(3)	1.431(2)	C(13)-H(13)	0.9300
O(2)-C(4)	1.412(2)	C(13)-C(14)	1.382(3)
O(3)-C(5)	1.435(2)	C(14)-H(14)	0.9300
O(3)-C(6)	1.397(2)	C(14)-C(15)	1.371(4)
O(4)-C(18)	1.164(2)	C(15)-H(15)	0.9300
O(5)-C(19)	1.152(3)	C(15)-C(16)	1.380(3)
O(6)-C(20)	1.167(2)	C(16)-H(16)	0.9300
N(1)-C(2)	1.511(2)	C(17)-H(17A)	0.9600
N(1)-C(4)	1.497(2)	C(17)-H(17B)	0.9600
N(1)-C(6)	1.507(2)	C(17)-H(17C)	0.9600
N(2)-C(7)	1.336(2)	N(2)-Mo(1)-N(1)	73.63(5)
N(2)-C(11)	1.351(2)	N(3)-Mo(1)-N(1)	73.01(5)
N(3)-C(12)	1.339(2)	N(3)-Mo(1)-N(2)	88.33(6)
N(3)-C(16)	1.342(2)	C(18)-Mo(1)-N(1)	165.74(7)
C(1)-H(1A)	0.9700	C(18)-Mo(1)-N(2)	97.75(7)
C(1)-H(1B)	0.9700	C(18)-Mo(1)-N(3)	95.78(7)
C(1)-C(2)	1.516(3)	C(18)-Mo(1)-C(19)	86.72(9)
C(2)-C(3)	1.530(3)	C(18)-Mo(1)-C(20)	84.77(8)
C(2)-C(5)	1.539(3)	C(19)-Mo(1)-N(1)	102.50(7)
C(3)-H(3A)	0.9700	C(19)-Mo(1)-N(2)	174.83(7)
C(3)-H(3B)	0.9700	C(19)-Mo(1)-N(3)	93.83(8)
C(4)-H(4)	0.9800	C(20)-Mo(1)-N(1)	106.91(6)
C(4)-C(7)	1.505(2)	C(20)-Mo(1)-N(2)	94.59(7)
C(5)-H(5A)	0.9700	C(20)-Mo(1)-N(3)	176.94(7)
C(5)-H(5B)	0.9700	C(20)-Mo(1)-C(19)	83.19(8)
C(6)-H(6)	0.9800	C(17)-O(1)-C(1)	113.26(19)
C(6)-C(12)	1.499(3)	C(4)-O(2)-C(3)	106.56(14)
C(7)-C(8)	1.392(2)	C(6)-O(3)-C(5)	105.41(14)

C(2)-N(1)-Mo(1)	123.02(10)	O(3)-C(5)-C(2)	106.17(14)
C(4)-N(1)-Mo(1)	111.73(9)	O(3)-C(5)-H(5A)	110.5
C(4)-N(1)-C(2)	104.99(13)	O(3)-C(5)-H(5B)	110.5
C(4)-N(1)-C(6)	109.08(13)	C(2)-C(5)-H(5A)	110.5
C(6)-N(1)-Mo(1)	106.64(10)	C(2)-C(5)-H(5B)	110.5
C(6)-N(1)-C(2)	100.31(12)	H(5A)-C(5)-H(5B)	108.7
C(7)-N(2)-Mo(1)	119.59(11)	O(3)-C(6)-N(1)	103.89(14)
C(7)-N(2)-C(11)	117.22(16)	O(3)-C(6)-H(6)	109.5
C(11)-N(2)-Mo(1)	121.80(13)	O(3)-C(6)-C(12)	111.05(15)
C(12)-N(3)-Mo(1)	118.38(12)	N(1)-C(6)-H(6)	109.5
C(12)-N(3)-C(16)	117.46(17)	C(12)-C(6)-N(1)	113.11(14)
C(16)-N(3)-Mo(1)	123.89(14)	C(12)-C(6)-H(6)	109.5
O(1)-C(1)-H(1A)	110.5	N(2)-C(7)-C(4)	117.08(15)
O(1)-C(1)-H(1B)	110.5	N(2)-C(7)-C(8)	122.88(16)
O(1)-C(1)-C(2)	106.12(16)	C(8)-C(7)-C(4)	119.95(16)
H(1A)-C(1)-H(1B)	108.7	C(7)-C(8)-H(8)	120.7
C(2)-C(1)-H(1A)	110.5	C(9)-C(8)-C(7)	118.65(19)
C(2)-C(1)-H(1B)	110.5	C(9)-C(8)-H(8)	120.7
N(1)-C(2)-C(1)	113.22(14)	C(8)-C(9)-H(9)	120.5
N(1)-C(2)-C(3)	101.87(14)	C(10)-C(9)-C(8)	119.02(19)
N(1)-C(2)-C(5)	103.21(14)	C(10)-C(9)-H(9)	120.5
C(1)-C(2)-C(3)	109.86(16)	C(9)-C(10)-H(10)	120.5
C(1)-C(2)-C(5)	112.55(17)	C(11)-C(10)-C(9)	118.94(18)
C(3)-C(2)-C(5)	115.56(17)	C(11)-C(10)-H(10)	120.5
O(2)-C(3)-C(2)	102.81(14)	N(2)-C(11)-C(10)	123.21(19)
O(2)-C(3)-H(3A)	111.2	N(2)-C(11)-H(11)	118.4
O(2)-C(3)-H(3B)	111.2	C(10)-C(11)-H(11)	118.4
C(2)-C(3)-H(3A)	111.2	N(3)-C(12)-C(6)	117.02(15)
C(2)-C(3)-H(3B)	111.2	N(3)-C(12)-C(13)	122.76(18)
H(3A)-C(3)-H(3B)	109.1	C(13)-C(12)-C(6)	120.20(17)
O(2)-C(4)-N(1)	107.46(13)	C(12)-C(13)-H(13)	120.5
O(2)-C(4)-H(4)	108.5	C(14)-C(13)-C(12)	119.0(2)
O(2)-C(4)-C(7)	109.13(14)	C(14)-C(13)-H(13)	120.5
N(1)-C(4)-H(4)	108.5	C(13)-C(14)-H(14)	120.8
N(1)-C(4)-C(7)	114.68(13)	C(15)-C(14)-C(13)	118.5(2)
C(7)-C(4)-H(4)	108.5	C(15)-C(14)-H(14)	120.8

C(14)-C(15)-H(15)	120.3	O(1)-C(17)-H(17C)	109.5
C(14)-C(15)-C(16)	119.5(2)	H(17A)-C(17)-H(17B)	109.5
C(16)-C(15)-H(15)	120.3	H(17A)-C(17)-H(17C)	109.5
N(3)-C(16)-C(15)	122.8(2)	H(17B)-C(17)-H(17C)	109.5
N(3)-C(16)-H(16)	118.6	O(4)-C(18)-Mo(1)	178.53(19)
C(15)-C(16)-H(16)	118.6	O(5)-C(19)-Mo(1)	175.0(2)
O(1)-C(17)-H(17A)	109.5	O(6)-C(20)-Mo(1)	173.81(17)
O(1)-C(17)-H(17B)	109.5		

Table S11. Anisotropic displacement parameters ($Å^2$x 10^3) for jonap46. The anisotropic displacement factor exponent takes the form: $-2\pi^2[h^2 a^{*2}U_{11} + ... + 2 h k a^* b^* U_{12}]$

	U_{11}	U_{22}	U_{33}	U_{23}	U_{13}	U_{12}
Mo1	30(1)	22(1)	27(1)	-1(1)	4(1)	2(1)
O1	43(1)	54(1)	59(1)	9(1)	26(1)	0(1)
O2	34(1)	58(1)	40(1)	-21(1)	10(1)	0(1)
O3	29(1)	57(1)	38(1)	5(1)	0(1)	7(1)
O4	75(1)	50(1)	49(1)	-2(1)	23(1)	25(1)
O5	61(1)	82(1)	68(1)	-19(1)	-20(1)	-13(1)
O6	72(1)	30(1)	60(1)	11(1)	20(1)	1(1)
N1	24(1)	25(1)	28(1)	0(1)	4(1)	2(1)
N2	26(1)	25(1)	34(1)	0(1)	4(1)	1(1)
N3	40(1)	29(1)	32(1)	4(1)	9(1)	2(1)
C1	34(1)	39(1)	47(1)	4(1)	15(1)	2(1)
C2	25(1)	34(1)	35(1)	0(1)	7(1)	4(1)
C3	33(1)	43(1)	49(1)	-11(1)	14(1)	5(1)
C4	29(1)	25(1)	32(1)	-4(1)	6(1)	0(1)
C5	26(1)	57(1)	46(1)	4(1)	5(1)	5(1)
C6	32(1)	29(1)	35(1)	4(1)	4(1)	7(1)
C7	27(1)	26(1)	30(1)	2(1)	5(1)	-3(1)
C8	40(1)	40(1)	31(1)	-1(1)	2(1)	-6(1)
C9	37(1)	49(1)	43(1)	8(1)	-8(1)	-7(1)
C10	27(1)	41(1)	60(1)	11(1)	-3(1)	2(1)
C11	28(1)	31(1)	53(1)	2(1)	5(1)	6(1)
C12	37(1)	28(1)	31(1)	1(1)	3(1)	0(1)
C13	57(1)	31(1)	46(1)	10(1)	8(1)	6(1)
C14	76(2)	42(1)	46(1)	15(1)	15(1)	-5(1)
C15	72(2)	53(1)	47(1)	11(1)	30(1)	-3(1)
C16	58(1)	42(1)	44(1)	6(1)	23(1)	6(1)
C17	77(2)	75(2)	65(2)	17(2)	31(2)	-13(2)
C18	45(1)	31(1)	31(1)	2(1)	7(1)	4(1)
C19	45(1)	40(1)	36(1)	-2(1)	-2(1)	3(1)
C20	36(1)	28(1)	33(1)	-3(1)	6(1)	4(1)

Table S12. Hydrogen coordinates (x 10^4) and isotropic displacement parameters (Å^2 x 10^3) for jonap46.

	x	y	z	U(eq)
H1A	8226	7916	4517	47
H1B	8759	8599	5373	47
H3A	8795	5008	4265	49
H3B	8635	3778	4974	49
H4	7007	4170	5400	34
H5A	9763	4937	6008	52
H5B	9778	6765	6247	52
H6	8197	3817	6294	39
H8	5870	4912	3811	45
H9	4522	6250	3486	54
H10	4161	8146	4430	53
H11	5159	8727	5627	45
H13	7924	2659	7646	54
H14	7058	2707	8710	65
H15	6202	4947	8837	66
H16	6196	7023	7899	56
H17A	9117	8622	3518	105
H17B	10145	8745	3822	105
H17C	9503	9855	4224	105

Table S13. Torsion angles [°] for jonap46.

Mo1-N1-C2-C1	-32.5(2)	C3-O2-C4-N1	26.83(19)
Mo1-N1-C2-C3	-150.39(12)	C3-O2-C4-C7	151.75(15)
Mo1-N1-C2-C5	89.46(16)	C3-C2-C5-O3	-106.22(19)
Mo1-N1-C4-O2	133.50(11)	C4-O2-C3-C2	-40.2(2)
Mo1-N1-C4-C7	11.99(17)	C4-N1-C2-C1	96.64(17)
Mo1-N1-C6-O3	-84.33(13)	C4-N1-C2-C3	-21.26(17)
Mo1-N1-C6-C12	36.19(16)	C4-N1-C2-C5	-141.40(15)
Mo1-N2-C7-C4	19.4(2)	C4-N1-C6-O3	154.85(13)
Mo1-N2-C7-C8	-163.98(14)	C4-N1-C6-C12	-84.63(17)
Mo1-N2-C11-C10	163.80(16)	C4-C7-C8-C9	175.69(18)
Mo1-N3-C12-C6	-3.9(2)	C5-O3-C6-N1	-43.78(18)
Mo1-N3-C12-C13	174.74(16)	C5-O3-C6-C12	-165.68(16)
Mo1-N3-C16-C15	-172.84(19)	C5-C2-C3-O2	148.30(17)
O1-C1-C2-N1	179.22(15)	C6-O3-C5-C2	24.4(2)
O1-C1-C2-C3	-67.6(2)	C6-N1-C2-C1	-150.24(16)
O1-C1-C2-C5	62.7(2)	C6-N1-C2-C3	91.87(16)
O2-C4-C7-N2	-141.31(15)	C6-N1-C2-C5	-28.28(17)
O2-C4-C7-C8	42.0(2)	C6-N1-C4-O2	-108.84(15)
O3-C6-C12-N3	92.72(19)	C6-N1-C4-C7	129.65(15)
O3-C6-C12-C13	-86.0(2)	C6-C12-C13-C14	177.3(2)
N1-C2-C3-O2	37.24(19)	C7-N2-C11-C10	-2.6(3)
N1-C2-C5-O3	4.1(2)	C7-C8-C9-C10	-1.4(3)
N1-C4-C7-N2	-20.7(2)	C8-C9-C10-C11	1.6(3)
N1-C4-C7-C8	162.60(15)	C9-C10-C11-N2	0.5(3)
N1-C6-C12-N3	-23.6(2)	C11-N2-C7-C4	-173.81(16)
N1-C6-C12-C13	157.65(18)	C11-N2-C7-C8	2.8(3)
N2-C7-C8-C9	-0.8(3)	C12-N3-C16-C15	1.1(3)
N3-C12-C13-C14	-1.3(3)	C12-C13-C14-C15	0.6(4)
C1-C2-C3-O2	-83.04(18)	C13-C14-C15-C16	0.8(4)
C1-C2-C5-O3	126.46(18)	C14-C15-C16-N3	-1.8(4)
C2-N1-C4-O2	-2.06(17)	C16-N3-C12-C6	-178.21(18)
C2-N1-C4-C7	-123.57(15)	C16-N3-C12-C13	0.5(3)
C2-N1-C6-O3	44.91(16)	C17-O1-C1-C2	161.9(2)
C2-N1-C6-C12	165.42(15)		

REFERENCE NUMBER: jonap47

3

CRYSTAL STRUCTURE REPORT

C_{17} H_{19} Cl_3 Mo N_3 O_3

or

(κ^3-FOX^{OMe})MoCl$_3$ · xSolvent

Report prepared for:

J. Li, A. Panda, Prof. W. Jones

January 27, 2025

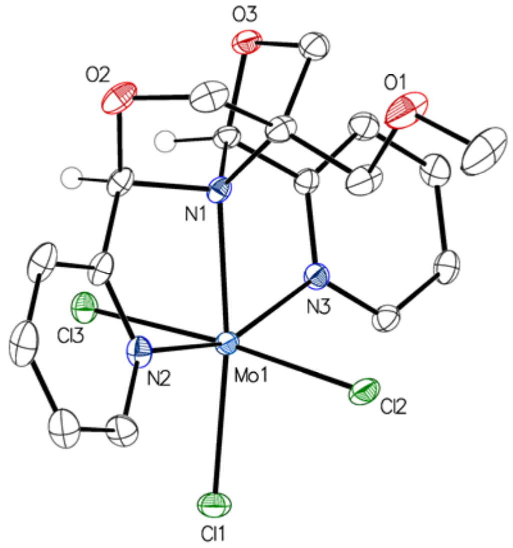

William W. Brennessel

X-ray Crystallographic Facility

Department of Chemistry, University of Rochester

120 Trustee Road

Rochester, NY 14627

Data collection

A crystal (0.177 x 0.127 x 0.047 mm^3) was placed onto a nylon loop and mounted on a Rigaku XtaLAB Synergy-S Dualflex diffractometer equipped with a HyPix-6000HE HPC area detector for data collection at 100.00(10) K. A preliminary set of cell constants and an orientation matrix were calculated from a small sampling of reflections.[1] A short pre-experiment was run, from which an optimal data collection strategy was determined. The full data collection was carried out using a PhotonJet (Cu) X-ray source with frame times of 0.17 and 0.66 seconds and a detector distance of 34.0 mm. Series of frames were collected in 0.50º steps in ω at different 2θ, κ, and ϕ settings. After the intensity data were corrected for absorption, the final cell constants were calculated from the xyz centroids of 26292 strong reflections from the actual data collection after integration.[1] See Table S14 for additional crystal and refinement information.

Structure solution and refinement

The structure was solved using SHELXT[2] and refined using SHELXL.[3] The space group *Pbca* was determined based on systematic absences. Most or all non-hydrogen atoms were assigned from the solution. Refinement proceeded in an iterative fashion, with each stage including full-matrix least squares cycles, followed by a difference Fourier synthesis, which located any remaining electron density. All non-hydrogen atoms were refined with anisotropic displacement parameters. All hydrogen atoms were placed in ideal positions and refined as riding atoms with relative isotropic displacement parameters.

Reflection contributions from highly disordered solvent were fixed and added to the calculated structure factors using the SQUEEZE routine of program Platon,[4] which determined there to be 347 electrons in 915 $Å^3$ per unit cell treated this way. Because the exact identity and amount of solvent were unknown, no solvent was included in the atom list or molecular formula. Thus all calculated quantities that derive from the molecular formula (e.g., F(000), density, molecular weight, etc.) are known to be inaccurate.

The final full matrix least squares refinement converged to $R1 = 0.0263$ (F^2, $I > 2\sigma(I)$) and $wR2 = 0.0670$ (F^2, all data).

Structure description

The structure is the one suggested. The asymmetric unit contains one molecule in a general position and solvent whose atoms were not explicitly assigned (see above).

Structure manipulation and figure generation were performed using Olex2.[5] Unless noted otherwise all structural diagrams containing anisotropic displacement ellipsoids are drawn at the 50 % probability level.

Data collection, structure solution, and structure refinement were conducted at the X-ray Crystallographic Facility, B04 Hutchison Hall, Department of Chemistry, University of Rochester. The instrument was purchased with funding from NSF MRI program grant CHE-1725028. All publications arising from this report MUST either 1) include William W. Brennessel as a coauthor or 2) acknowledge William W. Brennessel and the X-ray Crystallographic Facility of the Department of Chemistry at the University of Rochester.

[1] *CrysAlisPro*, version 171.42.90a; Rigaku Corporation: Oxford, UK, 2023.

[2] Sheldrick, G. M. *SHELXT* – Integrated space-group and crystal-structure determination. *Acta. Crystallogr.* **2015**, *A71*, 3-8. *SHELXT*, version 2018/2

[3] Sheldrick, G. M. Crystal structure refinement with *SHELXL*. *Acta. Crystallogr.* 2015, *C71*, 3-8. *SHELXL*, version 2019/2

[4] Spek, A. L. *PLATON* SQUEEZE: a tool for the calculation of the disorder solvent contribution to the calculated structure factors. *Acta. Crystallogr.* **2015**, *C71*, 9-18. *PLATON*, version 250420

[5] Dolomanov, O. V.; Bourhis, L. J.; Gildea, R. J.; Howard, J. A. K.; Puschmann, H. *OLEX2*: a complete structure solution, refinement and analysis program. *J. Appl. Cryst.* **2009**, *42*, 339-341. *Olex2*, version 1.5

Some equations of interest:

$$R_{int} = \Sigma |F_o^2 - \langle F_o^2 \rangle| / \Sigma|F_o^2|$$

$$R1 = \Sigma||F_o|-|F_c|| / \Sigma|F_o|$$

$$wR2 = [\Sigma[w(F_o^2-F_c^2)^2] / \Sigma [w(F_o^2)^2]]^{1/2}$$

where $w = 1 / [\sigma^2 (F_o^2) + (aP)^2 + bP]$ and

$$P = 1/3 \max (0, F_o^2) + 2/3 F_c^2$$

$$GOF = S = [\Sigma[w(F_o^2-F_c^2)^2] / (m-n)]^{1/2}$$

where m = number of reflections and n = number of parameters

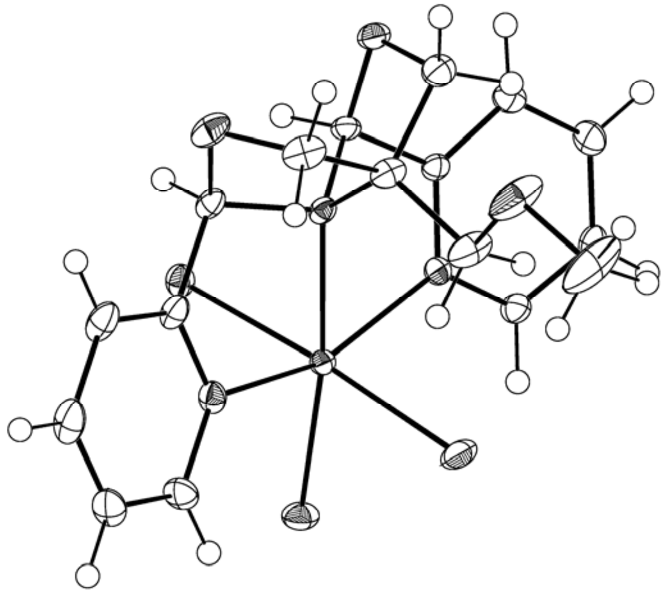

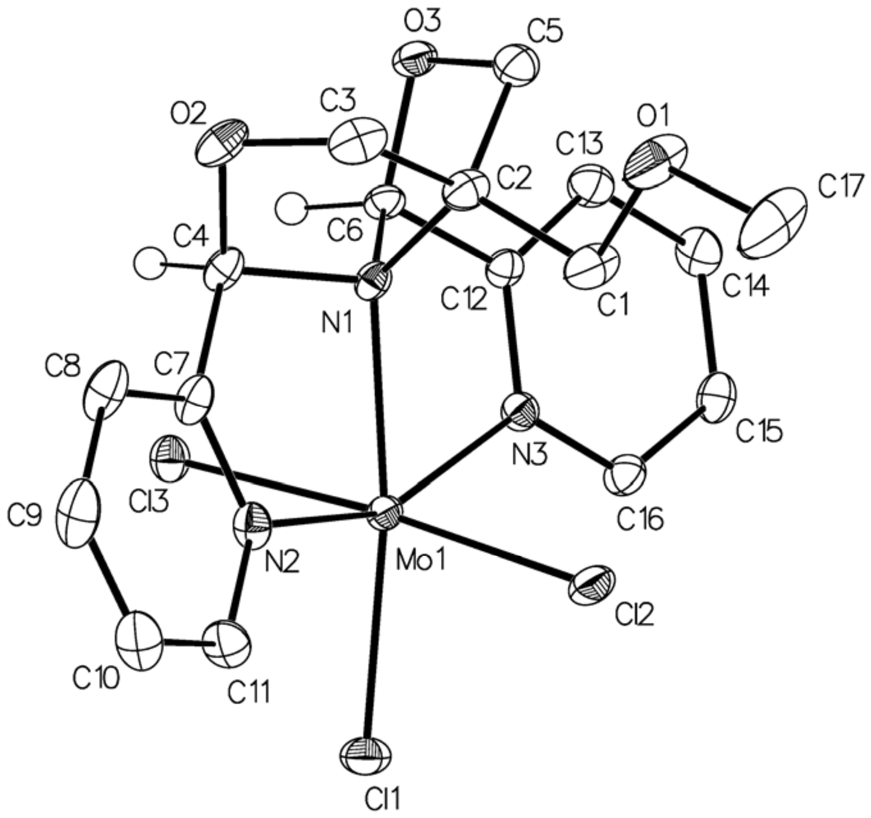
O3
C5
O2
C3
O1
C13
C2
C17
C4
C6
C12
C1
C14
N1
C8
C7
N3
C15
C9
Cl3
N2
Mo1
C16
Cl2
C10
C11
Cl1

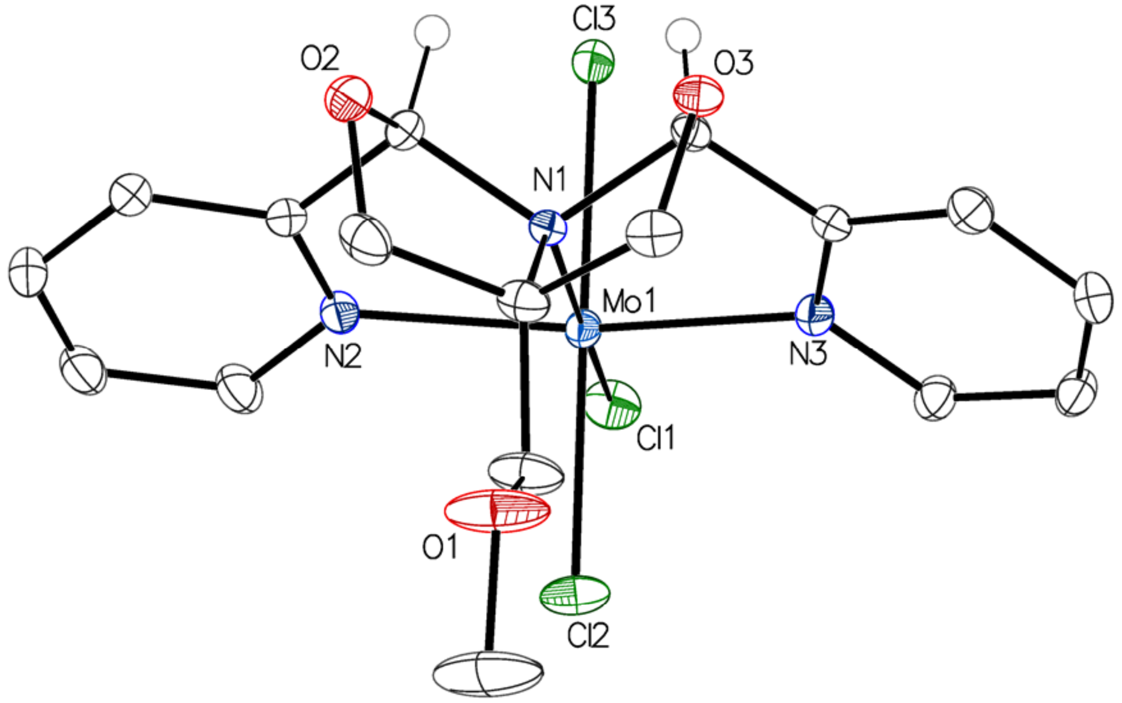
Cl3
O2
O3
N1
Mo1
N2
N3
Cl1
O1
Cl2

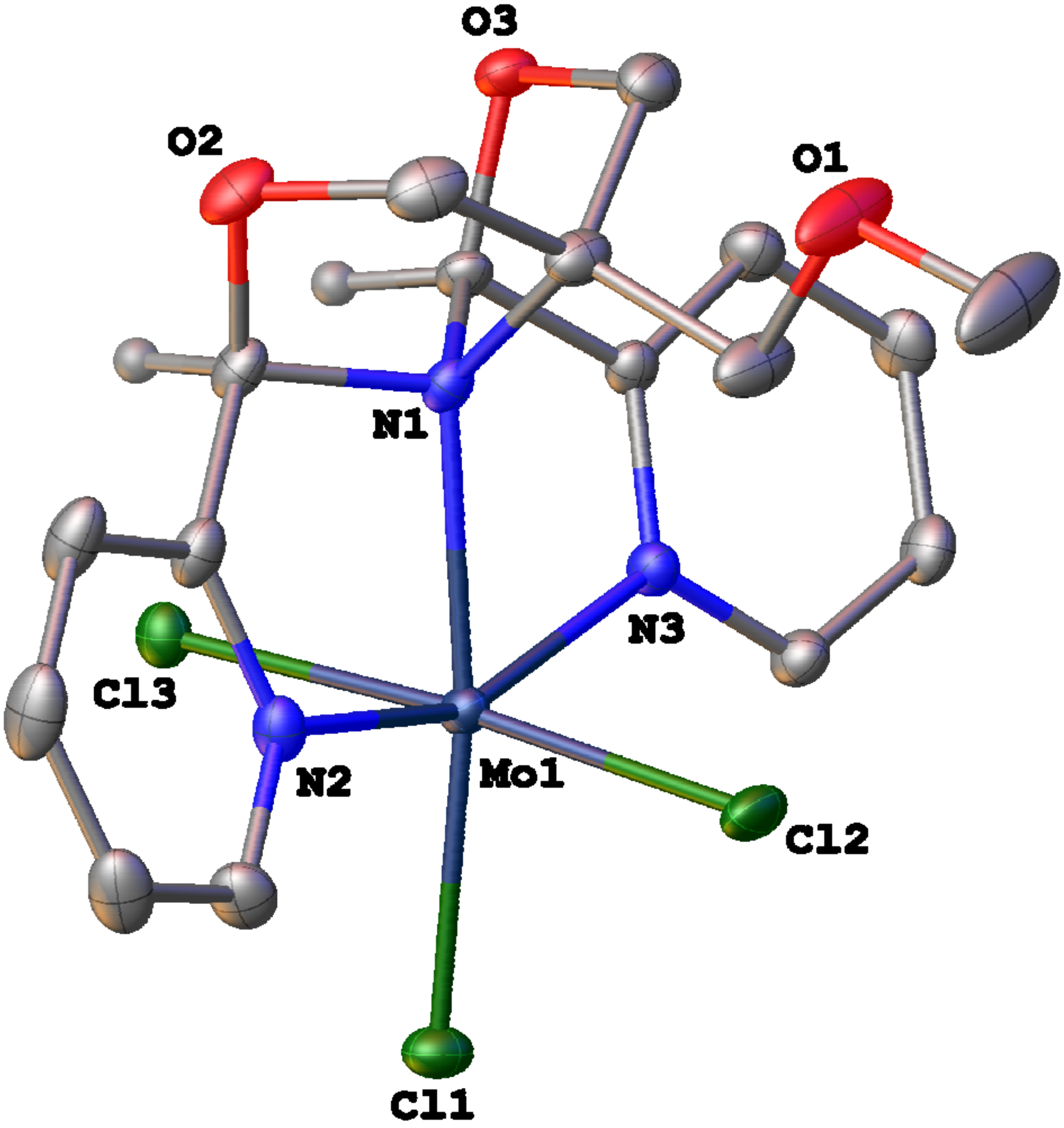
O3
O2
O1
N1
N3
Cl3
N2
Mo1
Cl2
Cl1

Table S14. Crystal data and structure refinement for jonap47.

Identification code	jonap47	
Empirical formula	C17 H19 Cl3 Mo N3 O3	
Formula weight	515.64	
Temperature	100.00(10) K	
Wavelength	1.54184 Å	
Crystal system	orthorhombic	
Space group	*Pbca*	
Unit cell dimensions	*a* = 14.50400(10) Å	α = 90°
	b = 13.68330(10) Å	β = 90°
	c = 22.84040(10) Å	γ = 90°
Volume	4532.97(5) $Å^3$	
Z	8	
Density (calculated)	1.511 Mg/m^3	
Absorption coefficient	8.170 mm^{-1}	
F(000)	2072	
Crystal color, morphology	yellow-orange, block	
Crystal size	0.177 x 0.127 x 0.047 mm^3	
Theta range for data collection	3.871 to 80.320°	
Index ranges	$-18 \leq h \leq 17$, $-12 \leq k \leq 17$, $-29 \leq l \leq 28$	
Reflections collected	40296	
Independent reflections	4883 [*R*(int) = 0.0372]	
Observed reflections	4555	
Completeness to theta = 74.504°	99.9%	
Absorption correction	Multi-scan	
Max. and min. transmission	1.00000 and 0.50520	
Refinement method	Full-matrix least-squares on F^2	
Data / restraints / parameters	4883 / 0 / 246	
Goodness-of-fit on F^2	1.068	
Final *R* indices [*I*>2sigma(*I*)]	*R*1 = 0.0263, *wR*2 = 0.0659	
R indices (all data)	*R*1 = 0.0281, *wR*2 = 0.0670	
Extinction coefficient	0.000086(13)	
Largest diff. peak and hole	0.517 and -0.567 e.$Å^{-3}$	

Table S15. Atomic coordinates (x 10^4) and equivalent isotropic displacement parameters ($Å^2$x 10^3) for jonap47. U_{eq} is defined as one third of the trace of the orthogonalized U_{ij} tensor.

	x	y	z	U_{eq}
Mo1	4636(1)	2464(1)	3012(1)	12(1)
Cl1	3690(1)	1817(1)	2239(1)	20(1)
Cl2	3518(1)	1838(1)	3688(1)	23(1)
Cl3	5699(1)	3059(1)	2254(1)	16(1)
O1	4943(1)	3022(2)	5233(1)	42(1)
O2	5988(1)	4807(1)	3902(1)	21(1)
O3	7106(1)	2894(1)	4002(1)	16(1)
N1	5612(1)	3191(1)	3649(1)	12(1)
N2	4063(1)	3897(1)	3069(1)	16(1)
N3	5557(1)	1284(1)	3225(1)	14(1)
C1	4803(2)	2800(2)	4630(1)	26(1)
C2	5601(2)	3290(2)	4322(1)	17(1)
C3	5577(2)	4406(2)	4415(1)	22(1)
C4	5623(1)	4250(1)	3443(1)	16(1)
C5	6530(2)	2843(2)	4511(1)	19(1)
C6	6518(1)	2672(1)	3532(1)	13(1)
C7	4640(1)	4576(2)	3292(1)	17(1)
C8	4371(2)	5542(2)	3356(1)	23(1)
C9	3483(2)	5807(2)	3193(1)	26(1)
C10	2894(2)	5110(2)	2973(1)	24(1)
C11	3198(2)	4160(2)	2918(1)	20(1)
C12	6349(1)	1570(1)	3487(1)	13(1)
C13	6985(2)	900(2)	3692(1)	18(1)
C14	6788(2)	-89(2)	3645(1)	20(1)
C15	5975(2)	-384(2)	3386(1)	18(1)
C16	5375(1)	319(2)	3178(1)	16(1)
C17	4197(3)	2644(3)	5576(1)	52(1)

Table S16. Bond lengths [Å] and angles [°] for jonap47.

Mo(1)-Cl(1)	2.4063(5)	C(9)-C(10)	1.377(4)
Mo(1)-Cl(2)	2.3968(5)	C(10)-H(10)	0.9500
Mo(1)-Cl(3)	2.4575(5)	C(10)-C(11)	1.378(3)
Mo(1)-N(1)	2.2603(17)	C(11)-H(11)	0.9500
Mo(1)-N(2)	2.1346(17)	C(12)-C(13)	1.383(3)
Mo(1)-N(3)	2.1514(17)	C(13)-H(13)	0.9500
O(1)-C(1)	1.426(3)	C(13)-C(14)	1.387(3)
O(1)-C(17)	1.432(3)	C(14)-H(14)	0.9500
O(2)-C(3)	1.425(3)	C(14)-C(15)	1.380(3)
O(2)-C(4)	1.401(3)	C(15)-H(15)	0.9500
O(3)-C(5)	1.434(3)	C(15)-C(16)	1.382(3)
O(3)-C(6)	1.404(2)	C(16)-H(16)	0.9500
N(1)-C(2)	1.543(3)	C(17)-H(17A)	0.9800
N(1)-C(4)	1.524(2)	C(17)-H(17B)	0.9800
N(1)-C(6)	1.517(2)	C(17)-H(17C)	0.9800
N(2)-C(7)	1.351(3)	Cl(1)-Mo(1)-Cl(3)	87.830(18)
N(2)-C(11)	1.350(3)	Cl(2)-Mo(1)-Cl(1)	87.415(18)
N(3)-C(12)	1.352(3)	Cl(2)-Mo(1)-Cl(3)	175.235(18)
N(3)-C(16)	1.350(3)	N(1)-Mo(1)-Cl(1)	172.62(4)
C(1)-H(1A)	0.9900	N(1)-Mo(1)-Cl(2)	99.62(4)
C(1)-H(1B)	0.9900	N(1)-Mo(1)-Cl(3)	85.12(4)
C(1)-C(2)	1.511(3)	N(2)-Mo(1)-Cl(1)	99.21(5)
C(2)-C(3)	1.541(3)	N(2)-Mo(1)-Cl(2)	91.46(5)
C(2)-C(5)	1.541(3)	N(2)-Mo(1)-Cl(3)	89.00(5)
C(3)-H(3A)	0.9900	N(2)-Mo(1)-N(1)	78.51(7)
C(3)-H(3B)	0.9900	N(2)-Mo(1)-N(3)	156.66(7)
C(4)-H(4)	1.0000	N(3)-Mo(1)-Cl(1)	104.12(5)
C(4)-C(7)	1.533(3)	N(3)-Mo(1)-Cl(2)	90.39(5)
C(5)-H(5A)	0.9900	N(3)-Mo(1)-Cl(3)	91.07(5)
C(5)-H(5B)	0.9900	N(3)-Mo(1)-N(1)	78.24(6)
C(6)-H(6)	1.0000	C(1)-O(1)-C(17)	110.1(2)
C(6)-C(12)	1.531(3)	C(4)-O(2)-C(3)	104.34(16)
C(7)-C(8)	1.385(3)	C(6)-O(3)-C(5)	104.80(15)
C(8)-H(8)	0.9500	C(2)-N(1)-Mo(1)	132.34(12)
C(8)-C(9)	1.388(3)	C(4)-N(1)-Mo(1)	103.05(12)
C(9)-H(9)	0.9500	C(4)-N(1)-C(2)	102.98(15)

C(6)-N(1)-Mo(1)	102.84(11)	C(2)-C(5)-H(5A)	110.7
C(6)-N(1)-C(2)	103.10(15)	C(2)-C(5)-H(5B)	110.7
C(6)-N(1)-C(4)	112.39(15)	H(5A)-C(5)-H(5B)	108.8
C(7)-N(2)-Mo(1)	114.39(14)	O(3)-C(6)-N(1)	106.84(16)
C(11)-N(2)-Mo(1)	126.24(15)	O(3)-C(6)-H(6)	109.7
C(11)-N(2)-C(7)	119.35(19)	O(3)-C(6)-C(12)	111.19(16)
C(12)-N(3)-Mo(1)	114.17(13)	N(1)-C(6)-H(6)	109.7
C(16)-N(3)-Mo(1)	126.46(14)	N(1)-C(6)-C(12)	109.52(15)
C(16)-N(3)-C(12)	118.98(18)	C(12)-C(6)-H(6)	109.7
O(1)-C(1)-H(1A)	110.9	N(2)-C(7)-C(4)	117.46(18)
O(1)-C(1)-H(1B)	110.9	N(2)-C(7)-C(8)	121.4(2)
O(1)-C(1)-C(2)	104.19(18)	C(8)-C(7)-C(4)	121.1(2)
H(1A)-C(1)-H(1B)	108.9	C(7)-C(8)-H(8)	120.6
C(2)-C(1)-H(1A)	110.9	C(7)-C(8)-C(9)	118.9(2)
C(2)-C(1)-H(1B)	110.9	C(9)-C(8)-H(8)	120.6
C(1)-C(2)-N(1)	115.54(17)	C(8)-C(9)-H(9)	120.3
C(1)-C(2)-C(3)	110.98(19)	C(10)-C(9)-C(8)	119.5(2)
C(1)-C(2)-C(5)	111.32(19)	C(10)-C(9)-H(9)	120.3
C(3)-C(2)-N(1)	102.99(17)	C(9)-C(10)-H(10)	120.4
C(5)-C(2)-N(1)	103.55(16)	C(9)-C(10)-C(11)	119.3(2)
C(5)-C(2)-C(3)	112.03(18)	C(11)-C(10)-H(10)	120.4
O(2)-C(3)-C(2)	105.01(17)	N(2)-C(11)-C(10)	121.6(2)
O(2)-C(3)-H(3A)	110.7	N(2)-C(11)-H(11)	119.2
O(2)-C(3)-H(3B)	110.7	C(10)-C(11)-H(11)	119.2
C(2)-C(3)-H(3A)	110.7	N(3)-C(12)-C(6)	116.81(17)
C(2)-C(3)-H(3B)	110.7	N(3)-C(12)-C(13)	121.65(19)
H(3A)-C(3)-H(3B)	108.8	C(13)-C(12)-C(6)	121.52(18)
O(2)-C(4)-N(1)	106.84(16)	C(12)-C(13)-H(13)	120.6
O(2)-C(4)-H(4)	109.7	C(12)-C(13)-C(14)	118.9(2)
O(2)-C(4)-C(7)	111.15(17)	C(14)-C(13)-H(13)	120.6
N(1)-C(4)-H(4)	109.7	C(13)-C(14)-H(14)	120.2
N(1)-C(4)-C(7)	109.69(16)	C(15)-C(14)-C(13)	119.7(2)
C(7)-C(4)-H(4)	109.7	C(15)-C(14)-H(14)	120.2
O(3)-C(5)-C(2)	105.29(17)	C(14)-C(15)-H(15)	120.6
O(3)-C(5)-H(5A)	110.7	C(14)-C(15)-C(16)	118.8(2)
O(3)-C(5)-H(5B)	110.7	C(16)-C(15)-H(15)	120.6

N(3)-C(16)-C(15)	122.0(2)	O(1)-C(17)-H(17C)	109.5
N(3)-C(16)-H(16)	119.0	H(17A)-C(17)-H(17B)	109.5
C(15)-C(16)-H(16)	119.0	H(17A)-C(17)-H(17C)	109.5
O(1)-C(17)-H(17A)	109.5	H(17B)-C(17)-H(17C)	109.5
O(1)-C(17)-H(17B)	109.5		

Table S17. Anisotropic displacement parameters ($Å^2$x 10^3) for jonap47. The anisotropic displacement factor exponent takes the form: $-2\pi^2[h^2 a^{*2}U_{11} + ... + 2 h k a^* b^* U_{12}]$

	U_{11}	U_{22}	U_{33}	U_{23}	U_{13}	U_{12}
Mo1	11(1)	10(1)	15(1)	0(1)	-1(1)	0(1)
Cl1	17(1)	25(1)	18(1)	-2(1)	-3(1)	-3(1)
Cl2	18(1)	31(1)	19(1)	2(1)	-1(1)	-12(1)
Cl3	17(1)	14(1)	17(1)	1(1)	2(1)	2(1)
O1	44(1)	68(1)	16(1)	-11(1)	11(1)	-36(1)
O2	19(1)	15(1)	28(1)	-8(1)	4(1)	-4(1)
O3	12(1)	19(1)	18(1)	-1(1)	-2(1)	-3(1)
N1	11(1)	11(1)	16(1)	0(1)	2(1)	0(1)
N2	16(1)	14(1)	17(1)	3(1)	3(1)	3(1)
N3	13(1)	12(1)	16(1)	1(1)	0(1)	1(1)
C1	24(1)	40(1)	15(1)	-7(1)	6(1)	-13(1)
C2	16(1)	21(1)	15(1)	-4(1)	2(1)	-4(1)
C3	19(1)	24(1)	23(1)	-10(1)	3(1)	-1(1)
C4	15(1)	11(1)	21(1)	-1(1)	3(1)	-1(1)
C5	20(1)	23(1)	15(1)	-1(1)	-1(1)	-4(1)
C6	9(1)	14(1)	17(1)	0(1)	-1(1)	0(1)
C7	16(1)	13(1)	22(1)	1(1)	6(1)	0(1)
C8	20(1)	14(1)	35(1)	2(1)	11(1)	1(1)
C9	26(1)	15(1)	38(1)	6(1)	13(1)	7(1)
C10	18(1)	26(1)	29(1)	8(1)	7(1)	9(1)
C11	15(1)	23(1)	23(1)	2(1)	1(1)	4(1)
C12	10(1)	14(1)	15(1)	1(1)	2(1)	0(1)
C13	14(1)	19(1)	22(1)	1(1)	-1(1)	1(1)
C14	17(1)	17(1)	26(1)	2(1)	1(1)	6(1)
C15	23(1)	11(1)	21(1)	1(1)	3(1)	0(1)
C16	16(1)	13(1)	20(1)	-1(1)	-1(1)	-1(1)
C17	60(2)	71(2)	24(1)	-16(1)	23(1)	-42(2)

Table S18. Hydrogen coordinates (x 10^4) and isotropic displacement parameters (Å^2 x 10^3) for jonap47.

	x	y	z	U(eq)
H1A	4207	3065	4490	31
H1B	4813	2085	4563	31
H3A	4934	4638	4460	26
H3B	5931	4591	4769	26
H4	6026	4312	3090	19
H5A	6803	3220	4838	23
H5B	6448	2157	4638	23
H6	6792	2918	3158	16
H8	4786	6014	3510	28
H9	3284	6465	3233	32
H10	2285	5281	2860	29
H11	2789	3678	2769	24
H13	7548	1114	3862	22
H14	7211	-560	3791	24
H15	5831	-1059	3350	22
H16	4817	118	2997	19
H17A	3618	2946	5449	78
H17B	4304	2793	5990	78
H17C	4159	1934	5523	78

Table S19. Torsion angles [°] for jonap47.

Mo1-N1-C2-C1	1.3(3)	C3-O2-C4-N1	-41.2(2)
Mo1-N1-C2-C3	-119.84(17)	C3-O2-C4-C7	78.4(2)
Mo1-N1-C2-C5	123.31(16)	C3-C2-C5-O3	-88.6(2)
Mo1-N1-C4-O2	163.22(12)	C4-O2-C3-C2	41.8(2)
Mo1-N1-C4-C7	42.65(17)	C4-N1-C2-C1	122.9(2)
Mo1-N1-C6-O3	-165.40(12)	C4-N1-C2-C3	1.72(19)
Mo1-N1-C6-C12	-44.87(17)	C4-N1-C2-C5	-115.13(17)
Mo1-N2-C7-C4	2.9(2)	C4-N1-C6-O3	84.46(19)
Mo1-N2-C7-C8	179.94(17)	C4-N1-C6-C12	-155.01(16)
Mo1-N2-C11-C10	179.84(16)	C4-C7-C8-C9	177.6(2)
Mo1-N3-C12-C6	-6.3(2)	C5-O3-C6-N1	40.87(19)
Mo1-N3-C12-C13	175.06(16)	C5-O3-C6-C12	-78.59(19)
Mo1-N3-C16-C15	-172.81(16)	C5-C2-C3-O2	84.9(2)
O1-C1-C2-N1	-178.5(2)	C6-O3-C5-C2	-38.9(2)
O1-C1-C2-C3	-61.7(3)	C6-N1-C2-C1	-120.1(2)
O1-C1-C2-C5	63.8(3)	C6-N1-C2-C3	118.78(17)
O2-C4-C7-N2	-151.12(18)	C6-N1-C2-C5	1.93(19)
O2-C4-C7-C8	31.9(3)	C6-N1-C4-O2	-86.77(19)
O3-C6-C12-N3	154.80(17)	C6-N1-C4-C7	152.66(17)
O3-C6-C12-C13	-26.6(3)	C6-C12-C13-C14	179.2(2)
N1-C2-C3-O2	-25.8(2)	C7-N2-C11-C10	1.4(3)
N1-C2-C5-O3	21.7(2)	C7-C8-C9-C10	0.1(4)
N1-C4-C7-N2	-33.2(3)	C8-C9-C10-C11	-0.1(4)
N1-C4-C7-C8	149.8(2)	C9-C10-C11-N2	-0.6(4)
N1-C6-C12-N3	37.0(2)	C11-N2-C7-C4	-178.45(19)
N1-C6-C12-C13	-144.41(19)	C11-N2-C7-C8	-1.4(3)
N2-C7-C8-C9	0.7(3)	C12-N3-C16-C15	-0.4(3)
N3-C12-C13-C14	-2.2(3)	C12-C13-C14-C15	1.3(3)
C1-C2-C3-O2	-150.03(18)	C13-C14-C15-C16	0.0(3)
C1-C2-C5-O3	146.44(18)	C14-C15-C16-N3	-0.4(3)
C2-N1-C4-O2	23.50(19)	C16-N3-C12-C6	-179.62(18)
C2-N1-C4-C7	-97.08(18)	C16-N3-C12-C13	1.7(3)
C2-N1-C6-O3	-25.73(19)	C17-O1-C1-C2	176.7(3)
C2-N1-C6-C12	94.80(18)		

REFERENCE NUMBER: jonap38

FOX^{OMe}

CRYSTAL STRUCTURE REPORT

$C_{17} H_{19} N_3 O_3$

Report prepared for:

A. Panda, Prof. W. Jones

February 19, 2024

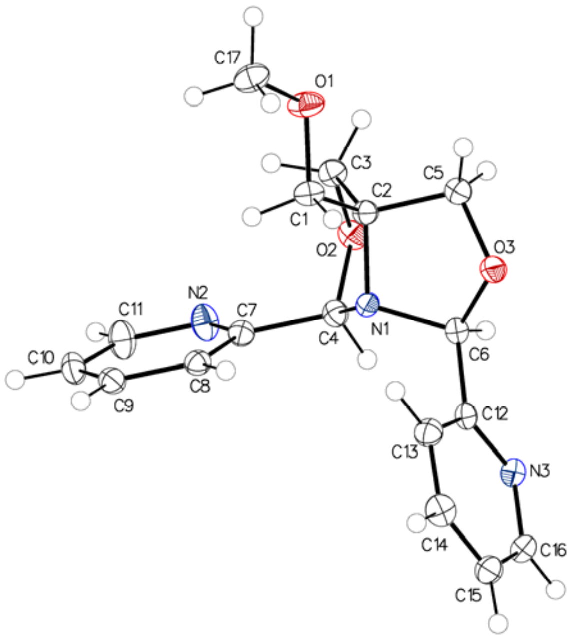

William W. Brennessel
X-ray Crystallographic Facility
Department of Chemistry, University of Rochester
120 Trustee Road
Rochester, NY 14627

Data collection

A crystal (0.183 x 0.131 x 0.084 mm^3) was placed onto a nylon loop and mounted on a Rigaku XtaLAB Synergy-S Dualflex diffractometer equipped with a HyPix-6000HE HPC area detector for data collection at 100.00(10) K. A preliminary set of cell constants and an orientation matrix were calculated from a small sampling of reflections.[1] A short pre-experiment was run, from which an optimal data collection strategy was determined. The full data collection was carried out using a PhotonJet (Cu) X-ray source with a frame time of 0.05 seconds and a detector distance of 34.0 mm. Series of frames were collected in 0.50º steps in ω at different 2θ, κ, and ϕ settings. After the intensity data were corrected for absorption, the final cell constants were calculated from the xyz centroids of 13689 strong reflections from the actual data collection after integration.[1] See Table S20 for additional crystal and refinement information.

Structure solution and refinement

The structure was solved using SHELXT[2] and refined using SHELXL.[3] The space group *I*2/*a* was determined based on systematic absences and intensity statistics. Most or all non-hydrogen atoms were assigned from the solution. Full-matrix least squares / difference Fourier cycles were performed which located any remaining non-hydrogen atoms. All non-hydrogen atoms were refined with anisotropic displacement parameters. All hydrogen atoms were placed in ideal positions and refined as riding atoms with relative isotropic displacement parameters. The final full matrix least squares refinement converged to $R1 = 0.0382$ (F^2, $I > 2\sigma(I)$) and $wR2 = 0.0923$ (F^2, all data).

Structure description

The structure is the one suggested. The asymmetric unit contains one molecule in a general position.

Structure manipulation and figure generation were performed using Olex2.[4] Unless noted otherwise all structural diagrams containing anisotropic displacement ellipsoids are drawn at the 50 % probability level.

Data collection, structure solution, and structure refinement were conducted at the X-ray Crystallographic Facility, B04 Hutchison Hall, Department of Chemistry, University of Rochester. The instrument was purchased with funding from NSF MRI program grant CHE-1725028. All publications arising from this report MUST either 1) include William W. Brennessel as a coauthor or 2) acknowledge William W. Brennessel and the X-ray Crystallographic Facility of the Department of Chemistry at the University of Rochester.

[1] *CrysAlisPro*, version 171.42.90a; Rigaku Corporation: Oxford, UK, 2023.

[2] Sheldrick, G. M. *SHELXT* – Integrated space-group and crystal-structure determination. *Acta. Crystallogr*. **2015**, *A71*, 3-8. *SHELXT*, version 2018/2

[3] Sheldrick, G. M. Crystal structure refinement with *SHELXL*. *Acta. Crystallogr.* 2015, *C71*, 3-8. *SHELXL*, version 2019/2

[4] Dolomanov, O. V.; Bourhis, L. J.; Gildea, R. J.; Howard, J. A. K.; Puschmann, H. *OLEX2*: a complete structure solution, refinement and analysis program. *J. Appl. Cryst.* **2009**, *42*, 339-341. *Olex2*, version 1.5

Some equations of interest:

$$R_{int} = \Sigma\, |F_o^2 - \langle F_o^2 \rangle| \,/\, \Sigma |F_o^2|$$

$$R1 = \Sigma ||F_o| - |F_c|| \,/\, \Sigma |F_o|$$

$$wR2 = [\Sigma[w(F_o^2 - F_c^2)^2] \,/\, \Sigma\, [w(F_o^2)^2]]^{1/2}$$

where $w = 1 / [\sigma^2 (F_o^2) + (aP)^2 + bP]$ and

$$P = 1/3 \max (0, F_o^2) + 2/3\, F_c^2$$

$$\mathrm{GOF} = S = [\Sigma[w(F_o^2 - F_c^2)^2] \,/\, (m-n)]^{1/2}$$

where m = number of reflections and n = number of parameters

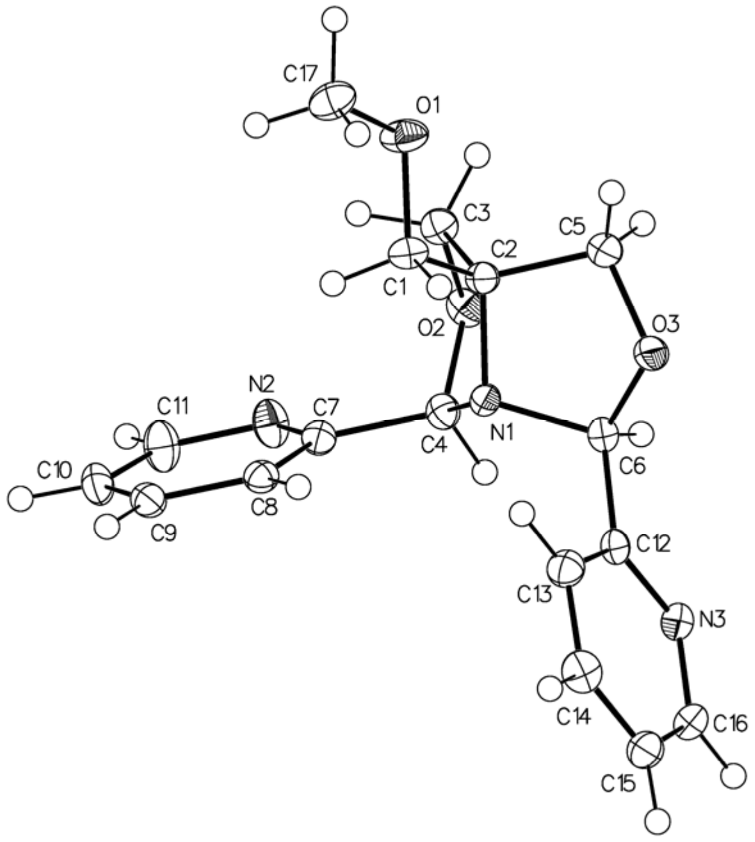
C17
O1
C3
C5
C2
C1
O2
O3
N2
C11
C7
C4
N1
C6
C10
C8
C9
C12
C13
N3
C14
C16
C15

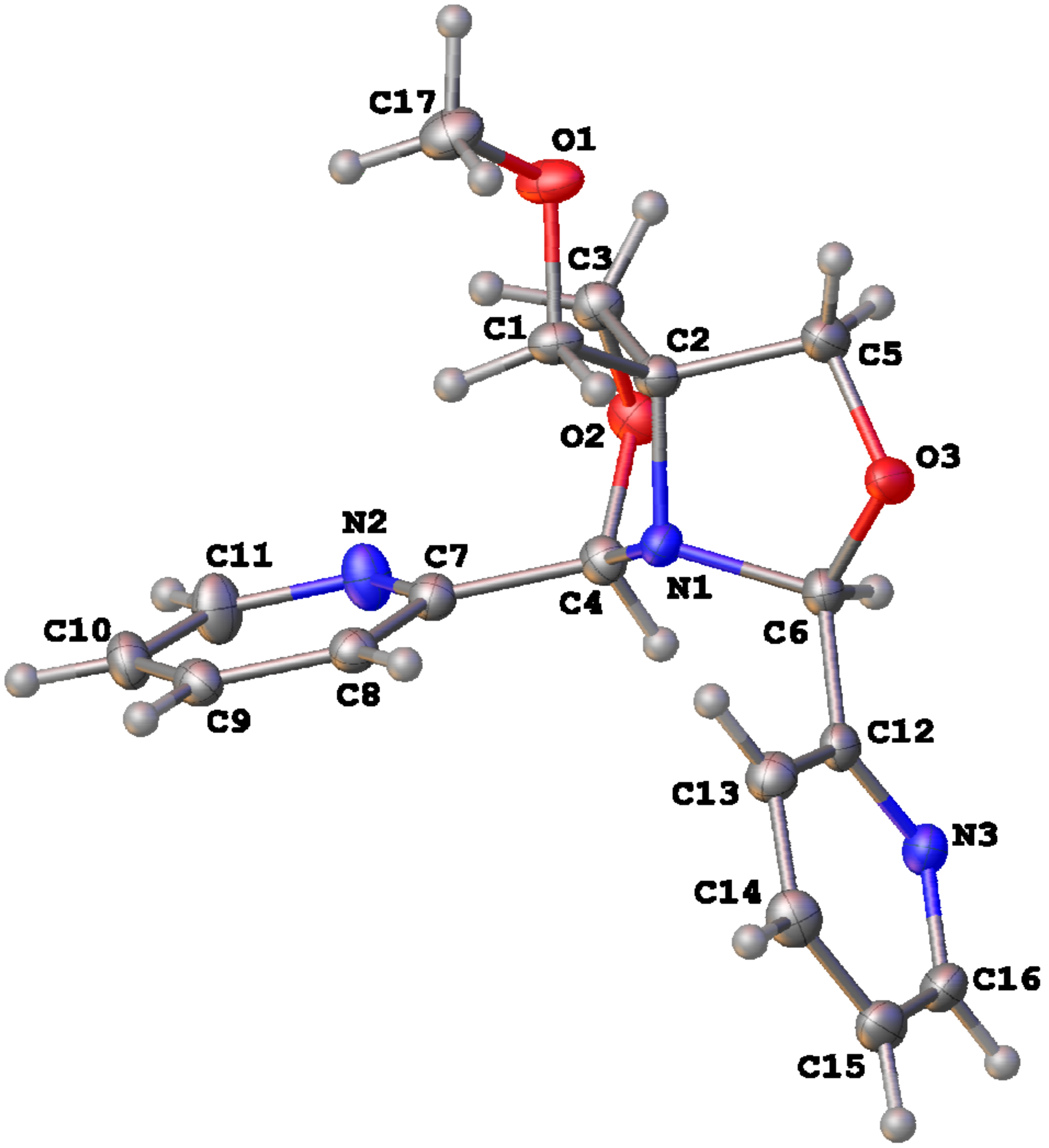

C17
O1
C3
C1
C2
C5
O2
O3
N2
C11
C7
C10
C4
N1
C6
C8
C9
C12
C13
N3
C14
C16
C15

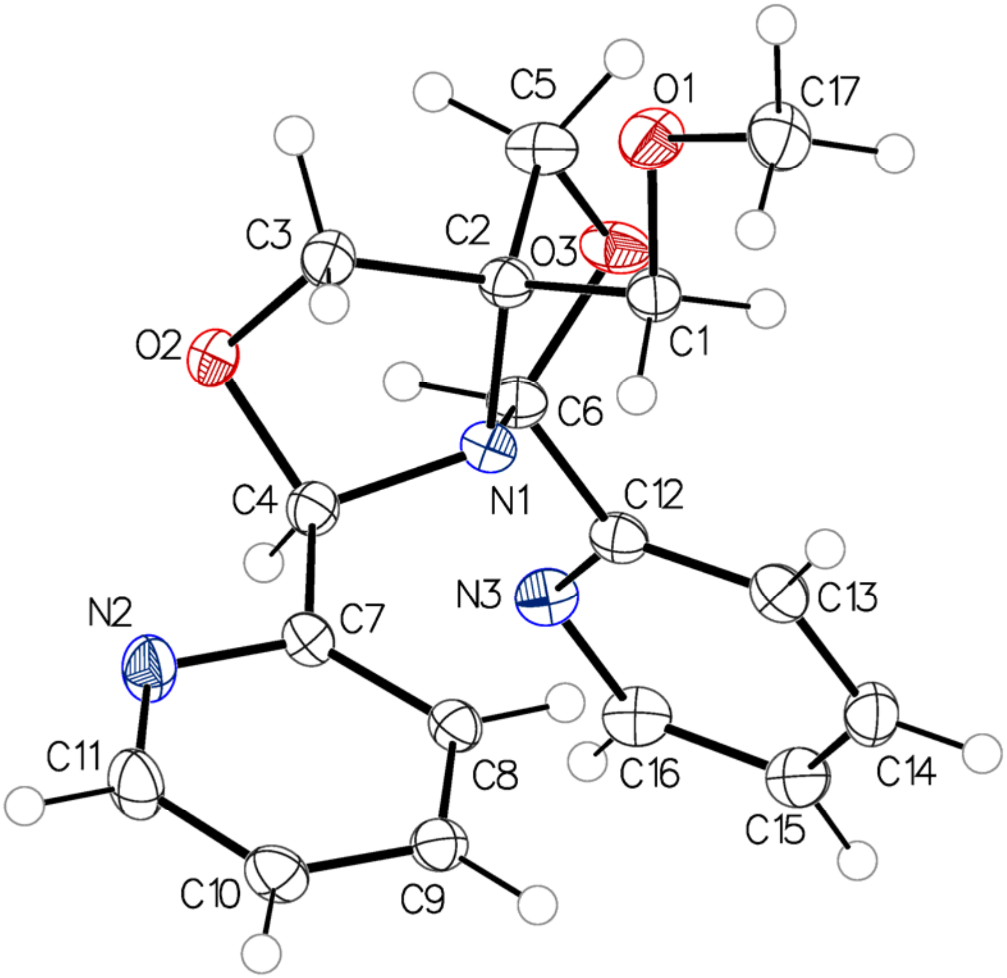
C5
O1
C17
C3
C2
O3
O2
C1
C6
N1
C4
C12
N3
C13
N2
C7
C11
C8
C16
C14
C15
C10
C9

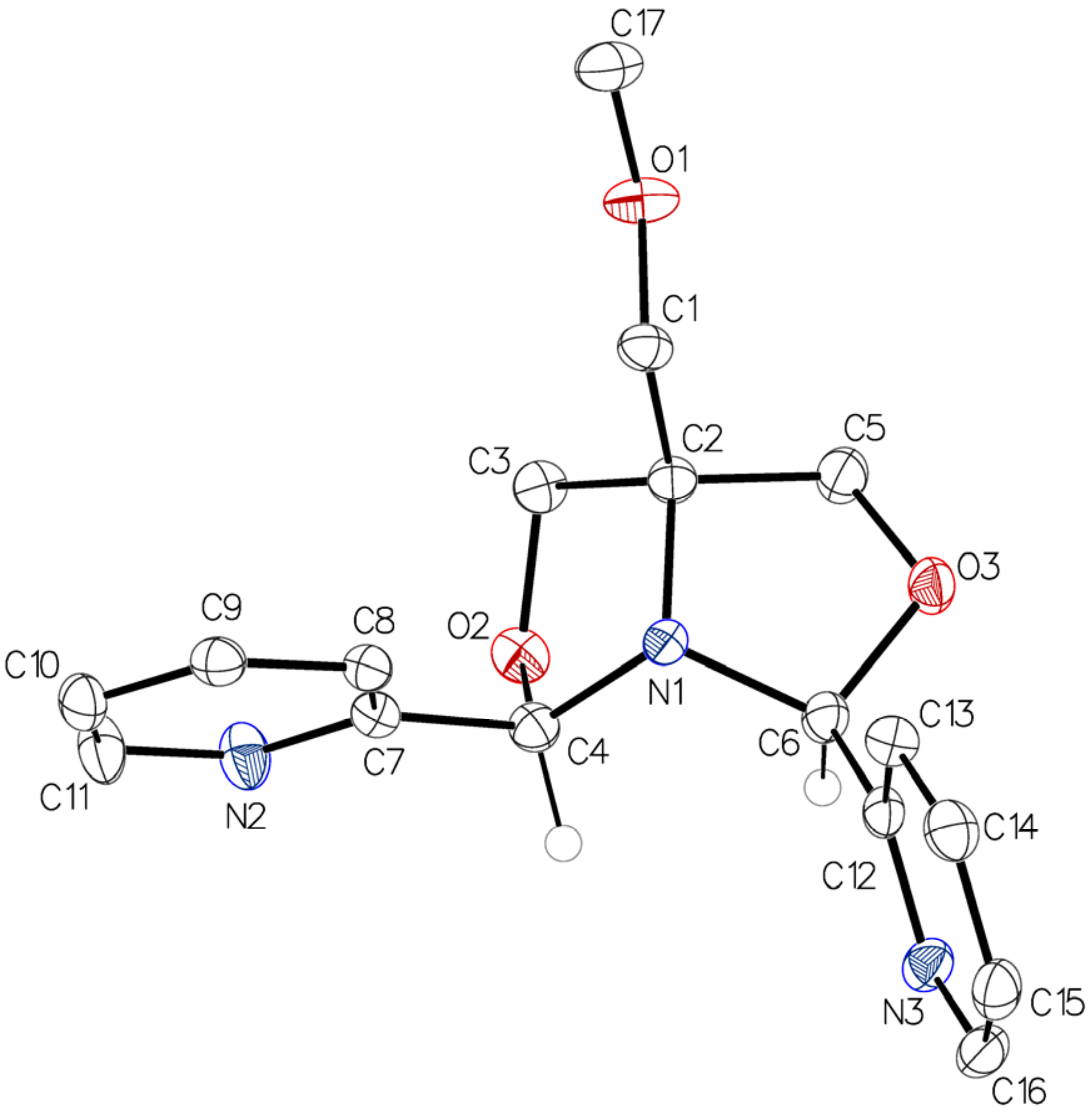

C17
O1
C1
C2
C3
C5
O3
C9
C8
O2
N1
C10
C13
C6
C7
C4
C11
N2
C14
C12
N3
C15
C16

Table S20. Crystal data and structure refinement for jonap38.

Identification code	jonap38	
Empirical formula	C17 H19 N3 O3	
Formula weight	313.35	
Temperature	100.00(10) K	
Wavelength	1.54184 Å	
Crystal system	monoclinic	
Space group	*I*2/*a*	
Unit cell dimensions	*a* = 18.68731(14) Å	α = 90°
	b = 6.21310(5) Å	β = 97.5581(7)°
	c = 27.1305(2) Å	γ = 90°
Volume	3122.65(4) $Å^3$	
Z	8	
Density (calculated)	1.333 Mg/m^3	
Absorption coefficient	0.761 mm^{-1}	
F(000)	1328	
Crystal color, morphology	colourless, block	
Crystal size	0.183 x 0.131 x 0.084 mm^3	
Theta range for data collection	3.286 to 80.266°	
Index ranges	$-23 \le h \le 17$, $-7 \le k \le 7$, $-34 \le l \le 34$	
Reflections collected	27568	
Independent reflections	3340 [*R*(int) = 0.0424]	
Observed reflections	3112	
Completeness to theta = 74.504°	99.8%	
Absorption correction	Multi-scan	
Max. and min. transmission	1.00000 and 0.91323	
Refinement method	Full-matrix least-squares on F^2	
Data / restraints / parameters	3340 / 0 / 209	
Goodness-of-fit on F^2	1.055	
Final *R* indices [*I*>2sigma(*I*)]	*R*1 = 0.0382, *wR*2 = 0.0900	
R indices (all data)	*R*1 = 0.0412, *wR*2 = 0.0923	
Largest diff. peak and hole	0.232 and -0.236 e.$Å^{-3}$	

Table S21. Atomic coordinates (x 10^4) and equivalent isotropic displacement parameters ($Å^2$x 10^3) for jonap38. U_{eq} is defined as one third of the trace of the orthogonalized U_{ij} tensor.

	x	y	z	U_{eq}
O1	6616(1)	3290(1)	7270(1)	26(1)
O2	5763(1)	-818(1)	6094(1)	21(1)
O3	4577(1)	1178(2)	7029(1)	23(1)
N1	5075(1)	2017(2)	6323(1)	16(1)
N2	5995(1)	1266(2)	5221(1)	24(1)
N3	3181(1)	1440(2)	6120(1)	20(1)
C1	6016(1)	3796(2)	6913(1)	19(1)
C2	5681(1)	1670(2)	6732(1)	17(1)
C3	6206(1)	212(2)	6495(1)	20(1)
C4	5282(1)	859(2)	5895(1)	18(1)
C5	5315(1)	520(2)	7129(1)	23(1)
C6	4420(1)	1134(2)	6504(1)	18(1)
C7	5645(1)	2319(2)	5550(1)	17(1)
C8	5598(1)	4547(2)	5566(1)	17(1)
C9	5937(1)	5757(2)	5233(1)	19(1)
C10	6307(1)	4694(2)	4898(1)	22(1)
C11	6319(1)	2462(2)	4903(1)	27(1)
C12	3756(1)	2483(2)	6353(1)	18(1)
C13	3755(1)	4671(2)	6462(1)	21(1)
C14	3129(1)	5842(2)	6331(1)	23(1)
C15	2530(1)	4784(2)	6088(1)	23(1)
C16	2580(1)	2608(2)	5989(1)	22(1)
C17	7001(1)	5155(2)	7455(1)	26(1)

Table S22. Bond lengths [Å] and angles [°] for jonap38.

O(1)-C(1)	1.4167(14)	C(12)-C(13)	1.3915(17)
O(1)-C(17)	1.4198(15)	C(13)-H(13)	0.9500
O(2)-C(3)	1.4292(14)	C(13)-C(14)	1.3840(18)
O(2)-C(4)	1.4344(14)	C(14)-H(14)	0.9500
O(3)-C(5)	1.4293(14)	C(14)-C(15)	1.3873(18)
O(3)-C(6)	1.4181(14)	C(15)-H(15)	0.9500
N(1)-C(2)	1.4931(14)	C(15)-C(16)	1.3837(18)
N(1)-C(4)	1.4620(14)	C(16)-H(16)	0.9500
N(1)-C(6)	1.4820(14)	C(17)-H(17A)	0.9800
N(2)-C(7)	1.3444(15)	C(17)-H(17B)	0.9800
N(2)-C(11)	1.3415(16)	C(17)-H(17C)	0.9800
N(3)-C(12)	1.3402(15)	C(1)-O(1)-C(17)	112.26(9)
N(3)-C(16)	1.3460(16)	C(3)-O(2)-C(4)	103.54(8)
C(1)-H(1A)	0.9900	C(6)-O(3)-C(5)	104.63(9)
C(1)-H(1B)	0.9900	C(4)-N(1)-C(2)	105.35(9)
C(1)-C(2)	1.5154(16)	C(4)-N(1)-C(6)	113.39(9)
C(2)-C(3)	1.5366(16)	C(6)-N(1)-C(2)	106.28(8)
C(2)-C(5)	1.5292(16)	C(11)-N(2)-C(7)	117.25(11)
C(3)-H(3A)	0.9900	C(12)-N(3)-C(16)	117.02(11)
C(3)-H(3B)	0.9900	O(1)-C(1)-H(1A)	110.4
C(4)-H(4)	1.0000	O(1)-C(1)-H(1B)	110.4
C(4)-C(7)	1.5245(16)	O(1)-C(1)-C(2)	106.52(9)
C(5)-H(5A)	0.9900	H(1A)-C(1)-H(1B)	108.6
C(5)-H(5B)	0.9900	C(2)-C(1)-H(1A)	110.4
C(6)-H(6)	1.0000	C(2)-C(1)-H(1B)	110.4
C(6)-C(12)	1.5078(16)	N(1)-C(2)-C(1)	110.84(9)
C(7)-C(8)	1.3878(17)	N(1)-C(2)-C(3)	103.84(9)
C(8)-H(8)	0.9500	N(1)-C(2)-C(5)	102.98(9)
C(8)-C(9)	1.3894(16)	C(1)-C(2)-C(3)	113.02(9)
C(9)-H(9)	0.9500	C(1)-C(2)-C(5)	112.42(10)
C(9)-C(10)	1.3814(17)	C(5)-C(2)-C(3)	112.90(10)
C(10)-H(10)	0.9500	O(2)-C(3)-C(2)	104.09(9)
C(10)-C(11)	1.3870(18)	O(2)-C(3)-H(3A)	110.9
C(11)-H(11)	0.9500	O(2)-C(3)-H(3B)	110.9

C(2)-C(3)-H(3A)	110.9	C(10)-C(9)-H(9)	120.7
C(2)-C(3)-H(3B)	110.9	C(9)-C(10)-H(10)	120.6
H(3A)-C(3)-H(3B)	109.0	C(9)-C(10)-C(11)	118.70(11)
O(2)-C(4)-N(1)	106.00(9)	C(11)-C(10)-H(10)	120.6
O(2)-C(4)-H(4)	109.2	N(2)-C(11)-C(10)	123.51(12)
O(2)-C(4)-C(7)	110.88(9)	N(2)-C(11)-H(11)	118.2
N(1)-C(4)-H(4)	109.2	C(10)-C(11)-H(11)	118.2
N(1)-C(4)-C(7)	112.35(9)	N(3)-C(12)-C(6)	116.04(10)
C(7)-C(4)-H(4)	109.2	N(3)-C(12)-C(13)	123.29(11)
O(3)-C(5)-C(2)	104.03(9)	C(13)-C(12)-C(6)	120.67(11)
O(3)-C(5)-H(5A)	110.9	C(12)-C(13)-H(13)	120.5
O(3)-C(5)-H(5B)	110.9	C(14)-C(13)-C(12)	118.94(11)
C(2)-C(5)-H(5A)	110.9	C(14)-C(13)-H(13)	120.5
C(2)-C(5)-H(5B)	110.9	C(13)-C(14)-H(14)	120.8
H(5A)-C(5)-H(5B)	109.0	C(13)-C(14)-C(15)	118.36(12)
O(3)-C(6)-N(1)	104.73(9)	C(15)-C(14)-H(14)	120.8
O(3)-C(6)-H(6)	110.2	C(14)-C(15)-H(15)	120.5
O(3)-C(6)-C(12)	108.43(9)	C(16)-C(15)-C(14)	119.00(12)
N(1)-C(6)-H(6)	110.2	C(16)-C(15)-H(15)	120.5
N(1)-C(6)-C(12)	112.94(9)	N(3)-C(16)-C(15)	123.38(11)
C(12)-C(6)-H(6)	110.2	N(3)-C(16)-H(16)	118.3
N(2)-C(7)-C(4)	114.35(10)	C(15)-C(16)-H(16)	118.3
N(2)-C(7)-C(8)	122.93(11)	O(1)-C(17)-H(17A)	109.5
C(8)-C(7)-C(4)	122.69(10)	O(1)-C(17)-H(17B)	109.5
C(7)-C(8)-H(8)	120.5	O(1)-C(17)-H(17C)	109.5
C(7)-C(8)-C(9)	118.95(11)	H(17A)-C(17)-H(17B)	109.5
C(9)-C(8)-H(8)	120.5	H(17A)-C(17)-H(17C)	109.5
C(8)-C(9)-H(9)	120.7	H(17B)-C(17)-H(17C)	109.5
C(10)-C(9)-C(8)	118.64(11)		

Table S23. Anisotropic displacement parameters ($Å^2 x 10^3$) for jonap38. The anisotropic displacement factor exponent takes the form: $-2\pi^2[h^2 a^{*2}U_{11} + ... + 2 h k a^* b^* U_{12}]$

	U_{11}	U_{22}	U_{33}	U_{23}	U_{13}	U_{12}
O1	23(1)	23(1)	29(1)	-3(1)	-9(1)	1(1)
O2	26(1)	14(1)	21(1)	-1(1)	2(1)	0(1)
O3	18(1)	34(1)	18(1)	7(1)	4(1)	2(1)
N1	14(1)	19(1)	16(1)	1(1)	2(1)	-2(1)
N2	33(1)	19(1)	24(1)	-2(1)	12(1)	-1(1)
N3	18(1)	21(1)	22(1)	2(1)	3(1)	-3(1)
C1	17(1)	21(1)	18(1)	-2(1)	-1(1)	2(1)
C2	16(1)	19(1)	16(1)	1(1)	1(1)	1(1)
C3	20(1)	18(1)	21(1)	-1(1)	2(1)	1(1)
C4	20(1)	16(1)	18(1)	-1(1)	2(1)	-1(1)
C5	18(1)	30(1)	21(1)	7(1)	2(1)	1(1)
C6	18(1)	20(1)	18(1)	3(1)	3(1)	-2(1)
C7	18(1)	18(1)	16(1)	-2(1)	2(1)	-1(1)
C8	17(1)	20(1)	16(1)	-1(1)	1(1)	0(1)
C9	21(1)	18(1)	18(1)	1(1)	0(1)	-2(1)
C10	26(1)	24(1)	18(1)	2(1)	6(1)	-5(1)
C11	34(1)	25(1)	24(1)	-3(1)	15(1)	-2(1)
C12	17(1)	21(1)	16(1)	3(1)	5(1)	-1(1)
C13	21(1)	23(1)	19(1)	-2(1)	2(1)	-2(1)
C14	27(1)	21(1)	24(1)	-2(1)	7(1)	2(1)
C15	18(1)	28(1)	23(1)	3(1)	6(1)	4(1)
C16	15(1)	26(1)	24(1)	3(1)	2(1)	-3(1)
C17	22(1)	27(1)	29(1)	-6(1)	-3(1)	-2(1)

Table S24. Hydrogen coordinates (x 10^4) and isotropic displacement parameters (Å^2 x 10^3) for jonap38.

	x	y	z	U(eq)
H1A	6173	4613	6633	23
H1B	5662	4677	7066	23
H3A	6592	1070	6371	24
H3B	6431	-861	6737	24
H4	4842	198	5704	21
H5A	5359	-1061	7100	28
H5B	5529	968	7467	28
H6	4335	-380	6385	22
H8	5339	5234	5800	21
H9	5915	7285	5236	23
H10	6548	5476	4668	27
H11	6570	1741	4669	32
H13	4177	5351	6624	25
H14	3110	7333	6405	28
H15	2093	5541	5991	28
H16	2168	1903	5819	26
H17A	7195	5888	7181	40
H17B	7399	4735	7708	40
H17C	6674	6128	7602	40

Table S25. Torsion angles [°] for jonap38.

O1-C1-C2-N1	-175.58(9)	C4-N1-C2-C5	-119.43(10)
O1-C1-C2-C3	-59.48(12)	C4-N1-C6-O3	138.70(9)
O1-C1-C2-C5	69.77(12)	C4-N1-C6-C12	-103.51(11)
O2-C4-C7-N2	46.53(13)	C4-C7-C8-C9	-179.08(10)
O2-C4-C7-C8	-135.26(11)	C5-O3-C6-N1	-40.35(11)
O3-C6-C12-N3	-117.43(11)	C5-O3-C6-C12	-161.17(10)
O3-C6-C12-C13	61.57(13)	C5-C2-C3-O2	87.73(11)
N1-C2-C3-O2	-23.08(11)	C6-O3-C5-C2	40.95(12)
N1-C2-C5-O3	-25.13(12)	C6-N1-C2-C1	-119.26(10)
N1-C4-C7-N2	164.95(10)	C6-N1-C2-C3	119.08(10)
N1-C4-C7-C8	-16.85(16)	C6-N1-C2-C5	1.17(12)
N1-C6-C12-N3	126.98(11)	C6-N1-C4-O2	-90.04(10)
N1-C6-C12-C13	-54.02(14)	C6-N1-C4-C7	148.71(10)
N2-C7-C8-C9	-1.02(18)	C6-C12-C13-C14	-178.10(11)
N3-C12-C13-C14	0.83(18)	C7-N2-C11-C10	-0.2(2)
C1-C2-C3-O2	-143.27(9)	C7-C8-C9-C10	0.13(17)
C1-C2-C5-O3	94.22(11)	C8-C9-C10-C11	0.65(18)
C2-N1-C4-O2	25.78(11)	C9-C10-C11-N2	-0.7(2)
C2-N1-C4-C7	-95.47(11)	C11-N2-C7-C4	179.24(11)
C2-N1-C6-O3	23.44(11)	C11-N2-C7-C8	1.04(18)
C2-N1-C6-C12	141.23(10)	C12-N3-C16-C15	-0.82(17)
C3-O2-C4-N1	-41.27(11)	C12-C13-C14-C15	-0.99(17)
C3-O2-C4-C7	80.92(10)	C13-C14-C15-C16	0.31(18)
C3-C2-C5-O3	-136.47(10)	C14-C15-C16-N3	0.63(19)
C4-O2-C3-C2	39.24(11)	C16-N3-C12-C6	179.05(10)
C4-N1-C2-C1	120.14(10)	C16-N3-C12-C13	0.07(17)
C4-N1-C2-C3	-1.52(11)	C17-O1-C1-C2	177.25(10)
